# Accessing Promising Passerini Adducts in Anticancer Drug Design

**DOI:** 10.3390/molecules29235538

**Published:** 2024-11-23

**Authors:** Ana Margarida Janeiro, Aday González-Bakker, José M. Padrón, Carolina S. Marques

**Affiliations:** 1 Faculty of Pharmacy, University of Lisbon, Av. Prof. Gama Pinto, 1649-003 Lisbon, Portugal; anamcjaneiro@gmail.com; 2 BioLab, Instituto Universitario de Bio-Orgánica Antonio González (IUBO-AG), Universidad de La Laguna, P.O. Box 456, 38200 La Laguna, Spain; agonzaba@ull.es (A.G.-B.); jmpadron@ull.edu.es (J.M.P.); 3 LAQV-REQUIMTE, Institute for Research and Advanced Studies, University of Évora, Rua Romão Ramalho, 59, 7000-641 Évora, Portugal

**Keywords:** Passerini-3C, oxindole, isatin, cancer, GI_50_, drug design

## Abstract

The 3-component Passerini reaction (3CPR), discovered little more than 100 years ago, has been demonstrated in the last few decades to be a valuable tool for accessing structural diversity and complexity, essential topics to consider in drug discovery programs. Focusing on accessing a fine-tuned family of α-acyloxyamide–oxindole hybrids, we underline herein our latest insights regarding the use of this mild reaction approach to obtain promising anticancer agents. Cheap and commercially available isatin was used as starting material. The library of α-acyloxyamide–oxindole hybrids was tested against six human solid-tumor cell lines; among them, non-small cell lung carcinoma, cervical and colon adenocarcinoma, and breast and pancreas cancer. The most potent compound displayed GI_50_ values in the range of 1.3–21 µM.

## 1. Introduction

**Figure 1:**
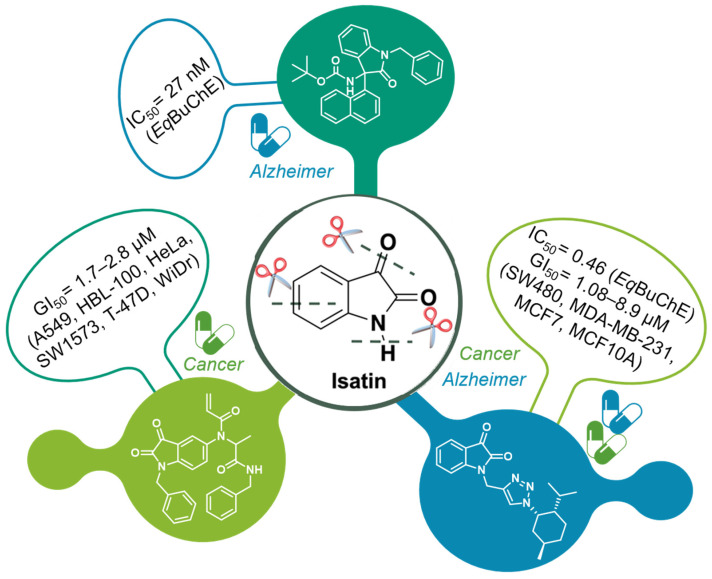
Exploiting the chemical space of isatin to access families of oxindole hybrids with biological interest in neurodegenerative diseases (like Alzheimer’s) and cancer.

Multicomponent reactions (MCRs) are a valuable tool in drug design and development programs, providing easy access to great structural diversity in mild reaction conditions with a considerable reduction in chemical waste [1,2,3]. This was demonstrated by its application in the pharmaceutical industry in the development of many commercially available drugs [4]. Compared to multistep reaction protocols, the use of MCR offers immediate advantages from a synthetic, economic, and environment point of view. Extraordinary synthetic efficiency and atom- and step economy, together with a considerable reduction in waste generation, makes these particular protocols very attractive for accessing libraries of complex molecules of biological interest (and others). In the last decade, our group has been active in the synthesis of bioactive oxindole-type hybrids, manipulating the isatin core (positions *N*1, C3 and C5 of the aromatic ring) with resourceful chemical transformations [5,6,7,8,9,10,11]. Taking advantage of the transition-metal-catalyzed arylation reactions in racemic and asymmetric fashion, libraries of 3-hydroxy-3-aryl-oxindole [5,6,9] and 3-amino-3-aryl-oxindole [8] derivatives were obtained in good to excellent yields and enantioselectivities, proving to be promising hit scaffolds as cholinesterase inhibitors [5,6,8] (important targets for neurodegenerative diseases like Alzheimer’s) and anticancer agents [9] (Figure 1). Multistep protocols were also developed in order to obtain families of 3-protected-*N*,C5-substituted-oxindole derivatives, with potent activity against butyrylcholinesterase, a well-known target involved in the study of Alzheimer’s disease [7]. The use of isatin as a component in MCR is well established in the literature [12,13], demonstrating easy access to structural diversity in the quest for new bioactive and druglike molecules.

**Scheme 1:**
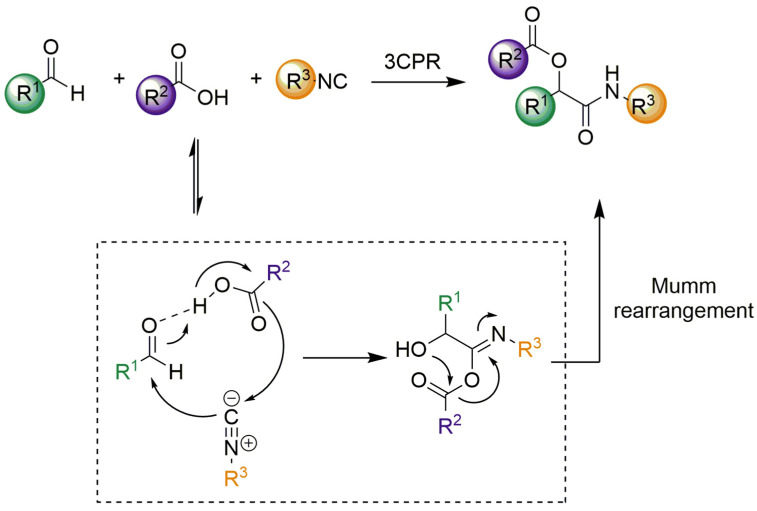
The 3CPR and general reaction mechanism.

Very recently, we reported our latest findings regarding the use of the Ugi MCR approach to obtain large libraries of isatin-based α-acetamide–carboxamide–oxindole hybrids (Figure 1), promising anticancer agents, in a mild and fast sustainable reaction process [10,11]. Focused on increasing the cytotoxicity of the drug candidates in several cancer cell lines (breast, lung, colon, etc.) and within the course of our research work–plan regarding the manipulation of the isatin core, we decided to investigate another MCR, the 3-component Passerini reaction (3CPR), to access 3,3-amido-esther-oxindole hybrids. The secular Passerini reaction was the first isocyanide-based 3-component MCR, discovered by Mario Torquato Passerini in 1921 [14], and engages an aldehyde, a carboxylic acid, and an isocyanide (Scheme 1) [15]. Overall, the most accepted mechanism starts with the activation of the aldehyde by the carboxylic acid, forming a hydrogen-bonded intermediate which undergoes the addition of an isocyanide, forming a nitrilium intermediate (α-addition of the electrophilic carbonyl component and the nucleophilic carboxylate to the chameleonic isocyanide). Mumm-type rearrangement gave access to the α-acyloxyamide product (Scheme 1). Despite being much less explored than the related Ugi 4-component reaction [16], the literature findings have demonstrated that 3CPR has emerged in the last decade as a powerful tool to access libraries of bioactive complex molecules [17]. 

However, and as far as we know, isatin-based 3CPR was still poorly explored in the literature. Eleven years ago, Esmaeili and co-workers reported the first version of the 3CPR, using isatin as ketone component. Using CH_3_CN as the solvent, at 80 °C, they could access a library of 15 isatin–Passerini adducts in moderate to high yield. [18] In the same year, the Biju group reported a similar synthetic outcome, using a solvent-free approach, at 100 °C, accessing a library of 26 isatin–Passerini adducts in low to high yield. [19] In 2016, Shaabani and co-workers explored the use of DES (Deep Eutectic Solvents) as environmental benign media to perform the 3CPR. Only two examples were reported regarding the use of isatin as a ketone component [20]. Investigating Ugi MCRs on isatin scaffolds, our group very recently reported an interesting formation of the isatin–Passerini adducts as secondary products using optimized conditions for the Ugi protocol. The cheap and commercially available InCl_3_ was used as a catalyst in mild reaction conditions [21]. Quite recently, Salami, Krause, and co-workers investigated the use of mechanochemical activation in the 3CPR. They found out that the methodology works well when using isatin as a starting material and water as a solvent. A library of 30 isatin–Passerini adducts was obtained in moderate to high yields [22,23].

Taking advantages of the MCR tool kit to obtain libraries of oxindole-type hybrids in drug design, and focused on the modification of the isatin core to increase the cytotoxicity of the scaffolds, we decided to explore the 3CPR in detail, since 3-substituted oxindole derivatives have proved their value as powerful anticancer agents [24,25].

## 2. Results and Discussion

### 2.1. The Isatin-Based 3-Component Passerini Reaction

Searching for new and powerful anticancer agents is still a very challenging and entirely relevant area of study, considering that cancer is still an incurable, complex, and fatal disease. 

The development of imatinib (Gleevec^®^) [26], the tyrosine kinase inhibitor synthetized 30 years ago and still prescribed to treat several types of cancer, marked a new era in drug discovery, setting a precedent for designing highly selective and potent drugs that achieve target-specific binding across diverse therapeutic areas. Inspired by this, we decided to explore the 3CPR’s ability to access α-acyloxyamide-oxindole hybrids **4**, using isatin derivatives **1** as starting material. Taking advantage of the scarce literature findings, we decided to start our reaction scope by using a microwave reactor, which has proven to be efficient in this kind of MCR [27]; however, as far as we know, it has not been tested with ketones. The results can be seen in Table 1.

In our first attempt, *N*-benzyl isatin **1a**, together with 2-iodobenzoic acid **2b**, *tert*-butyl isocyanide **3a**, and toluene, were put on a microwave vial at 60 °C with a short reaction time (15 min). The reaction failed to obtain the desired Passerini-adduct product **4aba** (Table 1, entry 1). Increasing the temperature, the reaction time (120 °C in 30 min), and the equivalents of **2b** (1.5 equivalents), **4aba** could be obtained in 60% yield (Table 1, entry 2). Within 3 h, **4aba** could be obtained in 91% yield, using a slight excess of **2b** (2 equivalents) and decreasing **3a** slightly (1.5 equivalents) (Table 1, entry 3). We found out that the yield of **4aba** decreased significantly when the stoichiometry of **2b** (3 equivalents) and **3a** (2.4 equivalents) were increased considerably (see Table 1; compare entries 3 and 4). Like toluene, 1,4-dioxane was also a good solvent for acquiring the desired **4aba** in very good yield (see Table 1; compare entries 3 and 5). Using the same reaction conditions to obtain 91% of **4aba** (Table 1, entry 3), we decided to evaluate the reaction scope, testing the behavior of other components (Table 1, entries 6, 8, 13 and 14). No significant differences were noted in the yield of **4aaa** using the corresponding 2-bromobenzoic acid **2a** (Table 1; compare entries 3 and 6). Using the bulky cyclohexyl isocyanide **3b**, the corresponding **4abb** was obtained at 76% (Table 1; compare entries 3 and 8). Using the unsubstituted benzoic acid **2c**, the corresponding **4acb** was obtained in 86% yield, as expected (Table 1; compare entries 8 and 13). *N*-Methyl isatin **1b** was also tested in this microwave reaction scope, and no variance in the yield was noted comparatively to the corresponding benzyl adduct **4acb** (Table 1; compare entries 13 and 14). Other variables were tested in this microwave reaction outcome. For instance, regarding the 3CPR to access **4aaa**, when an excess of **2a** (3 equivalents) and **3a** (2.4 equivalents) were used, the yield of **4aaa** slightly decreased (Table 1, entries 6 and 7). An attempt to reduce the time of the reaction was also evaluated when accessing **4abb**. The yield decreased when the reaction was performed in just 1 h (Table 1; compare entries 8 and 9). Setting the reaction time to 1 h, when the amounts of **2b** and **3b** were reduced to 1.5 and 1.2 equivalents, respectively, the yield of **4abb** was 49% (Table 1; compare entries 9 and 10). Other solvents were tested to access **4abb**, like DMF and 1,4-dioxane. Similar behavior to that reported previously for **4aba** (Table 1, entries 3 and 5) was found for **4abb** when 1,4-dioxane was used as a solvent (Table 1; compare entries 9 and 12). The use of DMF as a solvent afforded **4abb** in only 11% yield (Table 1, entry 11). In an attempt to decrease the reaction temperature, CH_3_CN was used as solvent in the synthesis of **4bcb** (Table 1, entries 15–17). Using 80 °C and 2 h of reaction time, the stoichiometric amounts of the components **2c** and **3b** were evaluated. The use of molecular sieves (MS) as an additive (water scavenger) [18] was also tested in the reaction optimization. We found similar behavior to that reported previously in the yield of **4bcb** when low and high amounts of **2c** and **3b** were used (Table 1; compare entries 15 and 17, respectively). 

Our next step was to test the reaction outcome in batch, using the Radley’s^®^ 12-position carousel system. After recognizing that the reaction does not work at room temperature (even after 7 days stirring, probably due to the less reactive and bulky ketone [15] **1**), and based on the results obtained previously using the microwave (Table 1), we start by using *N*-benzyl isatin **1a**, benzoic acid **2c**, and cyclohexylisocyanide **3b** as components, with aprotic CH_3_CN as a solvent [28] to obtain **4acb** in several reaction conditions. The results can be seen in Table 2.

The use of 2.0 equivalents of **2** and **3** was established as the optimal stoichiometric amounts for accessing high yields of **4acb** (Table 2; compare entries 1, 2 and 3). When a slight excess was used, the yield of **4acb** decreased significantly (Table 2; compare entries 5 and 14). Regarding reaction temperature and time, the reaction works better when using 100 °C and 24 h (Table 2, entry 5). No significant differences were noted by using powered or granulated 4Å MS (Table 2; compare entries 5 and 8), unlike the use of different sizes of MS (Table 2, entry 7) as water scavengers. ZnF_2_ has proven to be highly efficient as a catalyst in the 4C-Ugi reaction reported by our group. [10,11] We decided to test it in this 3CPR approach. A poor yield of 30% was obtained for **4acb**, demonstrating that the reaction works better without a catalyst (Table 2, entry 4). As demonstrated in Table 2 (entries 9 to 13), the solvent had a clear influence on reaction yield. Polar protic solvents like alcohols were not a good choice to access the Passerini adducts, as demonstrated by the low yields of **4acb** (Table 2, entries 9 and 10). In our case, the use of high polar protic 2,2,2-trifluoroethanol (TFE) afforded the desired **4acb** in 70% yield (Table 2, entry 12). According to the literature [15], typical low polarity aprotic solvents such as CH_2_Cl_2_, 2-MeTHF, or CH_3_CN are usually the ones that provide the best yields of the Passerini adducts, which was demonstrated in this study, since CH_3_CN provided the best yield of **4acb** (Table 2, entries 5 and 8). 

**Figure 2:**
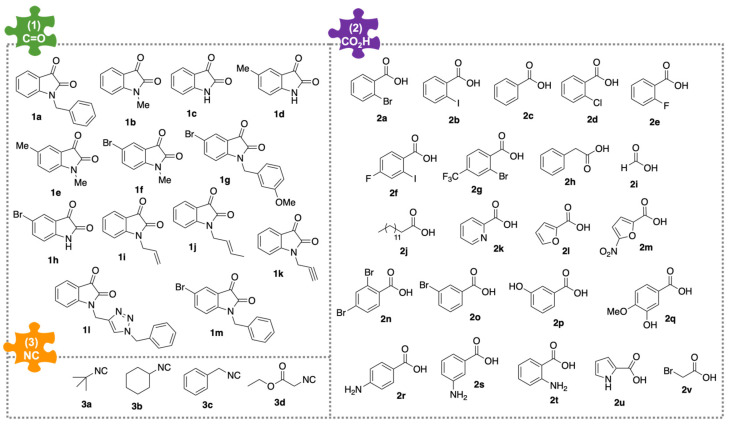
The components used in the 3CPR.

**Scheme 2:**
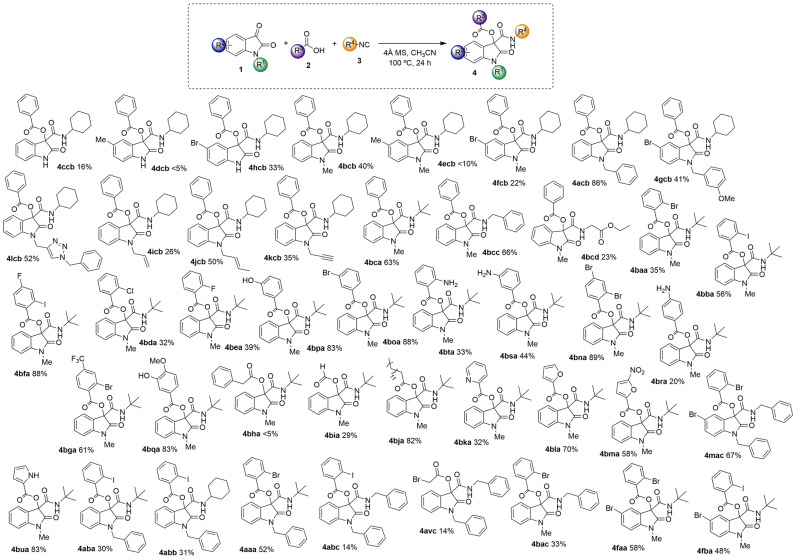
The library of α-acyloxyamide-oxindole hybrids **4** obtained via the 3CPR.

Considering the use of 2.0 equivalents of the component’s acid **2** and isocyanide **3**, and CH_3_CN (a key solvent in drug development [29]), as the optimized reaction conditions (Table 2, entry 5), we proceed with the study of the substrate scope in order to obtain a structurally versatile library of Passerini adducts **4**. A broad selection of in-house synthesized isatin derivatives **1**, commercially available carboxylic acids **2**, and isocyanides **3** were tested to achieve such a goal (Figure 2 and Scheme 2).

Using benzoic acid **2c** and cyclohexyl isocyanide **3b**, several isatin derivatives **1** were evaluated in this reaction approach. The best yield of **4** was obtained when *N*-benzyl isatin **1a** was used (Scheme 2, **4acb**, 86% yield). Usually, when *N*1-unsubstituted or C5-substituted **1** was used, very low yields of the corresponding **4** adducts were obtained (see, for instance, Scheme 2; compounds **4ccb**, **4dcb**, and **4ecb**, with 16%, <5%, and <10%, respectively). Compounds **4icb**, **4jcb**, and **4kcb** from *N*1-aliphatic derivatives of **1** were obtained in modest yields (26%, 50%, and 35%, respectively; Scheme 2). The less bulky **1b** (Figure 2) was used for isocyanide and carboxylic acid screenings. A comparison between compound **4bcb** and the compounds **4bca**, **4bcc**, and **4bcd** demonstrated the efficiency of the *tert*-butyl **3a** and benzyl **3c** isocyanides above the bulky **3b** and the electron-withdrawing **3d** (Scheme 2; a yield range from 23% to 66%). Regarding carboxylic acid **2** screening, we found out that the 3CPR has a great scope. No significant differences were found regarding *ortho*-substituted derivatives in benzoic acid derivatives **2** (Figure 2), since the yields of **4** were moderate, ranging from 32% (**4bda**) to 56% (**4bba**) (Scheme 2). The best yields were obtained when *meta*-substituted derivatives were used (**4bpa** with 83% yield and **4boa** with 88% yield) and when the benzoic acid **2** has two substituents in the aromatic ring (**4bna** with 89% yield, **4bfa** with 88% yield, and **4bqa** with 83% yield) (Scheme 2). The reaction was not efficient when 2-phenylacetic acid **2h** and formic acid **2i** were used, obtaining **4bha** and **4bia** adducts in less than 30% yield. On the contrary, using myristic acid **2j**, the corresponding **4bja** was obtained in 82% yield (Scheme 2), indicating a good tolerance for aliphatic long-chain carboxylic acids. Also, a good tolerance was established for the use of heterocyclic carboxylic acid components **2k**, **2l**, **2m** and **2u** (Figure 2), since the corresponding Passerini adducts **4bka**, **4bla**, **4bma**, and **4bua** were obtained in 32%, 70%, 58%, and 83%, respectively (Scheme 2). Interestingly, slight differences were noted in the yield of **4** depending on the position of the substituent in the aromatic ring of **2**. The formation of **4** was favored when electron-donating groups like NH_2_ or OH were present on the *meta*-position of the benzoic acid derivative **2** (see, for instance, compounds **4bsa**, **4bqa**, and **4bpa** with 44%, 83%, and 83% of yield, respectively (Scheme 2)), demonstrating electronic effects.

**Scheme 3:**
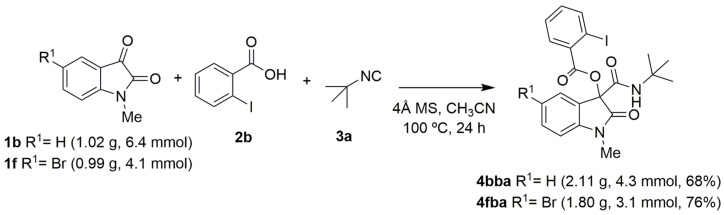
The effective gram-scale 3CPR to access **4bba** and **4fba**.

Overall, the versatility of this MCR was demonstrated by the high reagent scope, providing easy access to libraries of α-acyloxyamide-oxindole hybrids **4** in moderate to good yields (Scheme 2). To check the synthetic value of the method, the synthesis of **4bba** and **4fba** were performed at gram-scale, successfully. The yields obtained for the Passerini adducts were higher for the case of **4bba** and consistent for **4fba** (Scheme 3). 

### 2.2. Post-Passerini Reaction Approaches

**Scheme 4:**
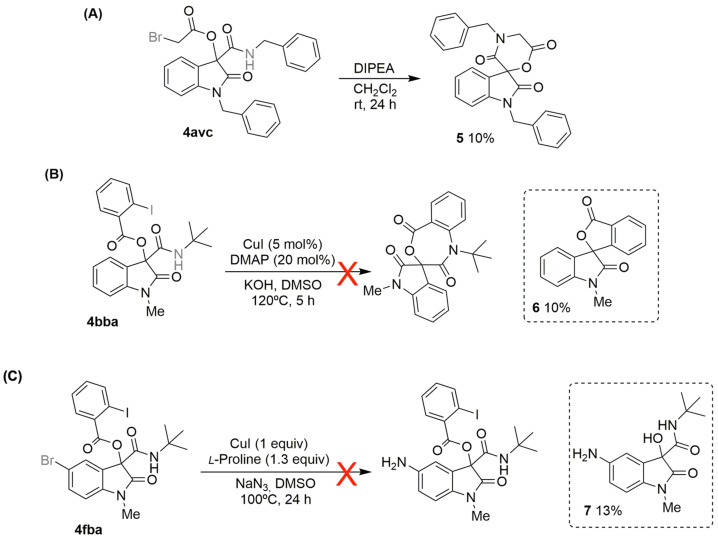
Post-Passerini reaction transformations in accessing molecular diversity; (**A**,**B**) accessing spiro-oxindole scaffold; (**C**) Cu-catalyzed amination reaction.

One of the main advantages of using MCR in drug-discovery programs is the high level of scaffold diversity and complexity, in an atom- and step-economy approach. We decided to explore the usefulness of the Passerini adducts **4** in post-Passerini reactions of interest, making them a more druglike species. Taking advantage of the bromoacetic moiety on Passerini-adduct **4avc**, we decided to use *N*,*N*’-diisopropylethylamine (DIPEA) as the non-nucleophilic base to access spiro-oxindole derivatives, privileged scaffolds for anticancer agents [25,30]. Despite not being optimized, the highly valuable spiro-oxindole **5** was obtained in 10% yield (Scheme 4A). Arylamides are also important building blocks in several biological active molecules. Cross-coupling reactions are indispensable tools and robust transformations for the formation of new C-N bonds. [31] The amination reactions of aryl halides have been impressively explored on the last decades, with the Buchwald–Hartwig [32,33] and the Ullman [34,35] catalyzed coupling reactions being remarkable examples. After several failed attempts using the Pd-catalyzed Buchwald–Hartwig system on the **4bba** Passerini adduct, we decided to use the copper-amino acid catalyzed C-N coupling methodology [36] to access the 7-member spiro-oxindole derivative (Scheme 4B). Despite the reaction having failed to reach the 7-member spiro-oxindole, a 5-member spiro-oxindole derivative **6** was obtained instead, in low yield (Scheme 4B). The mechanism is unclear, but we believe that some rearrangement might be involved in the formation of **6**.

In an attempt to obtain primary aromatic amines from aromatic halides, using sodium azide, we applied our previous established protocol [7], using the Passerini-adduct **4fba** (Scheme 4C). Despite the successful introduction of the amine unit in position C5 of the aromatic ring of **4fba**, the harsh reaction conditions led to the cleavage of the ester group of the scaffold [37], leading to the 3-hydroxy-derivative **7**, in 13% yield (Scheme 4C). 

### 2.3. Antiproliferative Activity

**Figure 3:**
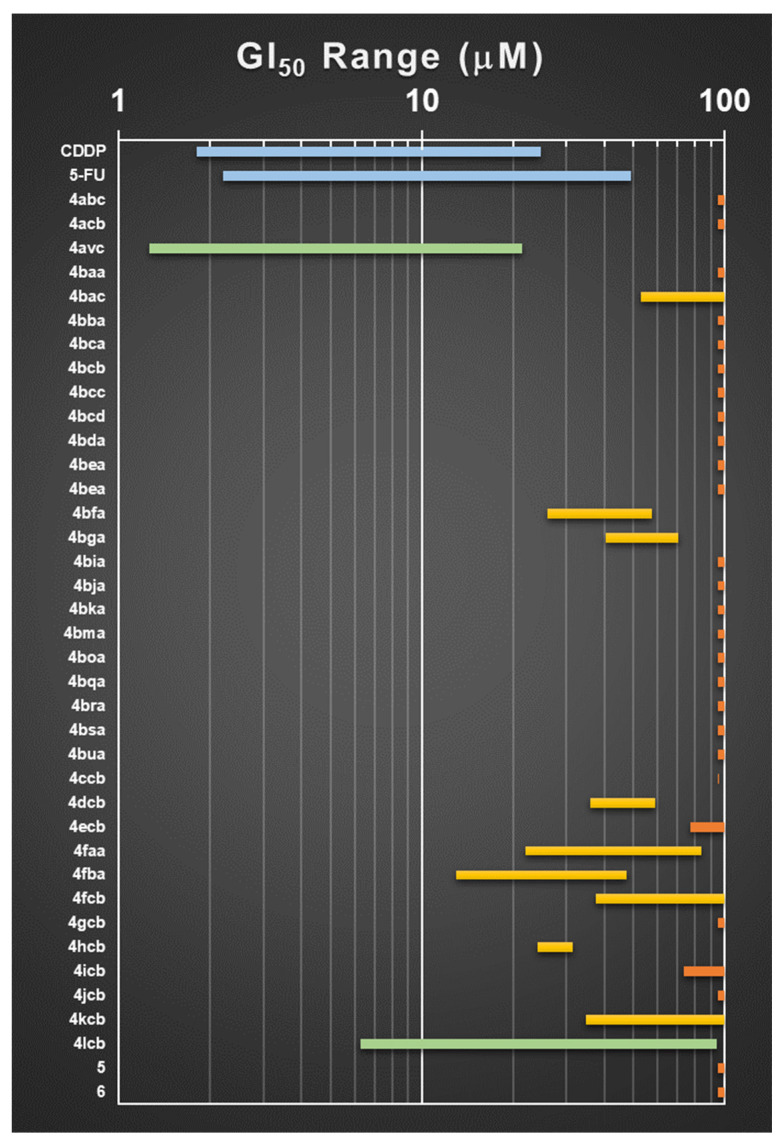
Range plot of GI_50_ values. Blue bars represent reference drugs cisplatin (CDDP) and 5-fluorouracil (5-FU). Green, yellow, and red bars denote active, intermediate, and poorly active compounds, respectively.

Next, we ran a preliminary screening to check the biological activity of the Passerini adducts. A panel of six representative human solid-tumor cell lines served as a model to test the antiproliferative activity of the compounds. The panel comprised lung (A549 and SW1573), cervix (HeLa), pancreas (MIA PaCa-2), breast (T-47D), and colon (WiDr) cancer cell lines. The test followed our implementation of the NCI protocol [38]. Thirty-eight samples underwent screening, whilst compounds **4bha**, **4bna**, **4bpa** and **4bta** were discarded due to poor solubility under the experimental conditions. The results (Figure 3) revealed that the majority of the compounds were inactive (GI_50_ > 100 μM against all cell lines). 

Table 3 shows the antiproliferative effects (50% growth inhibition, GI_50_) of active compounds. According to the data, compound **4avc** was the most potent compound of the series, with GI_50_ values in the range 1.3–21 μM. The α-bromo ester fragment is essential for the activity, since its modification led to an inactive derivative **5**. This result is consistent with a previous report on the antiproliferative effects of Ugi adducts of isatin [21]. When considering selectivity, compound **4avc** showed preferential activity against HeLa, MIA PaCa-2, SW1573, and T-47D cells. Interestingly, SW1573 and MIA PaCa-2 cells were more sensitive to **4avc** than A549 cells. A difference between these cell lines is the mutation in KRAS protein. The formers are KRAS G12C, whilst the latter is KRAS G12S. This difference was crucial for the different bioactivity profile of the Ugi adducts of isatin [39]. Interestingly, adduct **4lcb** also exhibited a preferential activity in SW1573 and MIA PaCa-2 cells. The Ugi adducts exhibited significantly greater differences in GI_50_ values across KRAS cell lines compared to the Passerini adducts. For example, the GI_50_ values for Ugi adducts varied from 1.3 μM in A549 cells to 2.3 nM in SW1573 cells and 3.8 nM in MIA PaCa-2 cells. In contrast, the Passerini adducts showed more moderate variation, with GI_50_ values of 21 μM in A549 cells, 1.3 μM in SW1573 cells, and 2.0 μM in MIA PaCa-2 cells (Table 3). Our results demonstrate that the presence of the α-halo ester on the isatin scaffold is key for the selectivity.

## 3. Materials and Methods

### 3.1. General Information 

All reagents were obtained from Sigma–Aldrich, Acros, BLDPharm, and TCI, and were used as received. Solvents were used as received. Reactions were conducted in microwave vials, round-bottom flasks, or in a Radley’s^®^ 12-position carousel reactor. Microwave reactions were conducted with a Biotage Initator+ Microwave System with an automated position system. Column chromatography was carried out on silica gel (Carlo Erba, 40–63 μm, 60Å). Thin-layer chromatography (TLC) was carried out on aluminum-backed Kieselgel 60 F254 plates (Merck and Macherey–Nagel). Plates were visualized either by UV light or with phosphomolybdic acid in ethanol. Melting points (m.p.) were determined with a Barnstead Electothermal 9100 apparatus and are uncorrected. NMR spectra were recorded with a Bruker Avance III instrument (400 MHz). Chemical shifts (δ) are given in parts per million (ppm) with respect to the solvent (CDCl_3_, 1H: δ = 7.26 ppm, 13C: δ = 77.2 ppm; DMSO-*d*6, 1H: δ = 2.50 ppm, 13C: δ = 39.5 ppm). Coupling constants (*J*) are reported in Hz and refer to apparent peak multiplicities. Splitting patterns are reported as s (singlet), d (doublet), t (triplet), q (quadruplet), m (multiplet), br (broad), dd (double of doublets), dt (double of triplets). Mass spectra (MS) were recorded with a quadrupole mass spectrometer Waters ZQ4000 (Chemistry department, University of Salamanca). The ionization was performed by ESI and the samples were infused in methanol. 

Isatin derivatives **1c** and **1d** were commercially available and used as received. The other isatin derivatives were synthesized using our previously reported procedures [40,41,42]. The characterization of compounds **4ccb**, **4bcb**, **4fcb**, **4bca**, **4bcd**, **4baa**, **4bda**, **4bea**, **4bka**, and **4bla** can be found in the literature [18,19,20]. 

### 3.2. The General Procedure for the Synthesis of α-Acyloxyamide-Oxindole Hybrids 4 Using the 3CPR

Microwave Reactor: In a microwave vial with a magnetic stirrer, the corresponding isatin derivative **1a–l** (0.4 mmol or 0.6 mmol), the carboxylic acid **2a–v**, the isocyanide **3a–d**, and the solvent (3 mL) were added. The vial was closed with a proper cap and the reaction mixture was left stirred at the indicated temperature for a certain time. The solvent was evaporated under reduced pressure, and the crude reaction mixture was purified in a short chromatographic glass column with SiO_2_ flash using hexane and AcOEt mixtures as the eluents.

Radley’s^®^ 12-position Carousel: In a glass reactor tube with a magnetic stirrer, the corresponding isatin derivative **1a–l** (1.0 mmol), the carboxylic acid **2a–v** (2.0 mmol), the isocyanide **3a–d** (2.0 mmol), powder 4Å MS (100 mg), and CH_3_CN (3 mL) were added. The vial was closed with a proper cap and the reaction mixture was left stirred at 100 °C for 24 h. The solvent was evaporated under reduced pressure, and the crude reaction mixture was purified in a short chromatographic glass column with SiO_2_ flash using hexane and AcOEt mixtures as the eluents.

*3-(Cyclohexylcarbamoyl)-2-oxoindolin-3-yl benzoate* **(4ccb)** [20]: **1c** (147.1 mg, 1.0 mmol), **2c** (159 mg, 2.0 mmol, 2 equivalents), **3b** (124 μL, 2.0 mmol, 2 equivalents), powder 4Å MS (100 mg), and CH_3_CN (3 mL) were used to obtain the corresponding **4ccb** as a white solid (69.4 mg, 16% yield), m.p. = 205.3–298.1 °C. ^1^H NMR (CDCl_3_, 400 MHz) δ: 0.83–0.90 (m, 1H, CH_2_), 1.28–1.42 (m, 4H, CH_2_), 1.60–1.64 (m, 1H, CH_2_), 1.71–1.76 (m, 2H, CH_2_), 1.95–2.03 (m, 2H, CH_2_), 3.81–3.89 (m, 1H, CH), 6.77 (d, 1H, NH), 6.87 (d, *J* = 8 Hz, 1H, Ar), 7.01 (t, *J* = 8 Hz, 1H, Ar), 7.24–7.32 (m, 2H, Ar), 7.47 (t, *J* = 8 Hz, 2H, Ar), 7.61 (t, *J* = 8 Hz, 1H, Ar), 7.99–8.02 (m, 2H, Ar), 8.48 (s br, 1H, NH). ^13^C APT NMR (CDCl_3_, 100 MHz) δ: 24.75, 24.79, 25.55, 32.65, 32.88, 49.06, 81.27, 110.93, 123.15, 124.39, 125.47, 128.54, 128.80, 130.08, 130.83, 134.09, 142.39, 163.37, 163.90, 172.80. HRMS (ESI) *m*/*z*: calculated for C_22_H_22_O_4_N_2_Na [M]^+^ 401.1472, found 401.1465.

*3-(Cyclohexylcarbamoyl)-5-methyl-2-oxoindolin-3-yl benzoate* **(4dcb)**: **1d** (100 mg, 0.62 mmol), **2c** (152 mg, 2.0 mmol, 2 equivalents), **3b** (149 μL, 2.0 mmol, 2 equivalents), powder 4Å MS (100 mg), and CH_3_CN (3 mL) were used to obtain the corresponding **4dcb** as a white solid (6 mg, <5% yield), m.p. = 139.5–142.1 °C. ^1^H NMR (CDCl_3_, 400 MHz) δ: 1.24–1.36 (m, 5H, CH_2_), 1.60–1.65 (m, 1H, CH_2_), 1.69–1.78 (m, 2H, CH_2_), 1.96–2.05 (m, 2H, CH_2_), 2.26 (s, 3H, CH_3_), 3.82–3.87 (m, 1H, CH), 6.74–6.80 (m, 2H, NH + Ar), 7.08 (d, *J* = 8 Hz, 1H, Ar), 7.13 (s, 1H, Ar), 7.46–7.49 (m, 2H, Ar), 7.62 (t, *J* = 8 Hz, 1H, Ar), 8.01 (d, *J* = 8 Hz, 2H, Ar), 8.09–8.12 (s br, 1H, NH). ^13^C APT NMR (CDCl_3_, 100 MHz) δ: 21.22, 24.76, 24.81, 25.57, 32.68, 32.91, 49.05, 81.33, 110.51, 125.16, 125.41, 128.61, 128.81, 130.10, 131.26, 132.82, 134.08, 139.74, 163.42, 163.89, 172.75. HRMS (ESI) *m*/*z*: calculated for C_23_H_24_O_4_N_2_Na [M]^+^ 415.1628, found 415.1621.

*5-Bromo-3-(cyclohexylcarbamoyl)-2-oxoindolin-3-yl benzoate* **(4hcb)**: **1h** (100 mg, 0.44 mmol), **2c** (108 mg, 2.0 mmol, 2 equivalents), **3b** (110 μL, 2.0 mmol, 2 equivalents), powder 4Å MS (100 mg), and CH_3_CN (3 mL) were used to obtain the corresponding **4hcb** as a white solid (46 mg, 33% yield), m.p. = 147.3–149.1 °C. ^1^H NMR (CDCl_3_, 400 MHz) δ: 1.32–1.46 (m, 5H, CH_2_), 1.62–1.65 (m, 1H, CH_2_), 1.72–1.78 (m, 2H, CH_2_), 1.95–2.00 (m, 2H, CH_2_), 3.80–3.87 (m, 1H, CH), 6.74 (d, *J* = 8 Hz, 1H, Ar), 6.80 (d, 1H, NH), 7.35–7.40 (m, 2H, Ar), 7.48 (t, *J* = 8 Hz, 2H, Ar), 7.63 (t, *J* = 8 Hz, 2H, Ar), 7.98–8.00 (m, 2H, Ar), 8.73 (s, 1H, NH). ^13^C APT NMR (CDCl_3_, 100 MHz) δ: 24.66, 24.71, 25.41, 32.53, 32.77, 49.18, 80.85, 112.42, 115.53, 127.22, 127.32, 128.81, 130.02, 133.56, 134.26, 141.47, 162.90, 163.75, 172.23. HRMS (ESI) *m*/*z*: calculated for C_22_H_21_O_4_N_2_BrNa [M]^+^ 479.0577, found 479.0568.

*3-(Cyclohexylcarbamoyl)-1-methyl-2-oxoindolin-3-yl benzoate* **(4bcb)** [19]: **1b** (100 mg, 0.62 mmol), **2c** (152 mg, 2.0 mmol, 2 equivalents), **3b** (149 μL, 2.0 mmol, 2 equivalents), powder 4Å MS (100 mg), and CH_3_CN (3 mL) were used to obtain the corresponding **4bcb** as a yellow solid (96 mg, 40% yield), m.p. = 145.7–149.2 °C.

*3-(Cyclohexylcarbamoyl)-1,5-dimethyl-2-oxoindolin-3-yl benzoate* **(4ecb)**: **1e** (100 mg, 0.57 mmol), **2c** (139 mg, 2.0 mmol, 2 equivalents), **3b** (136 μL, 2.0 mmol, 2 equivalents), powder 4Å MS (100 mg), and CH_3_CN (3 mL) were used to obtain the corresponding **4ecb** as a white solid (12 mg, <10% yield), m.p. = 178.7–181.2 °C. ^1^H NMR (CDCl_3_, 400 MHz) δ: 1.22–1.42 (m, 5H, CH_2_), 1.60–1.65 (m, 1H, CH_2_), 1.71–1.78 (m, 2H, CH_2_), 1.96–2.04 (m, 2H, CH_2_), 2.29 (s, 3H, CH_3_), 3.30 (s, 3H, CH_3_), 3.79–3.86 (m, 1H, CH), 6.78–6.82 (m, 2H, NH + Ar), 7.16–7.18 (m, 2H, Ar), 7.47 (t, *J* = 8 Hz, 2H, Ar), 7.61 (t, *J* = 8 Hz, 1H, Ar), 7.99 (d, *J* = 8 Hz, 2H, Ar). ^13^C APT NMR (CDCl_3_, 100 MHz) δ: 21.21, 24.75, 24.81, 25.59, 27.09, 32.68, 32.92, 49.03, 81.15, 108.66, 124.93, 125.12, 128.69, 128.78, 130.05, 131.18, 132.91, 134.01, 142.81, 163.45, 163.81, 171.48. HRMS (ESI) *m*/*z*: calculated for C_24_H_26_O_4_N_2_Na [M]^+^ 429.1785, found 429.1775.

*5-Bromo-3-(cyclohexylcarbamoyl)-1-methyl-2-oxoindolin-3-yl benzoate* **(4fcb)** [18]: **1f** (100 mg, 0.42 mmol), **2c** (102 mg, 2.0 mmol, 2 equivalents), **3b** (103 μL, 2.0 mmol, 2 equivalents), powder 4Å MS (100 mg), and CH_3_CN (3 mL) were used to obtain the corresponding 4fcb as a pale yellow solid (52 mg, 22% yield), m.p. = 190.1–192.3 °C. HRMS (ESI) *m*/*z*: calculated for C_23_H_23_O_4_N_2_BrNa [M]^+^ 493.07334, found 493.0725.

*1-Benzyl-3-(cyclohexylcarbamoyl)-2-oxoindolin-3-yl benzoate* **(4acb)**: **1a** (243 mg, 1.0 mmol), **2c** (244 mg, 2.0 mmol, 2 equivalents), 3b (250 μL, 2.0 mmol, 2 equivalents), powder 4Å MS (100 mg), and CH_3_CN (3 mL) were used to obtain the corresponding **4acb** as a pale yellow solid (384 mg, 86% yield), m.p. = 140.0–142.1 °C. ^1^H NMR (CDCl_3_, 400 MHz) δ: 1.28–1.40 (m, 5H, CH_2_), 1.62–1.66 (m, 1H, CH_2_), 1.74–1.79 (m, 2H, CH_2_), 2.02–2.04 (m, 2H, CH_2_), 3.84–3.93 (m, 1H, CH), 4.95 (d, *J* = 16 Hz, 1H, CH_2_), 5.14 (d, *J* = 16 Hz, 1H, CH_2_), 6.69 (d, *J* = 8 Hz, 1H, Ar), 6.79–6.81 (s br, 1H, NH), 7.01 (t, *J* = 8 Hz, 1H, Ar), 7.22 (t, *J* = 8 Hz, 1H, Ar), 7.26–7.38 (m, 4H, Ar), 7.44–7.51 (m, 4H, Ar), 7.63 (t, *J* = 8 Hz, 1H, Ar), 8.02 (d, *J* = 8 Hz, 2H, Ar). ^13^C APT NMR (CDCl_3_, 100 MHz) δ: 24.76, 24.78, 25.57, 32.76, 32.98, 44.47, 48.93, 81.25, 110.10, 123.24, 123.76, 127.15, 127.67, 128.81, 128.96, 130.06, 130.70, 134.07, 135.25, 144.26, 163.54, 163.64, 171.63. HRMS (ESI) *m*/*z*: calculated for C_29_H_28_O_4_N_2_Na [M]^+^ 491.1941, found 491.1931.

*5-Bromo-3-(cyclohexylcarbamoyl)-1-(3-methoxybenzyl)-2-oxoindolin-3-yl benzoate* **(4gcb)**: **1g** (100 mg, 0.28 mmol), **2c** (70 mg, 2.0 mmol, 2 equivalents), **3b** (72 μL, 2.0 mmol, 2 equivalents), powder 4Å MS (100 mg), and CH_3_CN (3 mL) were used to obtain the corresponding **4gcb** as a white solid (67 mg, 41% yield), m.p. = 154.6–157.3 °C. ^1^H NMR (CDCl_3_, 400 MHz) δ: 1.24–1.45 (m, 5H, CH_2_), 1.62–1.66 (m, 1H, CH_2_), 1.75–1.78 (m, 2H, CH_2_), 2.00–2.05 (m, 2H, CH_2_), 3.74 (s, 3H, OCH_3_), 3.83–3.91 (m, 1H, CH), 5.09 (s, 2H, CH_2_), 6.68–6.73 (m, 2H, Ar), 6.81 (d, 1H, NH), 7.05 (t, *J* = 8 Hz, 1H, Ar), 7.14 (d, *J* = 4 Hz, 1H, Ar), 7.27 (t, *J* = 8 Hz, 1H, Ar), 7.32 (d, *J* = 8 Hz, 1H, Ar), 7.44–7.51 (m, 3H, Ar), 7.64 (t, *J* = 8 Hz, 1H, Ar), 7.98–8.01 (m, 2H, Ar). ^13^C APT NMR (CDCl_3_, 100 MHz) δ: 24.71, 24.75, 25.52, 32.70, 32.92, 44.59, 48.93, 55.76, 81.40, 109.93, 112.51, 112.58, 116.39, 123.43, 125.26, 128.43, 128.86, 129.91, 130.77, 133.29, 134.17, 135.03, 143.89, 159.72, 163.48, 163.58, 171.51. HRMS (ESI) *m*/*z*: calculated for C_30_H_29_O_5_N_2_BrNa [M]^+^ 599.1152, found 599.1143.

*1-((1-Benzyl-1H-1,2,3-triazol-4-yl)methyl)-3-(cyclohexylcarbamoyl)-2-oxoindolin-3-yl benzoate* **(4lcb)**: **1l** (100 mg, 0.31 mmol), **2c** (76 mg, 2.0 mmol, 2 equivalents), **3b** (78 μL, 2.0 mmol, 2 equivalents), powder 4Å MS (100 mg), and CH_3_CN (3 mL) were used to obtain the corresponding **4lcb** as a pale yellow solid (90 mg, 52% yield), m.p. = 168.1–173.2 °C. ^1^H NMR (CDCl_3_, 400 MHz) δ: 1.27–1.42 (m, 5H, CH_2_), 1.61–1.65 (m, 1H, CH_2_), 1.73–1.76 (m, 2H, CH_2_), 1.95–2.01 (m, 2H, CH_2_), 3.79–3.86 (m, 1H, CH), 5.17 (s br, 2H, CH_2_), 5.45–5.53 (m, 2H, CH_2_), 6.75 (d, 1H, NH), 6.92–6.93 (m, 1H, Ar), 7.02 (t, *J* = 8 Hz, 1H, Ar), 7.21–7.33 (m, 8H, CH + Ar), 7.48 (t, *J* = 8 Hz, 2H, Ar), 7.63 (t, *J* = 8 Hz, 1H, Ar), 7.91–7.94 (m, 2H, Ar). ^13^C APT NMR (CDCl_3_, 100 MHz) δ: 24.76, 24.78, 25.54, 32.69, 32.91, 49.00, 81.17, 110.13, 123.44, 123.58, 125.14, 128.16, 128.44, 128.71, 128.82, 129.11, 129.99, 130.85, 134.18, 134.58, 143.56, 163.42, 163.65, 171.11. HRMS (ESI) *m*/*z*: calculated for C_32_H_31_O_4_N_5_Na [M]^+^ 572.2268, found 572.2256.

*1-Allyl-3-(cyclohexylcarbamoyl)-2-oxoindolin-3-yl benzoate* **(4icb)**: **1i** (100 mg, 0.53 mmol), **2c** (130 mg, 2.0 mmol, 2 equivalents), **3b** (132 μL, 2.0 mmol, 2 equivalents), powder 4Å MS (100 mg), and CH_3_CN (3 mL) were used to obtain the corresponding **4icb** as a white solid (57 mg, 26% yield), m.p. = 136.5–138.9 °C. ^1^H NMR (CDCl_3_, 400 MHz) δ: 1.21–1.43 (m, 5H, CH_2_), 1.60–1.64 (m, 1H, CH_2_), 1.72–1.77 (m, 2H, CH_2_), 1.96–2.04 (m, 2H, CH_2_), 3.80–3.89 (m, 1H, CH), 4.38–4.49 (m, 2H, CH_2_), 5.28 (d, *J* = 12 Hz, 1H, CH_2_), 5.46 (d, *J* = 20 Hz, 1H, CH_2_), 5.87–5.96 (m, 1H, CH), 6.77 (d, 1H, NH), 6.89 (d, *J* = 8 Hz, 1H, Ar), 7.04 (t, *J* = 8 Hz, 1H, Ar), 7.31–7.35 (m, 2H, Ar), 7.47 (t, *J* = 8 Hz, 2H, Ar), 7.61 (t, *J* = 8 Hz, 1H, Ar), 7.98–8.01 (m, 2H, Ar). ^13^C APT NMR (CDCl_3_, 100 MHz) δ: 24.72, 24.75, 25.53, 32.68, 32.91, 42.89, 48.92, 81.10, 109.84, 117.87, 123.14, 123.84, 125.22, 128.57, 128.76, 129.99, 130.66, 134.01, 144.34, 163.44, 163.60, 171.23. HRMS (ESI) *m*/*z*: calculated for C_25_H_26_O_4_N_2_Na [M]^+^ 441.1785, found 441.1776.

*(E)-1-(But-2-en-1-yl)-3-(cyclohexylcarbamoyl)-2-oxoindolin-3-yl benzoate* **(4jcb)**: **1j** (100 mg, 0.49 mmol), **2c** (121 mg, 2.0 mmol, 2 equivalents), **3b** (123 μL, 2.0 mmol, 2 equivalents), powder 4Å MS (100 mg), and CH_3_CN (3 mL) were used to obtain the corresponding **4jcb** as a white solid (105 mg, 50% yield), m.p. = 130.1–133.2 °C. ^1^H NMR (CDCl_3_, 400 MHz) δ: 1.24–1.43 (m, 5H, CH_2_), 1.60–1.64 (m, 1H, CH_2_), 1.71–1.77 (m, 5H, CH_3_ + CH_2_), 1.95–2.02 (m, 2H, CH_2_), 3.80–3.87 (m, 1H, CH), 4.31–4.43 (m, 2H, CH_2_), 5.50–5.57 (m, 1H, CH), 5.82–5.88 (m, 1H, CH), 6.76 (d, 1H, NH), 6.91 (d, *J* = 8 Hz, 1H, Ar), 7.04 (t, *J* = 8 Hz, 1H, Ar), 7.31–7.35 (m, 2H, Ar), 7.45–7.48 (m, 2H, Ar), 7.61 (t, *J* = 8 Hz, 1H, Ar), 7.99 (d, *J* = 8 Hz, 2H, Ar). ^13^C APT NMR (CDCl_3_, 100 MHz) δ: 17.91, 24.73, 24.76, 25.57, 32.69, 32.92, 42.46, 48.92, 81.07, 109.93, 123.06, 123.72, 124.03, 125.20, 128.66, 128.76, 129.34, 130.03, 130.66, 133.98, 144.52, 163.42, 163.67, 171.17. HRMS (ESI) *m*/*z*: calculated for C_26_H_28_O_4_N_2_Na [M]^+^ 455.1941, found 455.1934.

*3-(Cyclohexylcarbamoyl)-2-oxo-1-(prop-2-yn-1-yl)indolin-3-yl benzoate* **(4kcb)**: **1k** (100 mg, 0.54 mmol), **2c** (132 mg, 2.0 mmol, 2 equivalents), **3b** (134 μL, 2.0 mmol, 2 equivalents), powder 4Å MS (100 mg), and CH_3_CN (3 mL) were used to obtain the corresponding **4kcb** as a white solid (81 mg, 35% yield), m.p. = 145.7–147.2 °C. ^1^H NMR (CDCl_3_, 400 MHz) δ: 1.23–1.40 (m, 5H, CH_2_), 1.59–1.63 (m, 1H, CH_2_), 1.70–1.74 (m, 2H, CH_2_), 1.94–2.01 (m, 2H, CH_2_), 2.33–2.34 (m, 1H, CH), 3.80–3.87 (m, 1H, CH), 4.53–4.62 (m, 2H, CH_2_), 6.75 (d, 1H, NH), 7.08 (t, *J* = 8 Hz, 1H, Ar), 7.13 (d, *J* = 8 Hz, 1H, Ar), 7.35–7.47 (m, 4H, Ar), 7.57–7.61 (m, 1H, Ar), 7.99 (d, *J* = 8 Hz, 2H, Ar). ^13^C APT NMR (CDCl_3_, 100 MHz) δ: 24.59, 24.62, 25.39, 29.92, 32.49, 32.71, 48.87, 72.93, 76.11, 80.77, 109.94, 123.47, 124.03, 124.91, 128.26, 128.65, 129.91, 130.70, 133.97, 143.23, 162.95, 163.62, 170.54. HRMS (ESI) *m*/*z*: calculated for C_25_H_24_O_4_N_2_Na [M]^+^ 439.1628, found 439.1622.

*3-(tert-Butylcarbamoyl)-1-methyl-2-oxoindolin-3-yl benzoate* **(4bca)** [19]: **1b** (100 mg, 0.62 mmol), **2c** (152 mg, 1.2 mmol, 2 equivalents), **3a** (140 μL, 1.2 mmol, 2 equivalents), powder 4Å MS (100 mg), and CH_3_CN (3 mL) were used to obtain the corresponding **4bca** as a pale yellow solid (142 mg, 63% yield), m.p. = 144.3–146.7 °C.

*3-(Benzylcarbamoyl)-1-methyl-2-oxoindolin-3-yl benzoate* **(4bcc)**: **1b** (108.8 mg, 0.67 mmol), **2c** (152 mg, 1.2 mmol, 2 equivalents), **3c** (151 μL, 1.2 mmol, 2 equivalents), powder 4Å MS (100 mg), and CH_3_CN (3 mL) were used to obtain the corresponding **4bcc** as a white solid (177 mg, 66% yield), m.p. = 180.3–182.9 °C. ^1^H NMR (CDCl_3_, 400 MHz) δ: 3.33 (s, 3H, CH_3_), 4.52 (dd, *J* = 6 Hz, 2H, Ar), 4.64 (dd, *J* = 6 Hz, 2H, Ar), 6.94 (d, *J* = 8 Hz, 1H, Ar), 7.06–7.10 (m, 1H, Ar), 7.31–7.45 (m, 10H, Ar + NH), 7.57–7.61 (m, 1H, Ar), 7.95–7.98 (m, 2H, Ar). ^13^C APT NMR (CDCl_3_, 100 MHz) δ: 27.06, 43.93, 81.12, 109.03, 123.36, 124.32, 124.88, 127.66, 127.79, 128.39, 128.74, 128.93, 130.03, 131.00, 134.06, 137.57, 145.24, 163.79, 164.46, 171.36. HRMS (ESI) *m*/*z*: calculated for C_24_H_20_O_4_N_2_Na [M]^+^ 423.1315, found 423.1306.

*3-((2-Ethoxy-2-oxoethyl)carbamoyl)-1-methyl-2-oxoindolin-3-yl benzoate* **(4bcd)** [19]: **1b** (100 mg, 0.62 mmol), **2c** (152 mg, 1.2 mmol, 2 equivalents), **3d** (135 μL, 1.2 mmol, 2 equivalents), powder 4Å MS (100 mg), and CH_3_CN (3 mL) were used to obtain the corresponding **4bcd** as a white solid (57 mg, 23% yield), m.p. = 132.5–135.9 °C. 

*3-(tert-Butylcarbamoyl)-1-methyl-2-oxoindolin-3-yl 2-bromobenzoate* **(4baa)** [19]: **1b** (172 mg, 1.1 mmol), **2a** (402 mg, 2.0 mmol, 2 equivalents), **3a** (200 μL, 2.0 mmol, 2 equivalents), powder 4Å MS (100 mg), and CH_3_CN (3 mL) were used to obtain the corresponding **4baa** as a white solid (163 mg, 35% yield), m.p. = 158.9–160.1 °C.

*3-(tert-Butylcarbamoyl)-1-methyl-2-oxoindolin-3-yl 2-iodobenzoate* **(4bba)**: **1b** (170 mg, 1.1 mmol), **2b** (496 mg, 2.0 mmol, 2 equivalents), 3a (200 μL, 2.0 mmol, 2 equivalents), powder 4Å MS (100 mg), and CH_3_CN (3 mL) were used to obtain the corresponding **4bba** as a white solid (209 mg, 56% yield), m.p. = 192.4–196.3 °C. ^1^H NMR (CDCl_3_, 400 MHz) δ: 1.41 (s, 9H, 3H), 3.31 (s, 3H, CH_3_), 6.91 (d, *J* = 8 Hz, 1H, Ar), 7.01 (s br, 1H, NH), 7.09 (t, *J* = 8 Hz, 1H, Ar), 7.20 (t, *J* = 8 Hz, 1H, Ar), 7.35–7.44 (m, 3H, Ar), 7.74 (d, *J* = 8 Hz, 1H, Ar), 7.98 (d, *J* = 8 Hz, 1H, Ar). ^13^C APT NMR (CDCl_3_, 100 MHz) δ: 27.02, 28.76, 52.43, 82.12, 93.24, 109.01, 123.19, 124.01, 125.18, 128.38, 130.84, 132.39, 132.57, 133.44, 141.35, 145.55, 163.26, 163.73, 171.12. HRMS (ESI) *m*/*z*: calculated for C_21_H_21_O_4_N_2_INa [M]^+^ 515.0438, found 515.0433.

*3-(tert-Butylcarbamoyl)-1-methyl-2-oxoindolin-3-yl 2-chlorobenzoate* **(4bda)** [19]: **1b** (100 mg, 0.62 mmol), **2d** (307 mg, 1.2 mmol, 2 equivalents), **3a** (140 μL, 1.2 mmol, 2 equivalents), powder 4Å MS (100 mg), and CH_3_CN (3 mL) were used to obtain the corresponding **4bda** as a white solid (80 mg, 32% yield), m.p. = 149.1–152.4 °C. 

*3-(tert-Butylcarbamoyl)-1-methyl-2-oxoindolin-3-yl 2-fluorobenzoate* **(4bea)** [19]: **1b** (100 mg, 0.62 mmol), **2e** (174 mg, 1.2 mmol, 2 equivalents), **3a** (140 μL, 1.2 mmol, 2 equivalents), powder 4Å MS (100 mg), and CH_3_CN (3 mL) were used to obtain the corresponding 4bea as a white solid (92 mg, 39% yield), m.p. = 161.5–163.4 °C. 

*3-(tert-Butylcarbamoyl)-1-methyl-2-oxoindolin-3-yl 3-hydroxybenzoate* **(4bpa)**: **1b** (100 mg, 0.62 mmol), **2p** (171 mg, 1.2 mmol, 2 equivalents), **3a** (140 μL, 1.2 mmol, 2 equivalents), powder 4Å MS (100 mg), and CH_3_CN (3 mL) were used to obtain the corresponding **4bpa** as a white solid (196 mg, 83% yield), m.p. = 245.7–247.2 °C. ^1^H NMR (DMSO-*d*6, 400 MHz) δ: 1.34 (s, 9H, CH_3_), 3.18 (s, 3H, CH_3_), 7.02–7.12 (m, 3H, Ar), 7.36–7.40 (m, 3H, Ar), 7.46–7.52 (m, 2H, Ar), 7.62 (s br, 1H, NH), 10.03 (s br, 1H, OH). ^13^C APT NMR (DMSO-*d*6, 100 MHz) δ: 26.53, 28.28, 51.55, 81.25, 109.06, 116.25, 120.66, 121.39, 122.62, 122.92, 125.89, 129.18, 130.12, 130.44, 145.21, 157.61, 163.26, 164.08, 170.98. HRMS (ESI) *m*/*z*: calculated for C_21_H_22_O_5_N_2_Na [M]^+^ 405.1421, found 405.1413.

*3-(tert-Butylcarbamoyl)-1-methyl-2-oxoindolin-3-yl 3-bromobenzoate* **(4boa)**: **1b** (100 mg, 0.62 mmol), **2o** (250 mg, 1.24 mmol, 2 equivalents), **3a** 140 μL, 1.24 mmol, 2 equivalents), powder 4Å MS (100 mg), and CH_3_CN (3 mL) were used to obtain the corresponding **4boa** as a white solid (234 mg, 88% yield), m.p. = 201.7–204.7 °C. ^1^H NMR (CDCl_3_, 400 MHz) δ: 1.42 (s, 9H, CH_3_), 3.31 (s, 3H, CH_3_), 6.75 (s br, 1H, NH), 6.91 (d, *J* = 8 Hz, 1H, Ar), 7.08 (t, *J* = 8 Hz, 1H, Ar), 7.36–7.40 (m, 2H, Ar), 7.59–7.61 (m, 2H, Ar), 7.82–7.84 (m, 2H, Ar). ^13^C APT NMR (CDCl_3_, 100 MHz) δ: 27.05, 28.66, 52.28, 81.25, 108.95, 123.39, 124.49, 124.77, 127.49, 129.32, 130.93, 131.40, 132.17, 145.14, 162.87, 163.18, 171.58. HRMS (ESI) *m*/*z*: calculated for C_21_H_21_O_4_N_2_BrNa [M]^+^ 467.0577, found 467.0571.

*3-(tert-Butylcarbamoyl)-1-methyl-2-oxoindolin-3-yl 2-aminobenzoate* **(4bta)**: **1b** (100 mg, 0.62 mmol), **2t** (170 mg, 1.24 mmol, 2 equivalents), **3a** (140 μL, 1.24 mmol, 2 equivalents), powder 4Å MS (100 mg), and CH_3_CN (3 mL) were used to obtain the corresponding **4bta** as a pale yellow solid (78 mg, 33% yield), m.p. = 264.8–267.1 °C. ^1^H NMR (DMSO-*d*6, 400 MHz) δ: 1.32 (s, 9H, CH_3_), 3.18 (s, 3H, CH_3_), 6.53 (s br, 2H, NH_2_), 6.62 (t, *J* = 8 Hz, 1H, Ar), 6.76 (d, *J* = 8 Hz, 1H, Ar), 7.02–7.08 (m, 2H, Ar), 7.28–7.33 (m, 1H, Ar), 7.37 (t, *J* = 8 Hz, 1H, Ar), 7.49 (d, *J* = 8 Hz, 1H, Ar), 7.53 (s br, 1H, NH). ^13^C APT NMR (DMSO-*d*6, 100 MHz) δ: 26.52, 28.26, 51.49, 80.79, 106.76, 108.99, 114.94, 116.69, 122.52, 122.78, 126.27, 130.23, 131.37, 135.08, 145.10, 152.07, 164.30, 164.56, 171.29. HRMS (ESI) *m*/*z*: calculated for C_21_H_23_O_4_N_3_Na [M]^+^ 404.1581, found 404.1573.

*3-(tert-Butylcarbamoyl)-1-methyl-2-oxoindolin-3-yl 3-aminobenzoate* **(4bsa)**: **1b** (100 mg, 0.62 mmol), **2s** (170 mg, 1.24 mmol, 2 equivalents), **3a** (140 μL, 1.24 mmol, 2 equivalents), powder 4Å MS (100 mg), and CH_3_CN (3 mL) were used to obtain the corresponding **4bsa** as a pale yellow solid (105 mg, 44% yield), m.p. = 174.7–176.9 °C. ^1^H NMR (CDCl_3_, 400 MHz) δ: 1.42 (s, 9H, CH_3_), 3.30 (s, 3H, CH_3_), 6.78 (s br, 1H, NH), 6.87–6.91 (m, 2H, Ar), 7.05 (t, *J* = 8 Hz, 1H, Ar), 7.20–7.24 (m, 2H, Ar), 7.32–7.38 (m, 3H, Ar). ^13^C APT NMR (CDCl_3_, 100 MHz) δ: 27.03, 28.71, 52.22, 81.17, 108.88, 116.04, 119.79, 120.38, 123.27, 124.13, 125.22, 129.52, 129.65, 130.78, 145.28, 146.84, 163.39, 163.93, 171.73. HRMS (ESI) *m*/*z*: calculated for C_21_H_23_O_4_N_3_Na [M]^+^ 404.1581, found 404.1575.

*3-(tert-Butylcarbamoyl)-1-methyl-2-oxoindolin-3-yl 2,4-dibromobenzoate* **(4bna)**: **1b** (100 mg, 0.62 mmol), **2n** (347 mg, 1.24 mmol, 2 equivalents), **3a** (140 μL, 1.24 mmol, 2 equivalents powder 4Å MS (100 mg), and CH_3_CN (3 mL) were used to obtain the corresponding **4bna** as a white solid (290 mg, 89% yield), m.p. = 193.2–195.7 °C. ^1^H NMR (CDCl_3_, 400 MHz) δ: 1.43 (s, 9H, CH_3_), 3.31 (s, 3H, CH_3_), 6.76 (s br, 1H, NH), 6.92 (d, *J* = 8 Hz, 1H, Ar), 7.10 (t, *J* = 8 Hz, 1H, Ar), 7.38–7.41 (m, 1H, Ar), 7.87–7.88 (m, 1H, Ar), 8–02 (s, 2H, Ar). ^13^C APT NMR (CDCl_3_, 100 MHz) δ: 27.11, 28.65, 29.83, 52.44, 81.53, 109.06, 123.38, 123.60, 124.35, 124.88, 131.14, 131.72, 131.74, 131.86, 138.85, 139.25, 145.12, 161.56, 162.49, 171.36. HRMS (ESI) *m*/*z*: calculated for C_21_H_20_O_4_N_2_Br_2_Na [M]^+^ 544.9682, found 544.9672.

*3-(tert-Butylcarbamoyl)-1-methyl-2-oxoindolin-3-yl 4-aminobenzoate* **(4bra)**: **1b** (100 mg, 0.62 mmol), **2r** (170 mg, 1.24 mmol, 2 equivalents), **3a** (140 μL, 1.24 mmol, 2 equivalents), powder 4Å MS (100 mg), and CH_3_CN (3 mL) were used to obtain the corresponding **4bra** as a white solid (48 mg, 20% yield), m.p. = 169.3–170.1 °C. ^1^H NMR (CDCl_3_, 400 MHz) δ: 1.42 (s, 9H, CH_3_), 3.29 (s, 3H, CH_3_), 4.27 (s br, 2H, NH_2_), 6.58 (d, *J* = 8 Hz, 2H, Ar), 6.78 (s br, 1H, NH), 6.88 (d, *J* = 8 Hz, 1H, Ar), 7.04 (t, *J* = 8 Hz, 1H, Ar), 7.29 (d, *J* = 8 Hz, 1H, Ar), 7.35 (t, *J* = 8 Hz, 1H, Ar), 7.72 (d, *J* = 8 Hz, 2H, Ar). ^13^C APT NMR (CDCl_3_, 100 MHz) δ: 26.97, 28.69, 52.11, 80.90, 108.83, 113.84, 117.16, 123.12, 123.73, 125.77, 130.58, 132.12, 145.24, 152.11, 163.62, 163.84, 172.11. HRMS (ESI) *m*/*z*: calculated for C_21_H_23_O_4_N_3_Na [M]^+^ 404.1581, found 404.1574.

*3-(tert-Butylcarbamoyl)-1-methyl-2-oxoindolin-3-yl 4-fluoro-2-iodobenzoate* **(4bfa)**: **1b** (100 mg, 0.62 mmol), **2f** (330 mg, 1.24 mmol, 2 equivalents), **3a** (140 μL, 1.24 mmol, 2 equivalents), powder 4Å MS (100 mg), and CH_3_CN (3 mL) were used to obtain the corresponding **4bfa** as a pale yellow solid (278 mg, 88% yield), m.p. = 166.1–169.5 °C. ^1^H NMR (CDCl_3_, 400 MHz) δ: 1.41 (s, 9H, CH_3_), 3.32 (s, 3H, CH_3_), 6.91 (d, *J* = 8 Hz, 1H, Ar), 6.97 (s br, 1H, NH), 7.07–7.14 (m, 2H, Ar), 7.36–7.43 (m, 2H, Ar), 7.71 (dd, *J* = 8 Hz, 1H, Ar), 7.78–7.82 (m, 1H, Ar). ^13^C APT NMR (CDCl_3_, 100 MHz) δ: 27.08, 28.78, 52.51, 82.15, 93.85, 93.94, 109.09, 115.69, 115.90, 123.33, 124.24, 125.01, 128.65, 128.89, 130.94, 134.19, 134.28, 145.48, 162.66, 162.86, 163.06, 165.24, 171.22. HRMS (ESI) *m*/*z*: calculated for C_21_H_20_O_4_N_2_FINa [M]^+^ 533.0344, found 533.0334.

*3-(tert-Butylcarbamoyl)-1-methyl-2-oxoindolin-3-yl 2-bromo-4-(trifluoromethyl)benzoate* **(4bga)**: **1b** (100 mg, 0.62 mmol), **2g** (334 mg, 1.24 mmol, 2 equivalents), **3a** (140 μL, 1.24 mmol, 2 equivalents), powder 4Å MS (100 mg), and CH_3_CN (3 mL) were used to obtain the corresponding **4bga** as a pale yellow solid (197 mg, 61% yield), m.p. = 149.8–152.1 °C. ^1^H NMR (CDCl_3_, 400 MHz) δ: 1.40 (s, 9H, CH_3_), 3.32 (s, 3H, CH_3_), 6.93 (d, *J* = 8 Hz, 1H, Ar), 6.97 (s br, 1H, NH), 7.10 (t, *J* = 8 Hz, 1H, Ar), 7.35–7.40 (m, 2H, Ar), 7.65 (d, *J* = 8 Hz, 1H, Ar), 7.89 (d, *J* = 8 Hz, 1H, Ar), 7.94 (s, 1H, Ar). ^13^C APT NMR (CDCl_3_, 100 MHz) δ: 26.99, 28.60, 29.72, 52.40, 82.28, 109.08, 121.46, 123.33, 123.76, 124.56, 124.69, 131.04, 131.38, 132.17, 133.07, 145.46, 162.26, 162.89, 170.78. HRMS (ESI) *m*/*z*: calculated for C_22_H_20_O_4_N_2_BrF_3_Na [M]^+^ 535.0451, found 535.0443.

*3-(tert-Butylcarbamoyl)-1-methyl-2-oxoindolin-3-yl 3-hydroxy-4-methoxybenzoate* **(4bqa)**: **1b** (100 mg, 0.62 mmol), **2q** (208 mg, 1.24 mmol, 2 equivalents), **3a** (140 μL, 1.24 mmol, 2 equivalents), powder 4Å MS (100 mg), and CH_3_CN (3 mL) were used to obtain the corresponding **4bqa** as a pale yellow solid (196 mg, 83% yield), m.p. = 148.8–151.1 °C. ^1^H NMR (CDCl_3_, 400 MHz) δ: 1.44 (s, 9H, CH_3_), 3.30 (s, 3H, CH_3_), 3.92 (s, 3H, OCH_3_), 5.99 (s br, 1H, OH), 6.82–6.84 (m, 2H, NH + Ar), 6.89 (d, *J* = 8 Hz, 1H, Ar), 7.05 (t, *J* = 8 Hz, 1H, Ar), 7.31 (d, *J* = 8 Hz, 1H, Ar), 7.35 (t, *J* = 8 Hz, 1H, Ar), 7.46 (d, *J* = 4 Hz, 1H, Ar), 7.49–7.52 (m, 1H, Ar). ^13^C APT NMR (CDCl_3_, 100 MHz) δ: 27.03, 28.70, 52.22, 56.19, 81.21, 108.87, 110.16, 115.78, 121.45, 123.25, 123.51, 123.98, 125.42, 130.70, 145.25, 145.56, 151.36, 163.35, 163.49, 171.90. HRMS (ESI) *m*/*z*: calculated for C_22_H_24_O_6_N_2_Na [M]^+^ 435.1527, found 435.1519.

*3-(tert-Butylcarbamoyl)-1-methyl-2-oxoindolin-3-yl 2-phenylacetate* **(4bha)**: **1b** (100 mg, 0.62 mmol), **2h** (168 mg, 1.24 mmol, 2 equivalents), **3a** (140 μL, 1.24 mmol, 2 equivalents), powder 4Å MS (100 mg), and CH_3_CN (3 mL) were used to obtain the corresponding **4bha** as a white solid (36 mg, <5% yield), m.p. = 210.8–203.6 °C. ^1^H NMR (CDCl_3_, 400 MHz) δ: 1.27 (s, 9H, CH_3_), 3.23 (s, 3H, CH_3_), 3.73 (d, *J* = 4 Hz, 2H, CH_2_), 6.29 (s br, 1H, NH), 6.84 (d, *J* = 8 Hz, 1H, Ar), 7.02 (d, *J* = 8 Hz, 1H, Ar), 7.07–7.09 (m, 1H, Ar), 7.25–7.27 (m, 2H, Ar), 7.28–7.39 (m, 4H, Ar). ^13^C APT NMR (CDCl_3_, 100 MHz) δ: 26.91, 28.55, 41.10, 51.98, 81.10, 108.92, 123.06, 123.11, 125.28, 127.62, 128.99, 129.35, 130.76, 133.13, 145.39, 163.34, 168.01, 171.25. HRMS (ESI) *m*/*z*: calculated for C_22_H_24_O_4_N_2_Na [M]^+^ 403.1628, found 403.1619.

*3-(tert-Butylcarbamoyl)-1-methyl-2-oxoindolin-3-yl formate* **(4bia)**: **1b** (100 mg, 0.62 mmol), **2i** (46.8 µL, 1.24 mmol, 2 equivalents), **3a** (140 μL, 1.24 mmol, 2 equivalents), powder 4Å MS (100 mg), and CH_3_CN (3 mL) were used to obtain the corresponding **4bia** as a white solid (53 mg, 29% yield), m.p. = 163.4–165.7 °C. ^1^H NMR (CDCl_3_, 400 MHz) δ: 1.38 (s, 9H, CH_3_), 3.25 (s, 3H, CH_3_), 6.67 (s br, 1H, NH), 6.87 (d, *J* = 8 Hz, 1H, Ar), 7.07 (t, *J* = 8 Hz, 1H, Ar), 7.25–7.27 (m, 1H, Ar), 7.34–7.39 (m, 1H, Ar), 7.97 (s, 1H, Ar). ^13^C APT NMR (CDCl_3_, 100 MHz) δ: 26.97, 28.60, 52.30, 80.77, 109.05, 123.34, 123.61, 124.78, 131.01, 145.22, 157.20, 162.92, 170.79. HRMS (ESI) *m*/*z*: calculated for C_15_H_18_O_4_N_2_Na [M]^+^ 313.1159, found 313.1153.

*3-(tert-Butylcarbamoyl)-1-methyl-2-oxoindolin-3-yl tetradecanoate* **(4bja)**: **1b** (100 mg, 0.62 mmol), **2j** (283 mg, 1.24 mmol, 2 equivalents), **3a** (140 μL, 1.24 mmol, 2 equivalents), powder 4Å MS (100 mg), and CH_3_CN (3 mL) were used to obtain the corresponding **4bja** as a white solid (233 mg, 82% yield), m.p. = 136.3–138.8 °C. ^1^H NMR (CDCl_3_, 400 MHz) δ: 0.86–0.89 (m, 3H, CH_3_), 1.24–1.25 (m, 20H, CH_2_), 1.38 (s, 9H, CH_3_), 2.34–2.48 (m, 2H, CH_2_), 3.25 (s, 3H, CH_3_), 6.64 (s br, 1H, NH), 6.85 (d, *J* = 8 Hz, 1H, Ar), 7–05 (t, *J* = 8 Hz, 1H, Ar), 7.23 (d, *J* = 8 Hz, 1H, Ar), 7.34 (t, *J* = 8 Hz, 1H, Ar). ^13^C APT NMR (CDCl_3_, 100 MHz) δ: 14.25, 22.80, 24.72, 26.93, 28.63, 28.95, 29.32, 29.46, 29.50, 29.68, 29.75, 29.77, 32.03, 33.93, 52.10, 80.88, 108.89, 123.14, 123.38, 125.54, 130.65, 145.30, 163.42, 170.69, 171.71. HRMS (ESI) *m*/*z*: calculated for C_28_H_44_O_4_N_2_Na [M]^+^ 495.3193, found 495.3186.

*3-(tert-Butylcarbamoyl)-1-methyl-2-oxoindolin-3-yl picolinate* **(4bka)** [19]: **1b** (100 mg, 0.62 mmol), **2k** (153 mg, 1.24 mmol, 2 equivalents), **3a** (140 μL, 1.24 mmol, 2 equivalents), powder 4Å MS (100 mg), and CH_3_CN (3 mL) were used to obtain the corresponding **4bka** as a white solid (83 mg, 32% yield), m.p. = 161.5–163.0 °C. 

*3-(tert-Butylcarbamoyl)-1-methyl-2-oxoindolin-3-yl furan-2-carboxylate* **(4bla)** [19]: **1b** (100 mg, 0.62 mmol), **2l** (139 mg, 1.24 mmol, 2 equivalents), **3a** (140 μL, 1.24 mmol, 2 equivalents), powder 4Å MS (100 mg), and CH_3_CN (3 mL) were used to obtain the corresponding **4bla** as a pale yellow solid (154 mg, 70% yield), m.p. = 193.0–195.6 °C.

*3-(tert-Butylcarbamoyl)-1-methyl-2-oxoindolin-3-yl 5-nitrofuran-2-carboxylate* **(4bma)**: **1b** (100 mg, 0.62 mmol), **2m** (190 mg, 1.24 mmol, 2 equivalents), **3a** (140 μL, 1.24 mmol, 2 equivalents), powder 4Å MS (100 mg), and CH_3_CN (3 mL) were used to obtain the corresponding **4bma** as a pale yellow solid (133 mg, 58% yield), m.p. = 210.1–212.8 °C. ^1^H NMR (CDCl_3_, 400 MHz) δ: 1.44 (s, 9H, CH_3_), 3.29 (s, 3H, CH_3_), 6.86 (s br, 1H, NH), 6.92 (d, *J* = 8 Hz, 1H, Ar), 7.08 (t, *J* = 8 Hz, 1H, Ar), 7.29–7.34 (m, 3H, Ar), 7.40 (t, *J* = 8 Hz, 1H, Ar). ^13^C APT NMR (CDCl_3_, 100 MHz) δ: 27.05, 28.59, 52.42, 81.66, 109.18, 111.66, 120.65, 123.50, 123.83, 124.27, 131.39, 143.01, 145.50, 153.56, 162.58, 170.32. HRMS (ESI) *m*/*z*: calculated for C_19_H_19_O_7_N_3_Na [M]^+^ 424.1115, found 424.1104.

*1-Benzyl-3-(benzylcarbamoyl)-5-bromo-2-oxoindolin-3-yl 2-bromobenzoate* **(4mac)**: **1m** (171 mg, 0.5 mmol), **2a** (201 mg, 1.0 mmol, 2 equivalents), **3c** (123 μL, 1.0 mmol, 2 equivalents), powder 4Å MS (100 mg), and CH_3_CN (3 mL) were used to obtain the corresponding **4mac** as a pale yellow solid (229 mg, 67% yield), m.p. = 133.8–135.0 °C. ^1^H NMR (CDCl_3_, 400 MHz) δ: 4.52–4.64 (m, 2H, CH_2_), 4.96–5.12 (m, 2H, CH_2_), 6.57 (d, *J* = 8 Hz, 1H, Ar), 7.29–7.40 (m, 10H, Ar), 7.44–7.45 (m, 3H, Ar), 7.51–7.53 (m, 1H, Ar), 7.64–7.66 (m, 1H, Ar), 7.83–7.85 (m, 1H, Ar). ^13^C APT NMR (CDCl_3_, 100 MHz) δ: 44.18, 44.66, 81.82, 111.77, 115.89, 121.58, 126.77, 127.06, 127.20, 127.81, 127.88, 127.97, 128.03, 129.05, 130.11, 133.23, 133.68, 134.04, 134.69, 134.72, 137.13, 143.70, 163.05, 163.99, 170.44. HRMS (ESI) *m*/*z*: calculated for C_30_H_22_O_4_N_2_Br_2_Na [M]^+^ 654.9839, found 654.9830.

*3-(tert-Butylcarbamoyl)-1-methyl-2-oxoindolin-3-yl 1H-pyrrole-2-carboxylate* **(4bua)**: **1b** (100 mg, 0.62 mmol), **2u** (138 mg, 1.24 mmol, 2 equivalents), **3a** (140 μL, 1.24 mmol, 2 equivalents), powder 4Å MS (100 mg), and CH_3_CN (3 mL) were used to obtain the corresponding **4bua** as a white solid (196 mg, 83% yield), m.p. = 196.1–199.2 °C. ^1^H NMR (CDCl_3_, 400 MHz) δ: 1.41 (s, 9H, CH_3_), 3.25 (s, 3H, CH_3_), 6.24–6.26 (m, 1H, Ar), 6.77 (s br, 1H, NH), 6.85–6.87 (m, 2H, Ar), 6.95–6.96 (m, 1H, Ar), 7.02–7.06 (m, 1H, Ar), 7.28–7.37 (m, 2H, Ar), 9.49 (s br, 1H, NH). ^13^C APT NMR (CDCl_3_, 100 MHz) δ: 26.94, 28.64, 52,15, 80.88, 108.81, 110.81, 114.89, 116.71, 120.67, 123.18, 123.59, 123.79, 125.23, 125.57, 130.70, 145.74, 158.24, 163.47, 171.74. HRMS (ESI) *m*/*z*: calculated for C_19_H_21_O_4_N_3_Na [M]^+^ 378.1424, found 378.1417.

*1-Benzyl-3-(tert-butylcarbamoyl)-2-oxoindolin-3-yl 2-iodobenzoate* **(4aba)**: **1a** (102 mg, 0.4 mmol), **2b** (209 mg, 0.8 mmol, 2 equivalents), **3a** (95 μL, 0.8 mmol, 2 equivalents), powder 4Å MS (100 mg), and CH_3_CN (3 mL) were used to obtain the corresponding **4aba** as a pale yellow solid (74 mg, 30% yield), m.p. = 144.0–145.8 °C. ^1^H NMR (CDCl_3_, 400 MHz) δ: 1.43 (s, 9H, CH_3_), 5.05 (q, *J* = 16 Hz, 2H, CH_2_), 6.68 (d, *J* = 8 Hz, 1H, Ar), 7.02–7.06 (m, 2H, NH + Ar), 7.19–7.24 (m, 2H, Ar), 7.28 (d, *J*= 8 Hz, 1H, Ar), 7.33–7.37 (m, 2H, Ar), 7.42–7.47 (m, 4H, Ar), 7.80 (dd, *J* = 8 Hz, 1H, Ar), 8.00 (dd, *J* = 8 Hz, 1H, Ar). ^13^C APT NMR (CDCl_3_, 100 MHz) δ: 28.85, 44.51, 52.49, 82.35, 93.37, 110.21, 123.22, 123.89, 125.31, 127.27, 127.64, 128.43, 128.92, 130.69, 132.49, 133.62, 134.45, 135.27, 141.43, 144.61, 163.33, 163.73, 171.28. HRMS (ESI) *m*/*z*: calculated for C_27_H_25_O_4_N_2_INa [M]^+^ 591.0751, found 591.0743.

*1-Benzyl-3-(cyclohexylcarbamoyl)-2-oxoindolin-3-yl 2-iodobenzoate* **(4abb)**: **1a** (113 mg, 0.47 mmol), **2b** (209 mg, 0.8 mmol, 2 equivalents), **3b** (104 μL, 0.8 mmol, 2 equivalents), powder 4Å MS (100 mg), and CH_3_CN (3 mL) were used to obtain the corresponding **4abb** as a pale yellow solid (91 mg, 31% yield), m.p. = 155.2–157.4 °C. ^1^H NMR (CDCl_3_, 400 MHz) δ: 1.22–1.39 (m, 5H, CH_2_), 1.61–1.64 (m, 1H, CH_2_), 1.73–1.76 (m, 2H, CH_2_), 1.98–2.00 (m, 2H, CH_2_), 3.82–3.89 (m, 1H, CH), 4.96 (d, *J* = 16 Hz, 1H, CH_2_), 5.14 (d, *J* = 16 Hz, 1H, CH_2_), 6.67 (d, *J* = 8 Hz, 1H, Ar), 7.01–7.08 (m, 2H, NH + Ar), 7.20–7.28 (m, 3H, Ar), 7.33–7.37 (m, 2H, Ar), 7.40–7.48 (m, 4H, Ar), 7.80 (dd, *J* = 8 Hz, 1H, Ar), 8.01 (dd, *J* = 8 Hz, 1H, Ar). ^13^C APT NMR (CDCl_3_, 100 MHz) δ: 24.89, 24.91, 25.56, 32.94, 33.20, 44.50, 49.14, 82.25, 93.34, 110.22, 123.18, 123.76, 125.30, 127.20, 127.64, 128.45, 128.94, 130.75, 132.61, 133.66, 134.47, 135.25, 141.43, 144.61, 163.50, 163.72, 171.10. HRMS (ESI) *m*/*z*: calculated for C_29_H_27_O_4_N_2_INa [M]^+^ 617.0908, found 617.0895.

*1-Benzyl-3-(tert-butylcarbamoyl)-2-oxoindolin-3-yl 2-bromobenzoate* **(4aaa)**: **1a** (101 mg, 0.4 mmol), **2a** (169 mg, 0.8 mmol, 2 equivalents), **3a** (95 μL, 0.8 mmol, 2 equivalents), powder 4Å MS (100 mg), and CH_3_CN (3 mL) were used to obtain the corresponding **4aaa** as a pale yellow solid (115 mg, 52% yield), m.p. = 175.8–176.0 °C. ^1^H NMR (CDCl_3_, 400 MHz) δ: 1.43 (s, 9H, CH_3_), 5.05 (s, 2H, CH_2_), 6.67 (d, *J* = 8 Hz, 1H, Ar), 7.03 (t, *J* = 8 Hz, 1H, Ar), 7.13 (s br, 1H, NH), 7.21 (dt, *J* = 8 Hz, 1H, Ar), 7.28 (d, *J* = 8 Hz, 1H, Ar), 7.33–7.37 (m, 3H, Ar), 7.40–7.42 (m, 2H, Ar), 7.45–7.47 (m, 2H, Ar), 7.69–7.71 (m, 1H, Ar), 7.83–7.86 (m, 1H, Ar). ^13^C APT NMR (CDCl_3_, 100 MHz) δ: 28.82, 44.52, 52.40, 82.41, 110.23, 121.30, 123.23, 123.39, 125.45, 127.24, 127.63, 127.83, 128.93, 130.71, 130.78, 133.27, 133.83, 134.60, 135.29, 144.70, 163.16, 163.53, 171.21. HRMS (ESI) *m*/*z*: calculated for C_27_H_25_O_4_N_2_BrNa [M]^+^ 543.0889, found 543.0883.

*1-Benzyl-3-(benzylcarbamoyl)-2-oxoindolin-3-yl 2-iodobenzoate* **(4abc)**: **1a** (420 mg, 1.8 mmol), **2b** (892 mg, 3.6 mmol, 2 equivalents), **3c** (438 μL, 3.6 mmol, 2 equivalents), powder 4Å MS (200 mg), and CH_3_CN (6 mL) were used to obtain the corresponding **4abc** as a white solid (152 mg, 14% yield), m.p. = 142.8–145.3 °C. ^1^H NMR (CDCl_3_, 400 MHz) δ: 4.49–4.54 (m, 1H, CH_2_), 4.62–4.67 (m, 1H, CH_2_), 5.06 (q, *J* = 16 Hz, 2H, CH_2_), 6.71 (d, *J* = 8 Hz, 1H, Ar), 7.05 (t, *J* = 8 Hz, 1H, Ar), 7.18 (t, *J* = 8 Hz, 1H, Ar), 7.24–7.43 (m, 11H, Ar), 7.48 (d, *J* = 8 Hz, 2H, Ar), 7.77–7.80 (m, 1H, Ar), 7.95 (d, *J* = 8 Hz, 1H, Ar). ^13^C APT NMR (CDCl_3_, 100 MHz) δ: 44.06, 44.57, 93.46, 110.30, 123.29, 123.96, 125.03, 127.25, 127.69, 127.92, 128.06, 128.36, 128.96, 130.90, 132.47, 133.63, 134.18, 135.17, 137.37, 141.44, 144.61, 163.64, 164.45, 170.97. HRMS (ESI) *m*/*z*: calculated for C_30_H_23_O_4_N_2_INa [M]^+^ 625.0595, found 625.0584.

*1-Benzyl-3-(benzylcarbamoyl)-2-oxoindolin-3-yl 2-bromoacetate* **(4avc)**: **1a** (565 mg, 2.4 mmol), **2v** (667 mg, 4.8 mmol, 2 equivalents), **3c** (585 μL, 4.8 mmol, 2 equivalents), powder 4Å MS (200 mg), and CH_3_CN (6 mL) were used to obtain the corresponding **4avc** as a white solid (167 mg, 14% yield), m.p. = 196.7–197.2 °C. ^1^H NMR (CDCl_3_, 400 MHz) δ: 3.89 (q, *J* = 16 Hz, 2H, CH_2_), 4.47–4.62 (m, 2H, CH_2_), 5.00 (q, *J* = 16 Hz, 2H, CH_2_), 6.68 (d, *J* = 8 Hz, 1H, Ar), 7.05 (t, *J* = 8 Hz, 1H, Ar), 7.16 (s br, 1H, NH), 7.22–7.41 (m, 12H, Ar). ^13^C APT NMR (CDCl_3_, 100 MHz) δ: 24.84, 43.94, 44.55, 81.98, 110.38, 123.49, 123.53, 124.25, 127.16, 127.78, 127.93, 128.97, 129.00, 131.20, 134.93, 137.33, 144.48, 163.85, 164.08, 170.60. HRMS (ESI) *m*/*z*: calculated for C_25_H_21_O_4_N_2_BrNa [M]^+^ 515.0577, found 515.0571.

*3-(Benzylcarbamoyl)-1-methyl-2-oxoindolin-3-yl 2-bromobenzoate* **(4bac)**: **1b** (300 mg, 2.0 mmol), **2a** (804 mg, 4.0 mmol, 2 equivalents), **3c** (487 μL, 4.0 mmol, 2 equivalents), powder 4Å MS (200 mg), and CH_3_CN (6 mL) were used to obtain the corresponding **4bac** as a pale yellow solid (314 mg, 33% yield), m.p. = 142.8–145.3 °C. ^1^H NMR (CDCl_3_, 400 MHz) δ: 3.33 (s, 3H, CH_3_), 4.46–4.51 (m, 1H, CH_2_), 4.57–4.63 (m, 1H, CH_2_), 6.93 (d, *J* = 8 Hz, 1H, Ar), 7.09 (t, *J* = 8 Hz, 1H, Ar), 7.30–7.42 (m, 9H, Ar), 7.49 (s br, 1H, NH), 7.61–7.63 (m, 1H, Ar), 7.77–7.79 (m, 1H, Ar). ^13^C APT NMR (CDCl_3_, 100 MHz) δ: 27.09, 44.02, 82.12, 109.14, 121.42, 123.30, 123.79, 124.97, 127.70, 127.86, 127.92, 128.93, 130.47, 131.07, 132.94, 133.77, 134.57, 137.34, 145.61, 163.09, 164.44, 170.80. HRMS (ESI) *m*/*z*: calculated for C_24_H_19_O_4_N_2_BrNa [M]^+^ 501.0420, found 501.0410.

*5-Bromo-3-(tert-butylcarbamoyl)-1-methyl-2-oxoindolin-3-yl 2-bromobenzoate* **(4faa)**: **1f** (259 mg, 1.1 mmol), **2a** (402 mg, 2.0 mmol, 2 equivalents), **3a** (200 μL, 2.0 mmol, 2 equivalents), powder 4Å MS (100 mg), and CH_3_CN (3 mL) were used to obtain the corresponding **4faa** as a white solid (328 mg, 58% yield), m.p. = 195.9–198.1 °C. ^1^H NMR (CDCl_3_, 400 MHz) δ: 1.41 (s, 9H, CH_3_), 3.29 (s, 3H, CH_3_), 6.79 (d, *J* = 8 Hz, 1H, Ar), 7.09 (s br, 1H, NH), 7.39–7.43 (m, 3H, Ar), 7.50 (dd, *J* = 8 Hz, 1H, Ar), 7.69–7.71 (m, 1H, Ar), 7.79–7.82 (m, 1H, Ar). ^13^C APT NMR (CDCl_3_, 100 MHz) δ: 27.14, 28.72, 52.55, 81.62, 110.46, 115.74, 121.44, 126.69, 127.22, 127.86, 130.22, 133.23, 133.65, 134.04, 134.72, 144.72, 162.83, 163.11, 170.62. HRMS (ESI) *m*/*z*: calculated for C_21_H_20_O_4_N_2_Br_2_Na [M]^+^ 544.9682, found 544.9675.

*5-Bromo-3-(tert-butylcarbamoyl)-1-methyl-2-oxoindolin-3-yl 2-iodobenzoate* **(4fba)**: **1f** (268 mg, 1.1 mmol), **2b** (496 mg, 2.0 mmol, 2 equivalents), **3a** (200 μL, 2.0 mmol, 2 equivalents), powder 4Å MS (100 mg), and CH_3_CN (3 mL) were used to obtain the corresponding **4fba** as a white solid (311 mg, 48% yield), m.p. = 196.7–202.4 **°C**. ^1^H NMR (CDCl_3_, 400 MHz) δ: 1.41 (s, 9H, CH_3_), 3.29 (s, 3H, CH_3_), 6.79 (d, *J* = 8 Hz, 1H, Ar), 6.99 (s br, 1H, NH), 7.21 (dt, *J* = 8 Hz, 1H, Ar), 7.43 (t, *J* = 8 Hz, 1H, Ar), 7.48–7.52 (m, 2H, Ar), 7.74 (dd, *J* = 8 Hz, 1H, Ar), 7.98–8.01 (m, 1H, Ar). ^13^C APT NMR (CDCl_3_, 100 MHz) δ: 27.14, 28.75, 52.61, 81.54, 93.41, 110.45, 115.72, 127.06, 127.18, 128.44, 132.44, 133.60, 133.77, 133.99, 141.48, 144.63, 162.62, 163.69, 170.67. HRMS (ESI) *m*/*z*: calculated for C_21_H_20_O_4_N_2_BrINa [M]^+^ 592.9543, found 592.9536.

### 3.3. The Post-Passerini Reaction Transformations

*1,4′-Dibenzylspiro[indoline-3,2′-morpholine]-2,3′,6′-trione* **(5)**: The compound **4avc** (150 mg, 0.3 mmol) and CH_2_Cl_2_ (5 mL) were added to a round-bottom flask with a magnetic stirrer. DIPEA (58 µL, 0.34 mmol, 1.1 equivalents) was added, and the reaction was left stirring at room temperature for 24 h. The reaction mixture was quenched with H_2_O (10 mL) and extracted with CH_2_Cl_2_ (3 × 10 mL). After being dried with Na_2_SO_4_ and filtered, the solvent was evaporated under reduced pressure. The crude product was purified in a short chromatographic glass column with SiO_2_ flash and Hex:AcOEt (5:1) and (2:1) as eluents. The corresponding compound **5** was obtained as a white solid (8.3 mg, 7% yield), m.p. = 105.0–106.7 °C. ^1^H NMR (CDCl_3_, 400 MHz) δ: 4.65 (d, *J* = 16 Hz, 1H, CH_2_), 4.83–4.96 (m, 2H, CH_2_), 5.08 (s, 2H, CH_2_), 5.38 (d, *J* = 16 Hz, 1H, CH_2_), 6.72 (d, *J* = 8 Hz, 1H, Ar), 7.07 (t, *J* = 8 Hz, 1H, Ar), 7.19 (d, *J* = 8 Hz, 1H, Ar), 7.27–7.39 (m, 11H, Ar). ^13^C NMR (CDCl_3_, 100 MHz) δ: 42.95, 44.02, 64.11, 79.66, 110.45, 123.97, 124.88, 125.86, 127.10, 127.95, 128.05, 128.65, 128.78, 129.13, 131.57, 134.57, 136.06, 144.01, 167.34, 168.56, 170.89. HRMS (ESI) *m*/*z*: calculated for C_25_H_20_O_4_N_2_Na [M]^+^ 435.1315, found 435.1309.

*1-Methyl-3′H-spiro[indoline-3,1′-isobenzofuran]-2,3′-dione* **(6)**: In a Radley’s^®^ 12-position carousel reactor tube, CuI (3.6 mg, 0.019 mmol, 5 mol%), DMAP (9.3 mg, 0.076 mmol, 20 mol%), and DMSO (2 mL) were added, and the mixture was left stirring for 30 min at 30 °C. Then, compound **4bba** (184 mg, 0.38 mmol) and KOH (53 mg, 0.95 mmol, 2.5 equivalents) were added in the reaction tube and the reaction was left stirring at 120 °C for 5 h. The reaction mixture was quenched with H_2_O (10 mL) and extracted with AcOEt (3 × 10 mL). After being dried with Na_2_SO_4_ and filtered, the solvent was evaporated under reduced pressure. The crude product was purified in a short chromatographic glass column with SiO_2_ flash and Hex:AcOEt (5:1) and (2:1) as eluents. The corresponding compound **6** was obtained as a white solid (9.3 mg, 9% yield), m.p. = 174.7–176.9 °C. ^1^H NMR (CDCl_3_, 400 MHz) δ: 3.30 (s, 3H, CH_3_), 6.98–7.03 (m, 2H, Ar), 7.06–7.11 (m, 2H, Ar), 7.45 (t, *J* = 8 Hz, 1H, Ar), 7.58–7.64 (m, 2H, Ar), 8.00–8.03 (m, 1H, Ar). ^13^C NMR (CDCl_3_, 100 MHz) δ: 27.15, 84.57, 109.27, 121.97, 123.95, 124.95, 125.35, 126.08, 126.26, 130.48, 131.79, 134.91, 144.76, 146.95, 169.70, 171.32. HRMS (ESI) *m*/*z*: calculated for C_16_H_11_O_3_NNa [M]^+^ 288.0631, found 288.0626.

*5-Amino-N-(tert-butyl)-3-hydroxy-1-methyl-2-oxoindoline-3-carboxamide* **(7)**: In a Radley’s^®^ 12-position carousel reactor tube under nitrogen atmosphere, compound **4fba** (158 mg, 0.28 mmol), CuI (58 mg, 0.28 mmol, 1 equivalent), L-Proline (46 mg, 0.4 mmol, 1.3 equivalents), NaN_3_ (40 mg, 0.6 mmol, 2 equivalents), and DMSO (1 mL) were added, and the mixture was left stirring at 100 °C for 24 h. The reaction mixture was quenched with *brine* (10 mL) and extracted with AcOEt (3 × 10 mL). After being dried with Na_2_SO_4_ and filtered, the solvent was evaporated under reduced pressure. The crude product was purified in a short chromatographic glass column with SiO_2_ flash and Hex:AcOEt (1:1) and AcOEt as eluents. The corresponding compound **7** was obtained as a white solid (10 mg, 13% yield), m.p. = 220.0–222.4 °C. ^1^H NMR (DMSO-*d*6, 400 MHz) δ: 1.29 (s, 9H, CH_3_), 3.01 (s, 3H, CH_3_), 4.88 (s br, 2H, NH_2_), 6.51 (d, *J* = 8 Hz, 1H, Ar), 6.55 (s, 1H, Ar), 6.67 (d, *J* = 8 Hz, 1H, Ar), 7.02 (s, 1H, OH), 7.20 (s br, 1H, NH). ^13^C APT NMR (DMSO-*d*6, 100 MHz) δ: 26.01, 28.40, 50.38, 78.26, 108.94, 110.35, 113.74, 130.86, 134.32, 144.71, 168.10, 173.73. HRMS (ESI) *m*/*z*: calculated for C_14_H_19_O_3_N_3_Na [M]^+^ 300.1319, found 300.1314.

### 3.4. Antiproliferative Bioassays

The antiproliferative tests were conducted using our implementation of the NCI protocol [38]. The cell-seeding densities were 2500 (A549, HeLa, MIA PaCa-2, and SW1573) or 5000 (T-47D and WiDr) cells/well. Stock solutions of isatin-based Passerini adducts were prepared in DMSO at 40 mM. The maximum test concentration was 100 μM. The exposure time was set at 48 h. At the end of the exposure, the SRB protocol was applied. The results were expressed as 50% growth inhibition (GI_50_).

## 4. Conclusions

A library of 44 α-acyloxyamide–oxindole hybrids was obtained efficiently using the valuable 3CPR approach. The reaction was optimized in terms of temperature, time, solvent, and the presence of additives, using a microwave or Radley’s carousel reactors. The component scope was also evaluated, proving that the 3CPR has a great tolerance towards isatin, carboxylic acid, and isocyanide derivatives. In general, moderate yields of the desired α-acyloxyamide–oxindole hybrids were obtained in 24 h, using CH_3_CN as the solvent. The success of the gram-scale reaction was a clear demonstration of the competence of the synthetic method. Post-Passerini reactions also supported the versatility of the α-acyloxyamide–oxindole hybrids’ scaffold in accessing other interesting derivatives. Most of the library of the α-acyloxyamide–oxindole hybrids was evaluated regarding their antiproliferative activity in six human solid-tumor cell lines, with **4avc** being the most potent compound of the series, with GI_50_ values in the range 1.3–21 μM. Further studies on the mode of action and lead discovery are in progress and will be reported shortly.

## Figures and Tables

**Table 1:** Survey of the 3CPR using microwave reaction conditions.

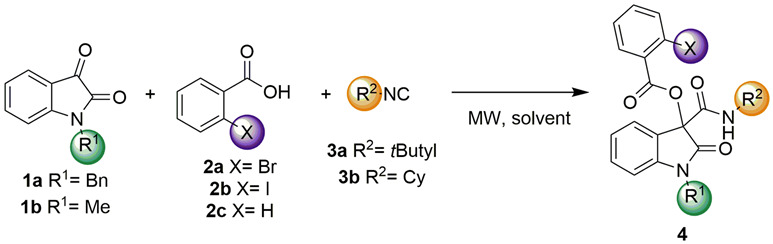										
Entry ^a^	1	2	Equiv.	3	Equiv.	Solvent	Temp./°C	T/h	4	Yield ^c^/%
1	**1a**	**2b**	1.0	**3a**	2.0	Toluene	60	0.25	**4aba**	0
2	**1a**	**2b**	1.5	**3a**	2.0	Toluene	120	0.5	**4aba**	60
3	**1a**	**2b**	2.0	**3a**	1.5	Toluene	120	3	**4aba**	91
4	**1a**	**2b**	3.0	**3a**	2.4	Toluene	120	3	**4aba**	59
5	**1a**	**2b**	2.0	**3a**	1.5	1,4-Dioxane	120	3	**4aba**	93
6	**1a**	**2a**	2.0	**3a**	1.5	Toluene	120	3	**4aaa**	85
7	**1a**	**2a**	3.0	**3a**	2.4	Toluene	120	3	**4aaa**	72
8	**1a**	**2b**	2.0	**3b**	1.5	Toluene	120	3	**4abb**	76
9	**1a**	**2b**	2.0	**3b**	1.5	Toluene	120	1	**4abb**	57
10	**1a**	**2b**	1.5	**3b**	1.2	Toluene	120	1	**4abb**	49
11	**1a**	**2b**	2.0	**3b**	1.5	DMF	120	1	**4abb**	11
12	**1a**	**2b**	2.0	**3b**	1.5	1,4-Dioxane	120	1	**4abb**	50
13	**1a**	**2c**	2.0	**3b**	1.5	Toluene	120	3	**4acb**	86
14	**1b**	**2c**	2.0	**3b**	1.5	Toluene	120	3	**4bcb**	89
15 ^b^	**1b**	**2c**	1.3	**3b**	1.0	CH_3_CN	80	2	**4bcb**	35
16 ^b^	**1b**	**2c**	2.5	**3b**	2.0	CH_3_CN	80	2	**4bcb**	50
17 ^b^	**1b**	**2c**	4.0	**3b**	2.0	CH_3_CN	80	2	**4bcb**	33

^a^ Reaction conditions: **1a** (0.4 mmol) or **1b** (0.6 mmol), **2**, **3**, and the solvent (3 mL) were added to a microwave vial and stirred at the indicated temperature for a certain time. ^b^ 4Å MS (100 mg) were added to the reaction vial. ^c^ Isolated yield.

**Table 2:** Survey of the 3CPR using batch reaction conditions with a Radley’s^®^ 12-position carousel system.

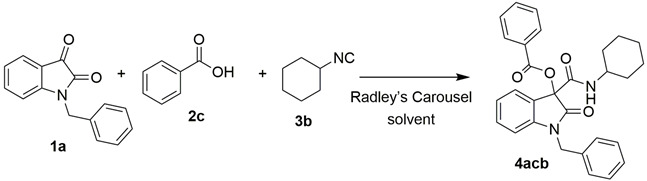							
Entry ^a^	(2a) Equiv.	(3b) Equiv.	Solvent	Additive	Temp./°C	t/h	Yield ^d^/%
1	2.0	1.5	CH_3_CN	4Å MS ^b^	80	24	34
2	1.3	1.0	CH_3_CN	4Å MS ^b^	80	16	33
3	2.0	2.0	CH_3_CN	4Å MS ^b^	80	87	73
4	2.0	2.0	CH_3_CN	4Å MS ^b,c^	80	15	30
5	2.0	2.0	CH_3_CN	4Å MS ^b^	100	24	86
6	2.0	2.0	CH_3_CN	-	100	24	69
7	2.0	2.0	CH_3_CN	5Å MS	100	24	35
8	2.0	2.0	CH_3_CN	4Å MS	100	24	83
9	2.0	2.0	MeOH	4Å MS ^b^	100	24	21
10	2.0	2.0	EtOH	4Å MS ^b^	100	24	21
11	2.0	2.0	2-MeTHF	4Å MS ^b^	100	24	74
12	2.0	2.0	TFE	4Å MS ^b^	100	24	70
13	2.0	2.0	CH_2_Cl_2_	4Å MS ^b^	60	24	54
14	2.5	2.5	CH_3_CN	4Å MS ^b^	100	24	55

^a^ Reaction conditions: **1a** (1.0 mmol), **2a**, **3b**, MS (100 mg) and solvent (3 mL) were added to a Radley’s^®^ 12-position carousel reactor tube and stirred at the indicated temperature for a certain time. ^b^ Powder. ^c^ ZnF_2_ (10 mol%) was added to the reaction media. ^d^ Isolated yield. TFE: 2,2,2-trifluoroethanol.

**Table 3:** Antiproliferative activity (GI_50_, μM) of Passerini adducts **4** active against human solid-tumor cell lines ^a^.

Compound	A549	HeLa	MIA PaCa-2	SW1573	T-47D	WiDr
**4avc**	21 ± 2.9	4.3 ± 1.6	2.0 ± 0.4	1.3 ± 0.2	6.3 ± 1.6	20 ± 5.9
**4bac**	>100	87 ± 19	>100	>100	>100	53 ± 23
**4bfa**	44 ± 17	36 ± 9.3	58 ± 12	31 ± 7.0	26 ± 5.0	43 ± 16
**4bga**	70 ± 28	41 ± 7.9	45 ± 8.9	59 ± 16	44 ± 13	41 ± 14
**4dcb**	59 ± 13	36 ± 4.5	36 ± 4.4	49 ± 12	40 ± 3.2	42 ± 1.4
**4faa**	78 ± 7.4	31 ± 8.2	84 ± 23	75 ± 26	22 ± 3.8	31 ± 6.8
**4fba**	48 ± 4.0	40 ± 2.7	37 ± 13	43 ± 18	13 ± 0.4	24 ± 9.7
**4fcb**	>100	43 ± 8.4	78 ± 38	38 ± 8.8	41 ± 16	60 ± 20
**4hcb**	32 ± 1.0	24 ± 6.8	27 ± 7.3	31 ± 4.9	25 ± 10	29 ± 6.6
**4kcb**	79 ± 36	37 ± 0.9	>100	>100	>100	35 ± 6.7
**4lcb**	15 ± 1.6	26 ± 8.5	6.3 ± 2.1	7.1 ± 2.4	94 ± 11	70 ± 27

^a^ Values represent mean ± standard deviation of three independent experiments.

## Data Availability

Data are contained within the article and Supplementary Materials.

## Supplementary Materials

The following supporting information can be downloaded at: https://www.mdpi.com/article/10.3390/molecules29235538/s1, ^1^H and ^13^C NMR spectra.

## References

[1] Buskes M.J., Coffin A., Troast D.M., Stein R., Blanco M.-J. (2023). Accelerating Drug Discovery: Synthesis of Complex Chemotypes via Multicomponent Reactions. ACS Med. Chem. Lett..

[2] Cores Á., Clerigué J., Orocio-Rodríguez E., Menéndez J.C. (2022). Multicomponent Reactions for the Synthesis of Active Pharmaceutical Ingredients. Pharmaceuticals.

[3] Insuasty D., Castillo J., Becerra D., Rojas H., Abonia R. (2020). Synthesis of Biologically Active Molecules through Multicomponent Reactions. Molecules.

[4] Younus H.A., Al-Rashida M., Hameed A., Uroos M., Salar U., Rana S., Khan K.M. (2020). Multicomponent reactions (MCR) in medicinal chemistry: A patent review (2010–2020). Expert Opin. Ther. Pat..

[5] Totobenazara J., Bacalhau P., San Juan A.A., Marques C.S., Fernandes L., Goth A., Caldeira A.T., Martins R., Burke A.J. (2016). Design, Synthesis and Bioassays of 3-Substituted-3-Hydroxyoxindoles for Cholinesterase Inhibition. Chem. Sel..

[6] Marques C.S., López Ó., Bagetta D., Carreiro E.P., Petralla S., Bartolini M., Hoffmann M., Alcaro S., Monti B., Bolognesi M.L. (2020). *N*-1,2,3-triazole-isatin derivatives for cholinesterase and β-amyloid aggregation inhibition: A comprehensive bioassay study. Bioorg. Chem..

[7] Marques C.S., López Ó., Leitzbach L., Fernández-Bolaños J.G., Stark H., Burke A.J. (2022). Survey of New Small Molecule Isatin-based Oxindole Hybrids as Multi-Targeted Drugs for the Treatment of Alzheimer’s Disease. Synthesis.

[8] Hofmanova T., Marques C., García-Sosa A.-T., López Ó., Leitzbach L., Carreiro E.P., González-Bakker A., Puerta A., Stark H., Padrón J.M. (2023). *N*-Substituted 3-Aminooxindoles and *N*-Propargyl Derivatives: Potential biological activities against Alzheimer’s disease. Results Chem..

[9] Busto N., Leitão-Castro J., García-Sosa A.-T., Cadete F., Marques C.S., Freitas R., Burke A.J. (2022). *N*-1,2,3-Triazole-Isatin Derivatives: Anti-proliferation effects and target identification in solid tumour cell lines. RSC Med. Chem..

[10] Marques C.S., González-Bakker A., Padrón J.M., Burke A.J. (2023). Easy access to Ugi-derived Isatin-peptoids and their potential as small-molecule anticancer agents. New J. Chem..

[11] Marques C.S., González-Bakker A., Padrón J.M. (2024). The Ugi4CR as effective tool to access promising anticancer isatin-based α-acetoamide carboxamide oxindole hybrids. Beilstein J. Org. Chem..

[12] Brandão P., Marques C.S., Burke A.J., Pineiro M. (2021). The application of isatin-based multicomponent-reactions in the quest for new bioactive and druglike molecules. Eur. J. Med. Chem..

[13] Brandão P., Marques C.S., Carreiro E.P., Pineiro M., Burke A.J. (2021). Engaging isatins in multicomponent reactions (MCRs)—Easy access to structural diversity. Chem. Rec..

[14] Passerini M. (1921). Isonitriles. II. Compounds with aldehydes or with ketones and monobasic organic acids. Gazz. Chim. Ital..

[15] Banfi L., Basso A., Lambruschini C., Moni L., Riva R. (2021). The 100 facets of the Passerini reaction. Chem. Sci..

[16] Dömling A. (2023). Innovations and Inventions: Why Was the Ugi Reaction Discovered Only 37 Years after the Passerini Reaction?. J. Org. Chem..

[17] Wahby Y., Abdel-Hamid H., Ayoup M.S. (2022). Two decades of recent advances of Passerini reactions: Synthetic and potential pharmaceutical applications. New J. Chem..

[18] Esmaeili A.A., Ghalandarabad S.A., Jannati S. (2013). A novel and efficient synthesis of 3,3-disubstituted indol-2-ones via Passerini three-component reactions in the presence of 4 Å molecular sieves. Tetrahedron Lett..

[19] Kaicharla T., Yetra S.R., Roy T., Biju A.T. (2013). Engaging isatins in solvent-free, sterically congested Passerini reaction. Green Chem..

[20] Shaabani A., Afshari R., Hooshmand S.E. (2016). Passerini three-component cascade reactions in deep eutectic solvent: An environmentally benign and rapid system for the synthesis of α-acyloxyamides. Res. Chem. Intermed..

[21] Brandão P., Puerta A., Padrón J.M., Kuznetsov M.L., Burke A.J., Pineiro M. (2021). Ugi Adducts of Isatin as Promising Antiproliferative Agents with Druglike Properties. Asian J. Org. Chem..

[22] Salami S.A., Safari J.B., Smith V.J., Krause R.W.M. (2023). Mechanochemically-Assisted Passerini Reactions: A Practical and Convenient Method for the Synthesis of Novel α-Acyloxycarboxamide Derivatives. ChemistryOpen.

[23] Salami S.A., Smith V.J., Krause R.W.M. (2023). Water-Assisted Passerini Reactions under Mechanochemical Activation: A Simple and Straightforward Access to Oxindole Derivatives. ChemistrySelect.

[24] Dhokne P., Sakla A.P., Shankaraiah N. (2021). Structural insights of oxindole based kinase inhibitors as anticancer agents: Recent advances. Eur. J. Med. Chem..

[25] Yu B., Yu D.-Q., Liu H.-M. (2015). Spirooxindoles: Promising scaffolds for anticancer agents. Eur. J. Med. Chem..

[26] Iqbal N., Iqbal N. (2014). Imatinib: A Breakthrough of Targeted Therapy in Cancer. Chemother. Res. Pract..

[27] Barreto A.F.S., Vercillo O.E., Andrade C.K.Z. (2011). Microwave-Assisted Passerini Reactions under Solvent-Free Conditions. J. Braz. Chem. Soc..

[28] Byrne F.P., Jin S., Paggiola G., Petchey T.H.M., Clark J.H., Farmer T.J., Hunt A.J., McElroy C.R., Sherwood J. (2016). Tools and techniques for solvent selection: Green solvent selection guides. Sustain. Chem Process.

[29] McConvey I.F., Woods D., Lewis M., Gan Q., Nancarrow P. (2012). The Importance of Acetonitrile in the Pharmaceutical Industry and Opportunities for its Recovery from Waste. Org. Process Res. Dev..

[30] Singh G.S., Desta Z.Y. (2012). Isatins As Privileged Molecules in Design and Synthesis of Spiro- Fused Cyclic Frameworks. Chem. Rev..

[31] Buskes M.J., Blanco M.-J. (2020). Impact of Cross-Coupling Reactions in Drug Discovery and Development. Molecules.

[32] Dorel R., Grugel C.P., Haydl A.M. (2019). The Buchwald–Hartwig Amination After 25 Years. Angew. Chem. Int. Ed..

[33] Heravi M.M., Kheilkordi Z., Zadsirjan V., Heydari M., Malmir M. (2018). Buchwald-Hartwig reaction: An overview. J. Organomet. Chem..

[34] Lin H., Sun D., Lin H., Sun D. (2013). Recent Synthetic Developments and Applications of the Ullmann Reaction. A Review. Org. Prep. Proced. Int..

[35] Monnier F., Taillefer M. (2008). Catalytic C-C, C-N, and C-O Ullmann-Type Coupling Reactions: Copper Makes a Difference. Angew. Chem. Int. Ed..

[36] Roy S., Dutta M.M., Sarma M.J., Phukan P. (2019). Accelerating Effect of DMAP on CuI Catalyzed Buchwald- Hartwig C-N Coupling: Mechanistic Insight to the Reaction Pathway. ChemistrySelect.

[37] Brunelli F., Ceresa C., Fracchia L., Tron G.C., Aprile S. (2022). Expanding the Chemical Space of Drug-like Passerini Compounds: Can α-Acyloxy Carboxamides Be Considered Hard Drugs?. ACS Med. Chem. Lett..

[38] Puerta A., Galán A.R., Abdilla R., Demanuele K., Fernandes M.X., Bosica G., Padrón J.M. (2019). Naphthol-derived Betti bases as potential SLC6A14 blockers. J. Mol. Clin. Med..

[39] Puerta A., González-Bakker A., Brandão P., Pineiro M., Burke A.J., Giovannetti E., Fernandes M.X., Padrón J.M. (2024). Early pharmacological profiling of isatin derivatives as potent and selective cytotoxic agents. Biochem. Pharmacol..

[40] Marques C.S., McArdle P., Erxleben A., Burke A.J. (2020). Accessing New 5-α-(3,3-Disubstituted Oxindole)-Benzylamine Derivatives from Isatin: Stereoselective Organocatalytic Three Component Petasis Reaction. Eur. J. Org. Chem..

[41] Marques C.S., Burke A.J. (2016). Enantioselective Rhodium(I)-Catalyzed Additions of Arylboronic Acids to *N*-1,2,3-Triazole-Isatin Derivatives: Accessing *N*-(1,2,3-Triazolmethyl)-3-hydroxy-3-aryloxindoles. ChemCatChem.

[42] Marques C.S., Burke A.J. (2016). Modular Catalytic Synthesis of 3-Amino-3-aryl-2-oxindoles: Rh Catalysis with Isatin-Derived N-Boc-Protected Ketimines. Eur. J. Org. Chem..

