# Supplementary material for: Accessing Promising Passerini Adducts in Anticancer Drug Design
Source: Molecules. 2024 Nov 23;29(23):5538. doi: 10.3390/molecules29235538 (PMC11643473; doi:10.3390/molecules29235538)

Accessing Promising Passerini-Adducts in Anticancer Drug Design

Ana Margarida Janeiro[1], Aday González-Bakker[2], José M. Padrón[2] and Carolina S. Marques[3,*]

[1]Faculty of Pharmacy, University of Lisbon, Av. Prof. Gama Pinto, 1649-003 Lisbon, Portugal;
[2]BioLab, Instituto Universitario de Bio-Orgánica Antonio González (IUBO-AG), Universidad de La Laguna, PO Box 456, 38200, La Laguna, Spain;
[3]LAQV-REQUIMTE, University of Évora, Institute for Research and Advanced Studies, Rua Romão Ramal-ho, 59, 7000-641, Évora, Portugal;
*Correspondence: carolsmarq@uevora.pt

^{1}H and ^{13}C NMR spectra

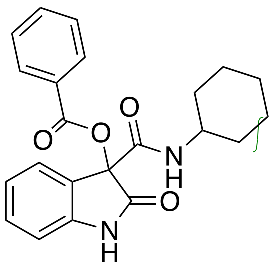

(4ccb)

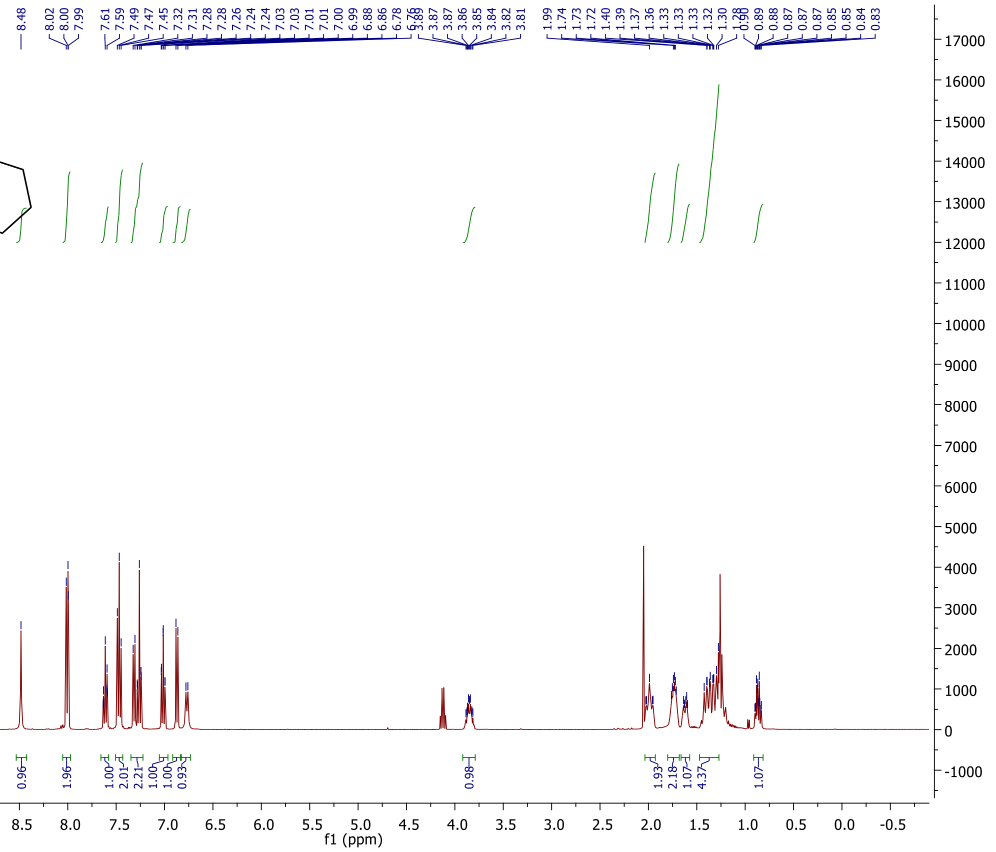

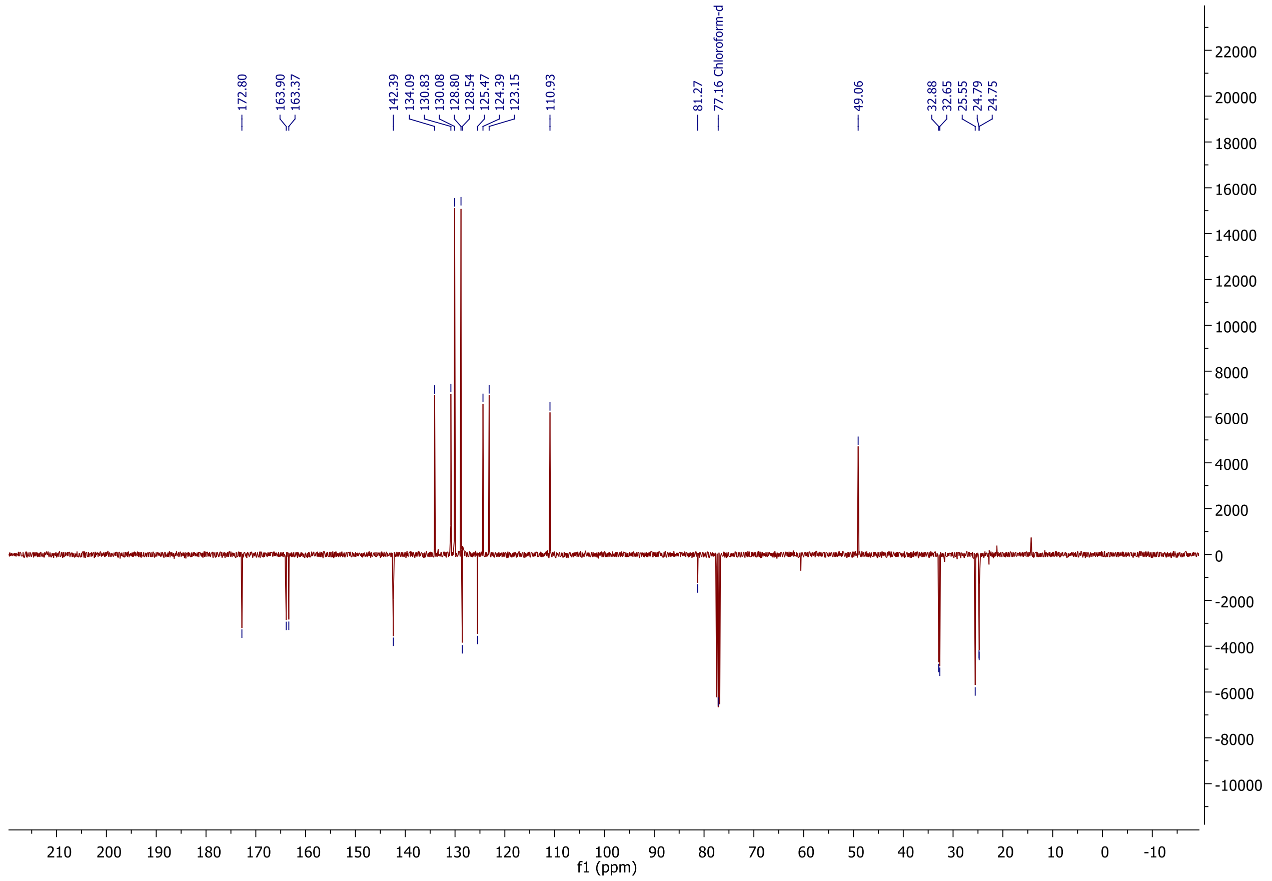

^{1}H and ^{13}C NMR spectra

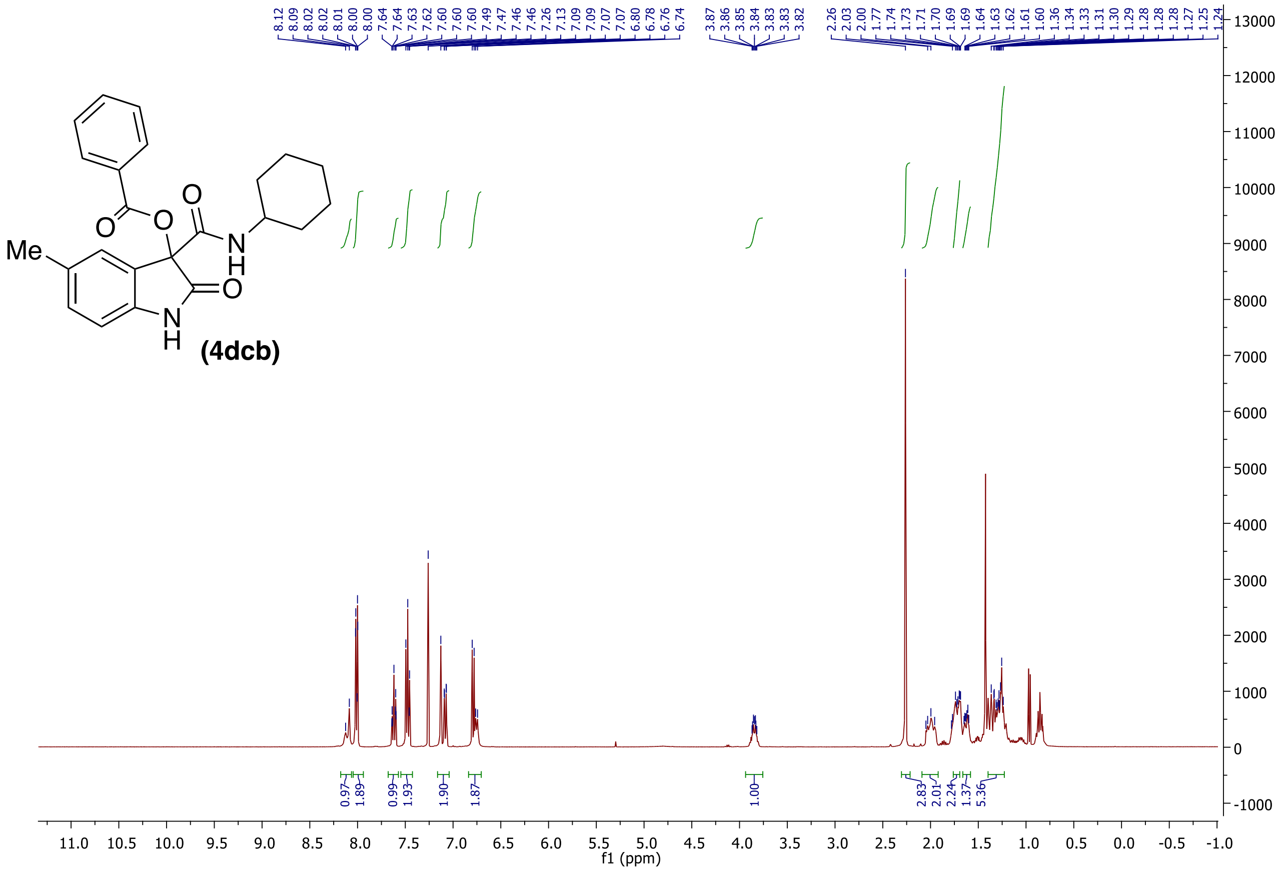

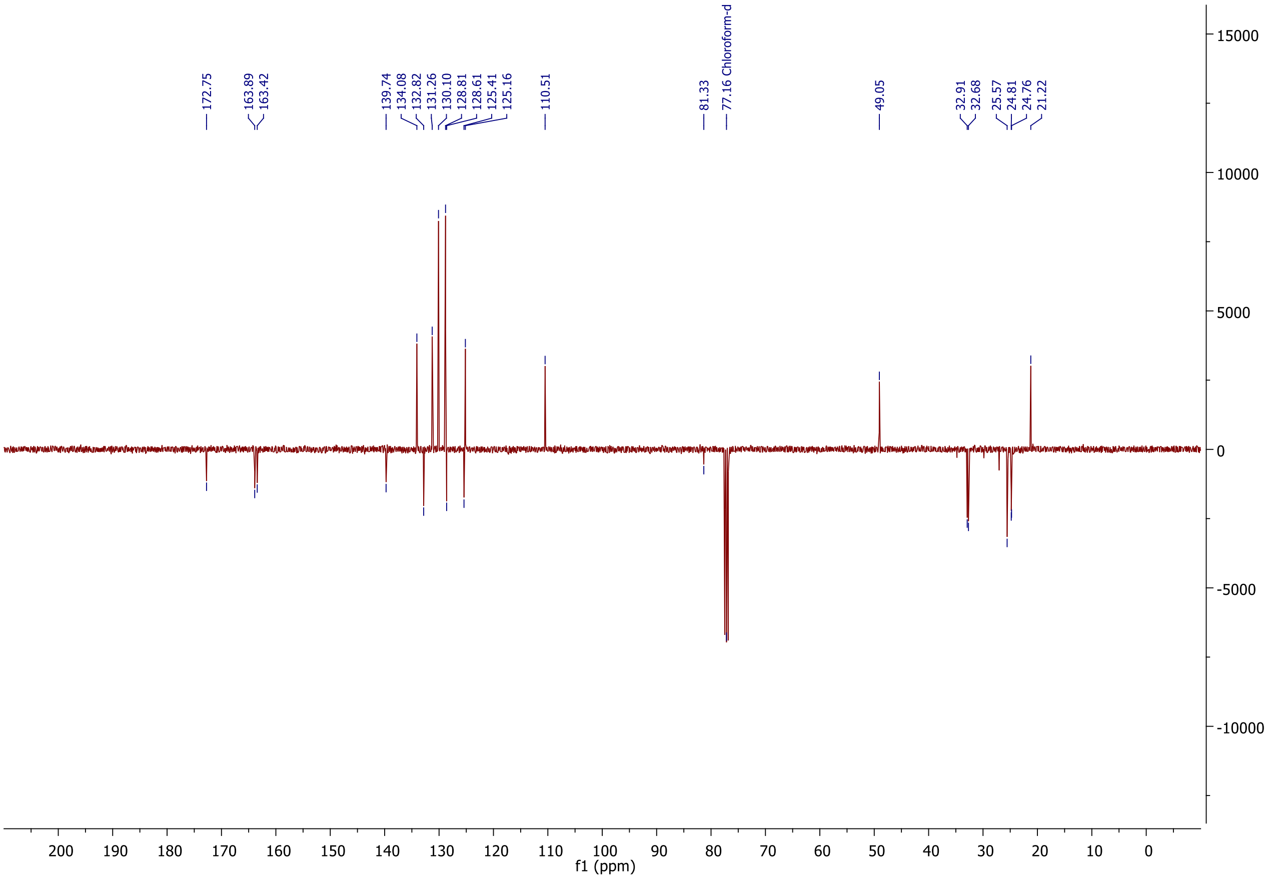

^{1}H and ^{13}C NMR spectra

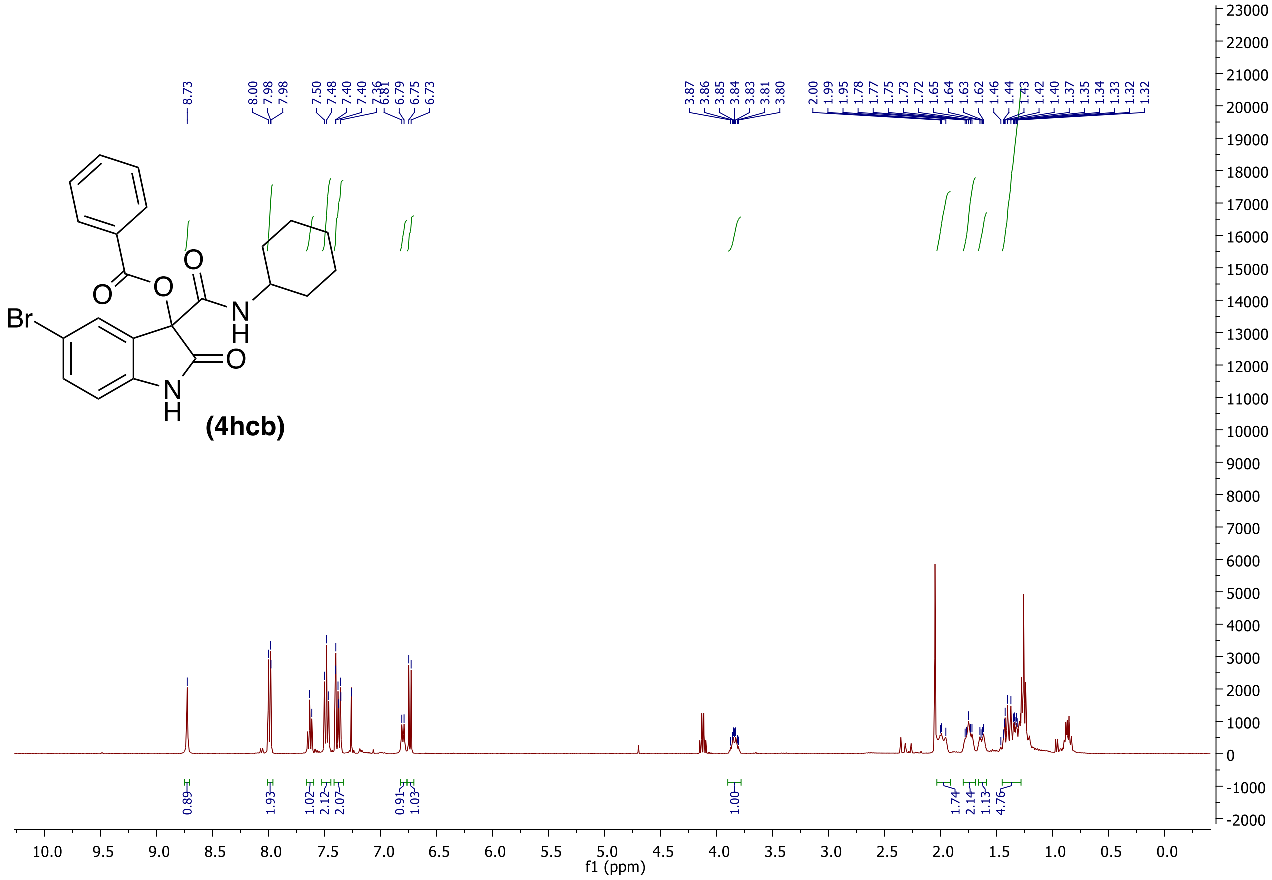

(4hcb)

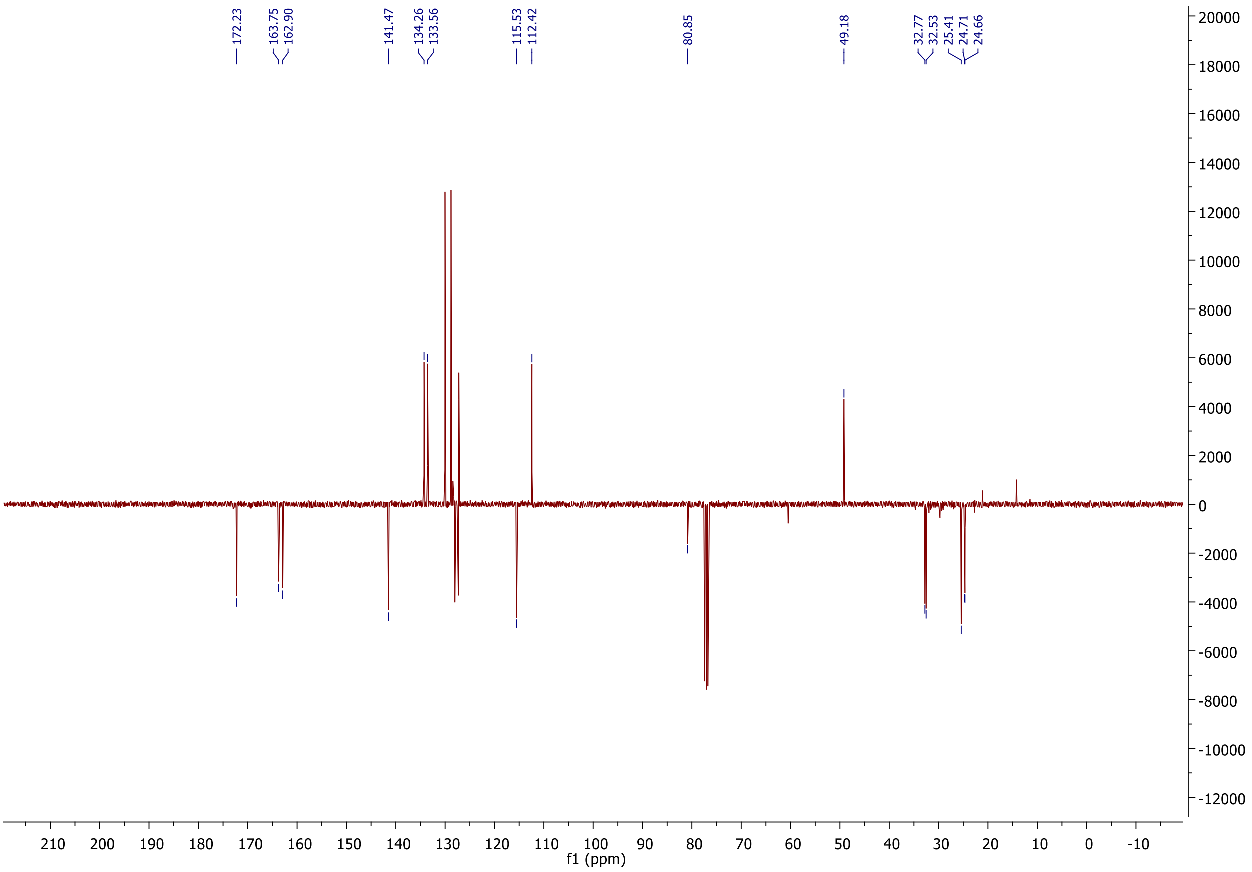

^{1}H and ^{13}C NMR spectra

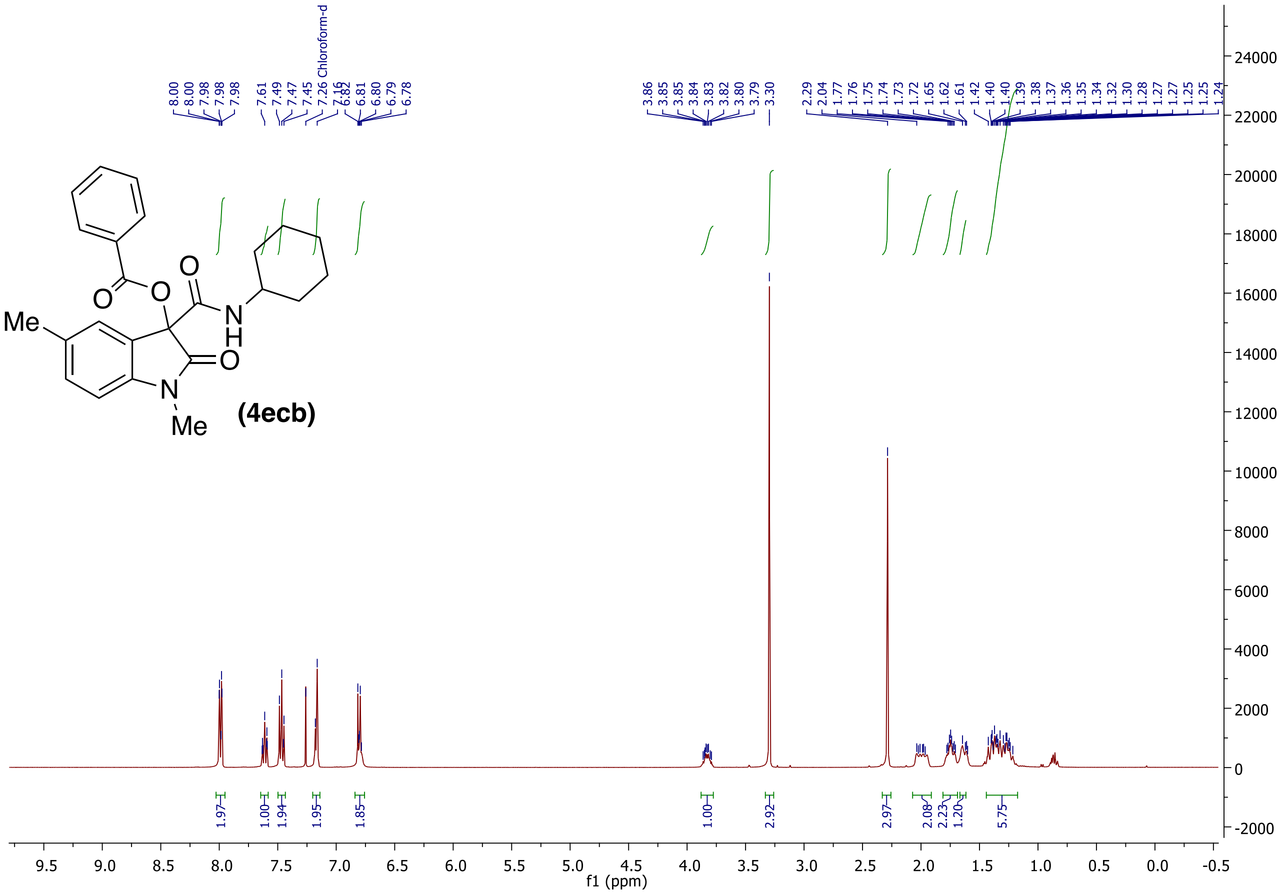

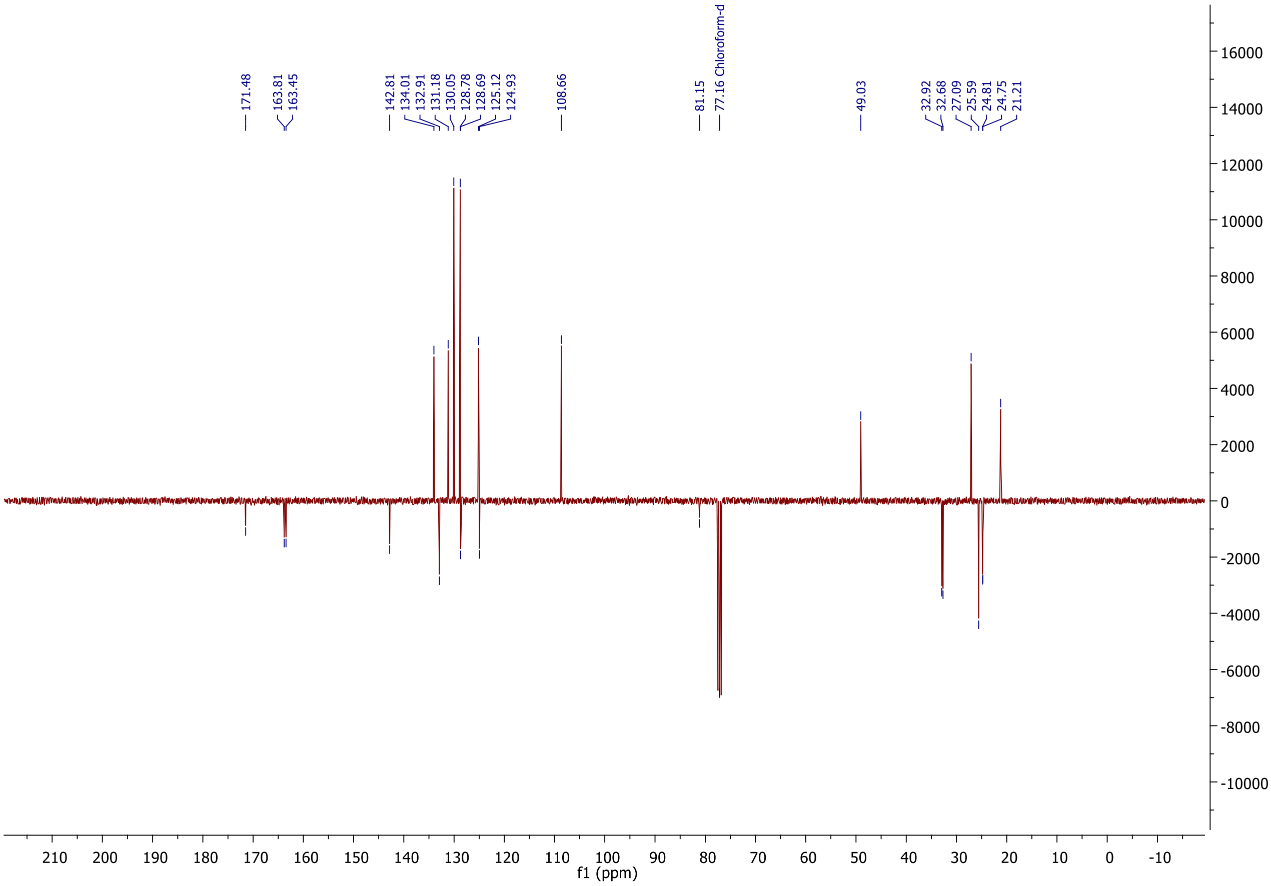

^1H and ^{13}C NMR spectra

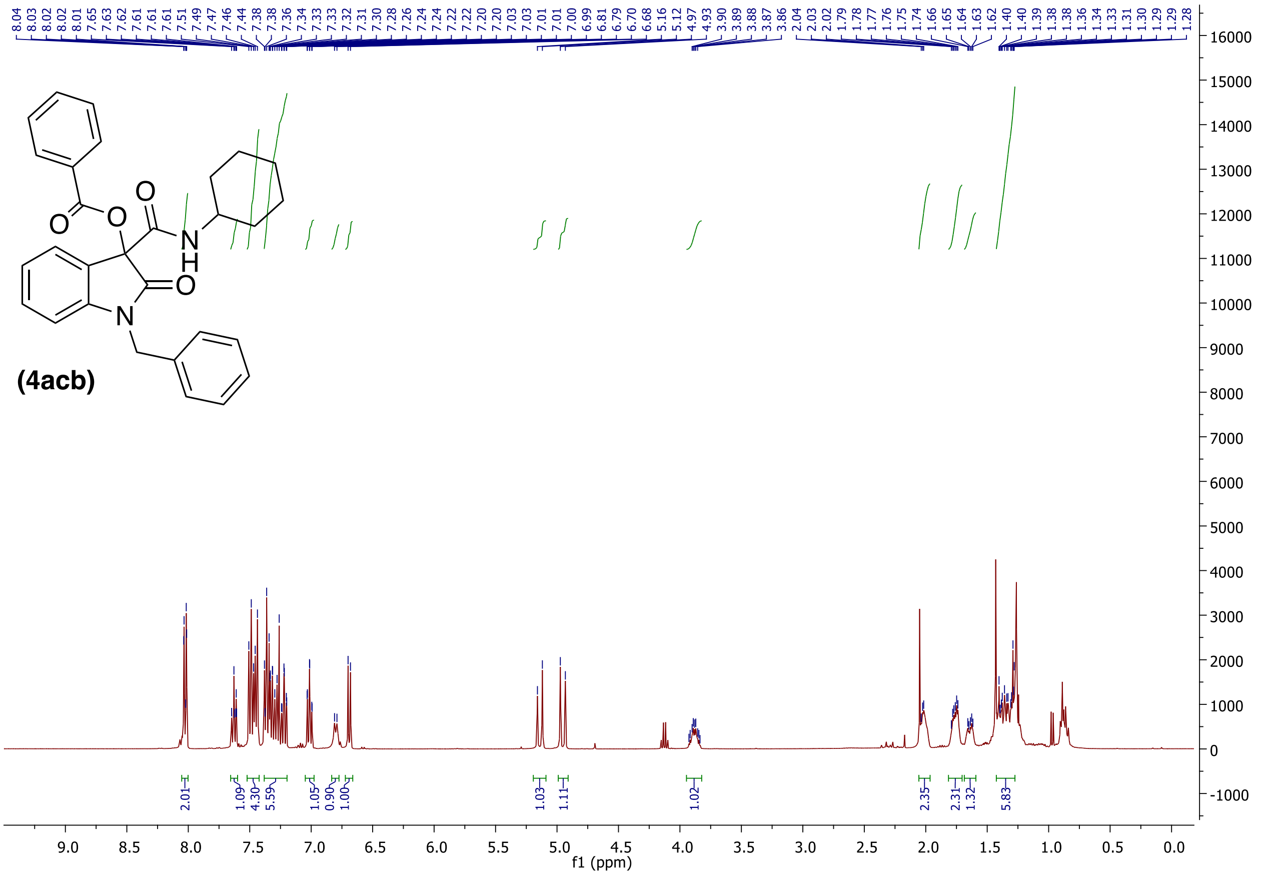

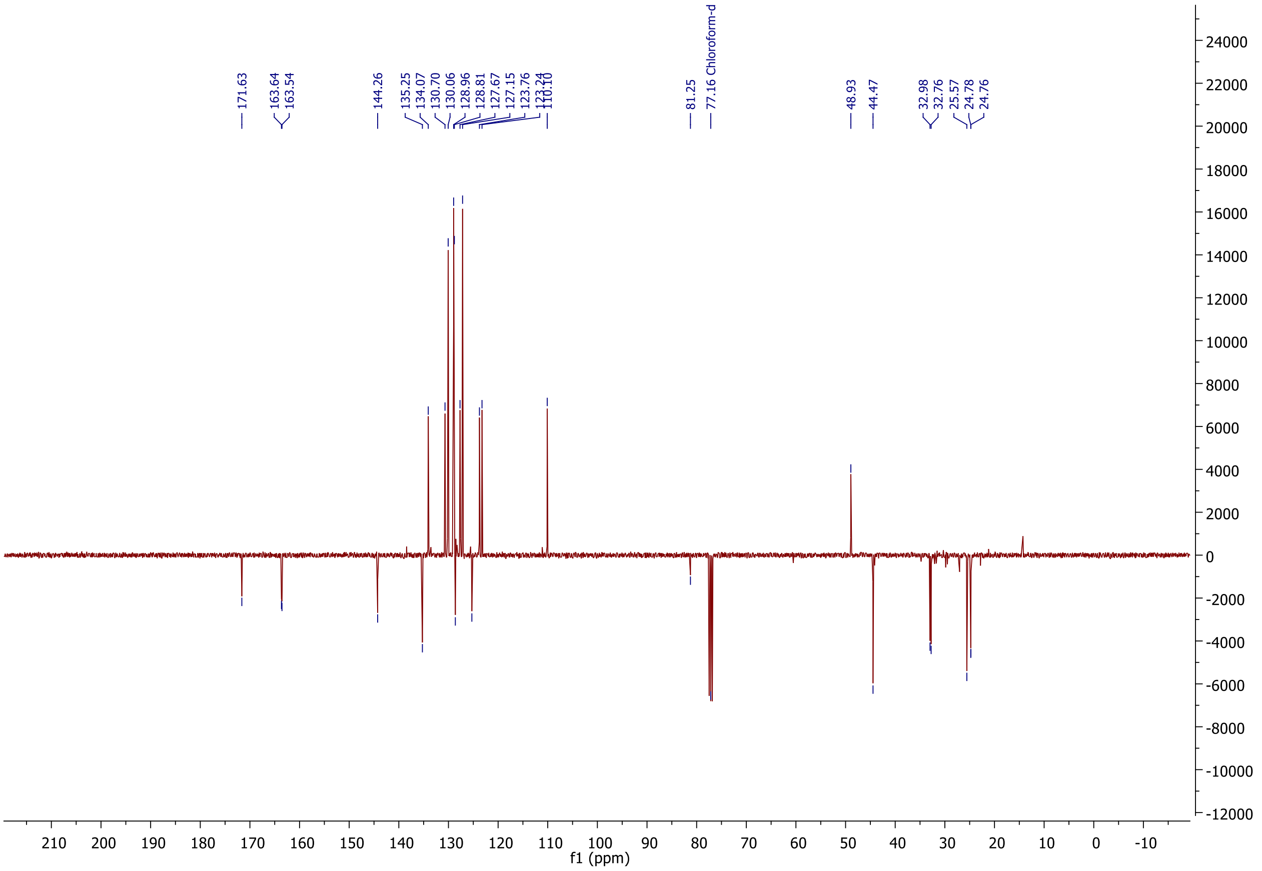

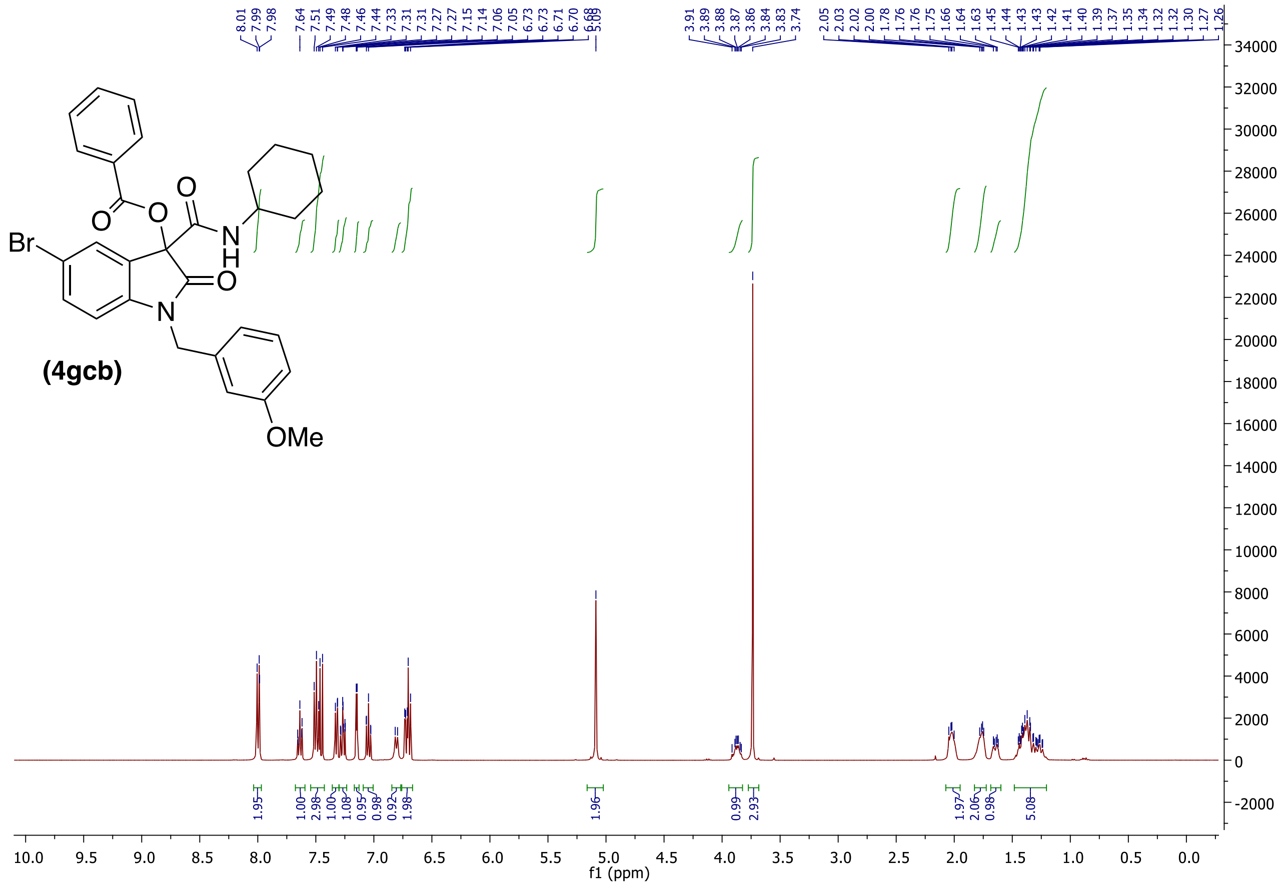
8.01
7.99
7.98
7.64
7.51
7.49
7.48
7.46
7.44
7.33
7.31
7.31
7.27
7.27
7.15
7.14
7.06
7.05
6.73
6.73
6.71
6.70
6.68
5.09
3.91
3.89
3.88
3.87
3.86
3.84
3.83
3.74
2.05
2.03
2.02
2.00
1.78
1.76
1.76
1.75
1.66
1.64
1.63
1.45
1.44
1.43
1.43
1.42
1.41
1.40
1.39
1.37
1.35
1.34
1.32
1.32
1.30
1.27
1.26
Br
O
O
O
N
H
O
N
OMe
(4gcb)
1.95
1.00
2.98
1.00
1.08
0.95
0.98
0.92
1.98
1.96
0.99
2.93
1.97
2.06
0.98
5.08
f1 (ppm)

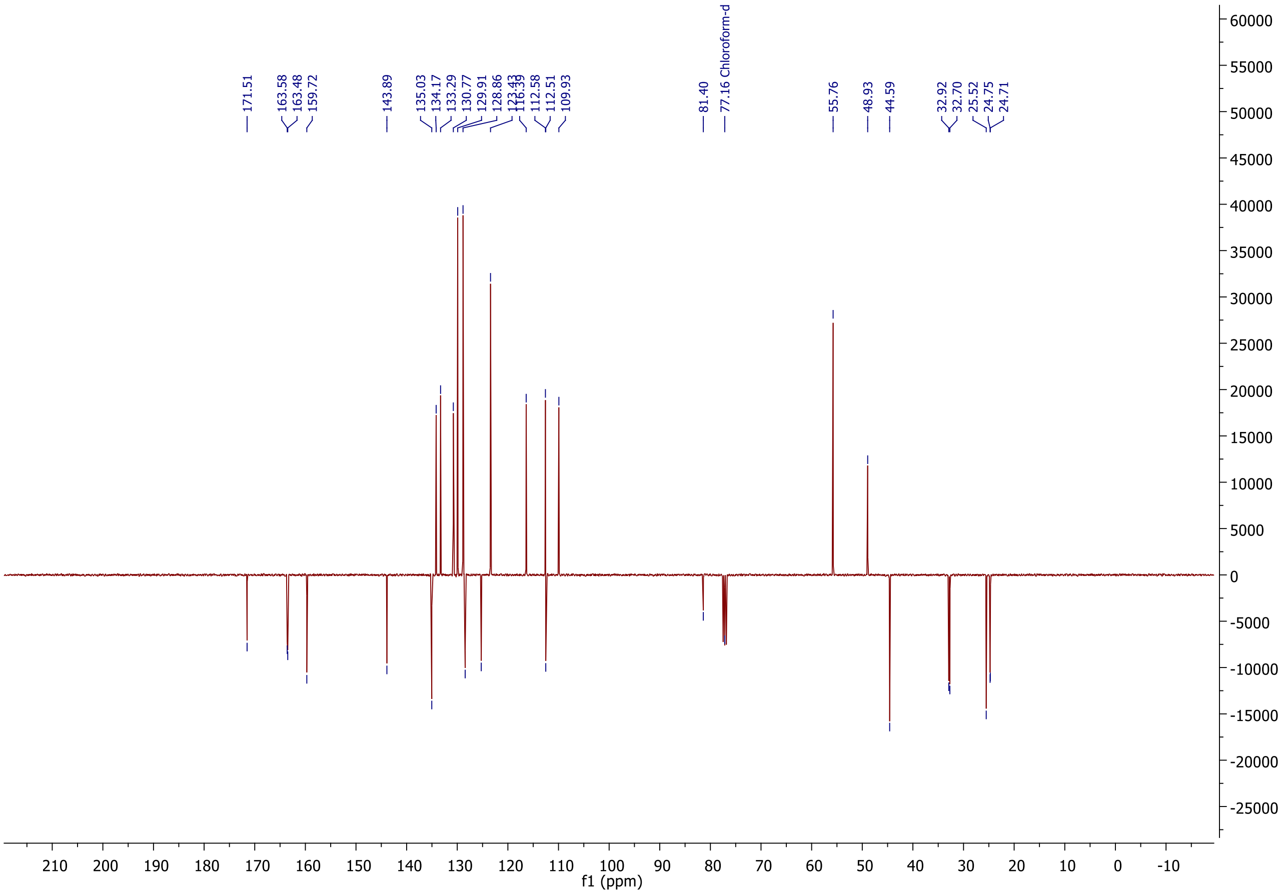
171.51
163.58
163.48
159.72
143.89
135.03
134.17
133.29
130.77
129.91
128.86
123.43
116.39
112.58
112.51
109.93
81.40
77.16 Chloroform-d
55.76
48.93
44.59
32.92
32.70
25.52
24.75
24.71
f1 (ppm)

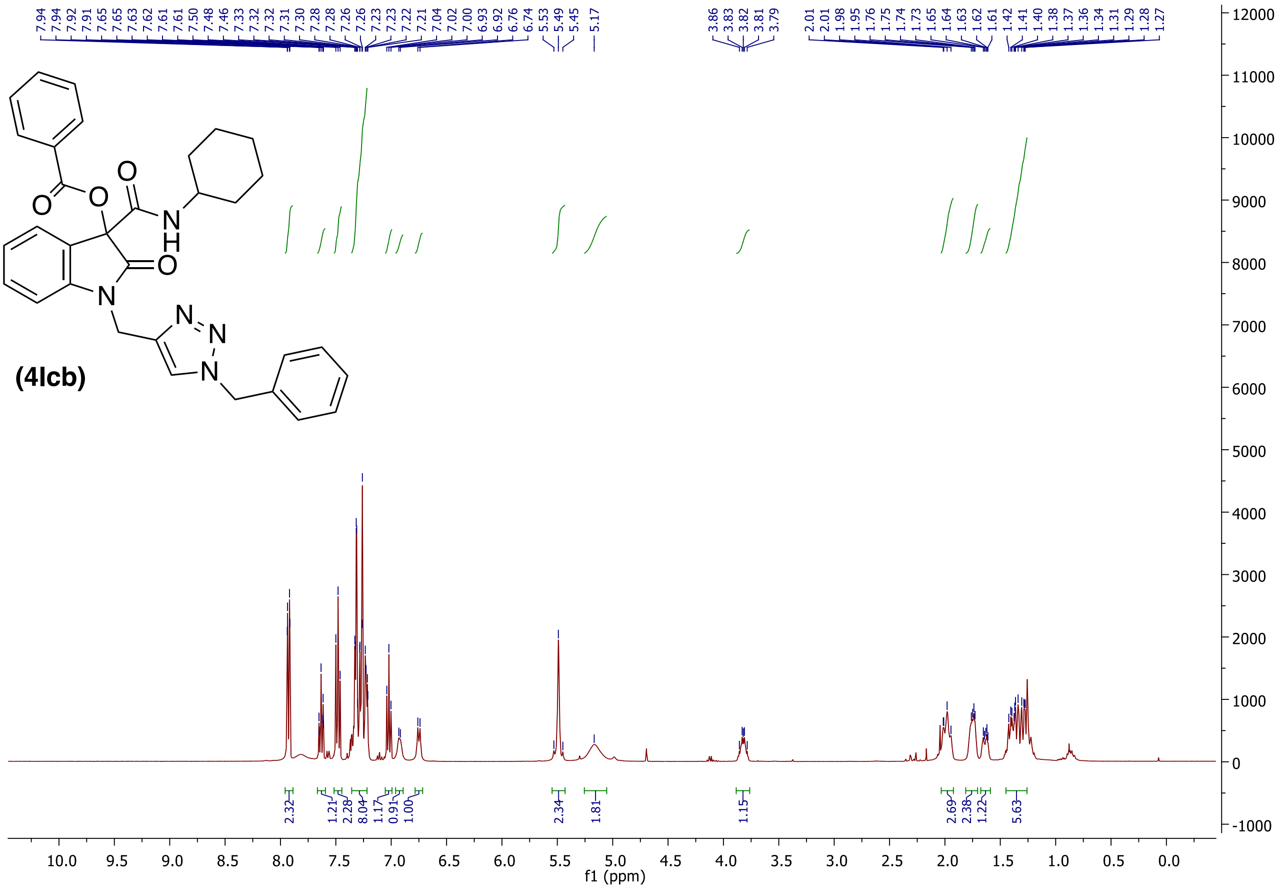
7.94
7.94
7.92
7.91
7.65
7.63
7.62
7.61
7.61
7.50
7.48
7.46
7.33
7.32
7.32
7.31
7.30
7.28
7.28
7.26
7.26
7.23
7.23
7.22
7.21
7.04
7.02
7.00
6.93
6.92
6.76
6.74
5.53
5.49
5.45
5.17
3.86
3.83
3.82
3.81
3.79
2.01
2.01
1.98
1.95
1.76
1.75
1.74
1.73
1.65
1.64
1.63
1.62
1.61
1.42
1.41
1.40
1.38
1.37
1.36
1.34
1.31
1.29
1.28
1.27
(4lcb)
2.32
1.21
2.28
8.04
1.17
0.91
1.00
2.34
1.81
1.15
2.69
2.38
1.22
5.63
f1 (ppm)

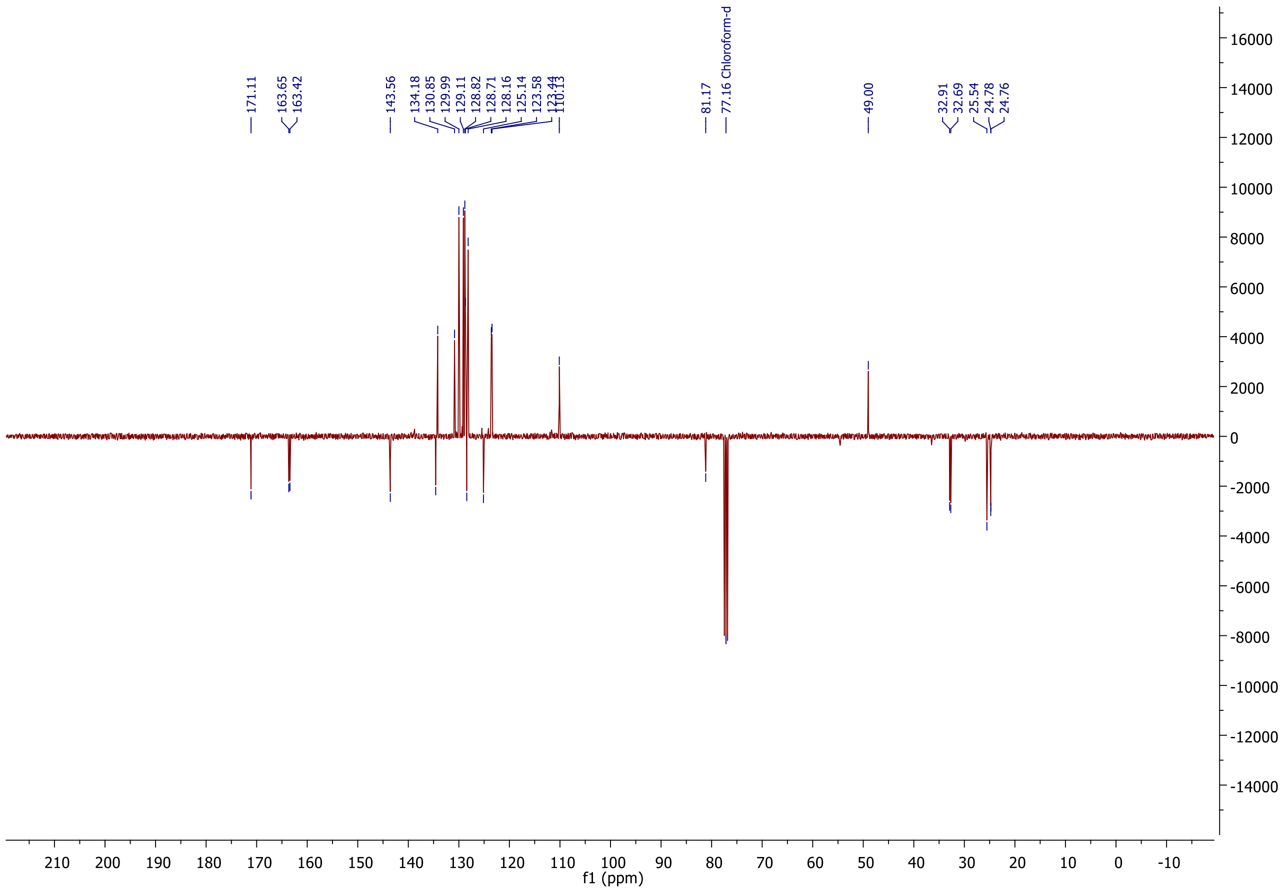
171.11
163.65
163.42
143.56
134.18
130.85
129.99
129.11
128.82
128.71
128.16
125.14
123.58
123.44
110.13
81.17
77.16 Chloroform-d
49.00
32.91
32.69
25.54
24.78
24.76
f1 (ppm)

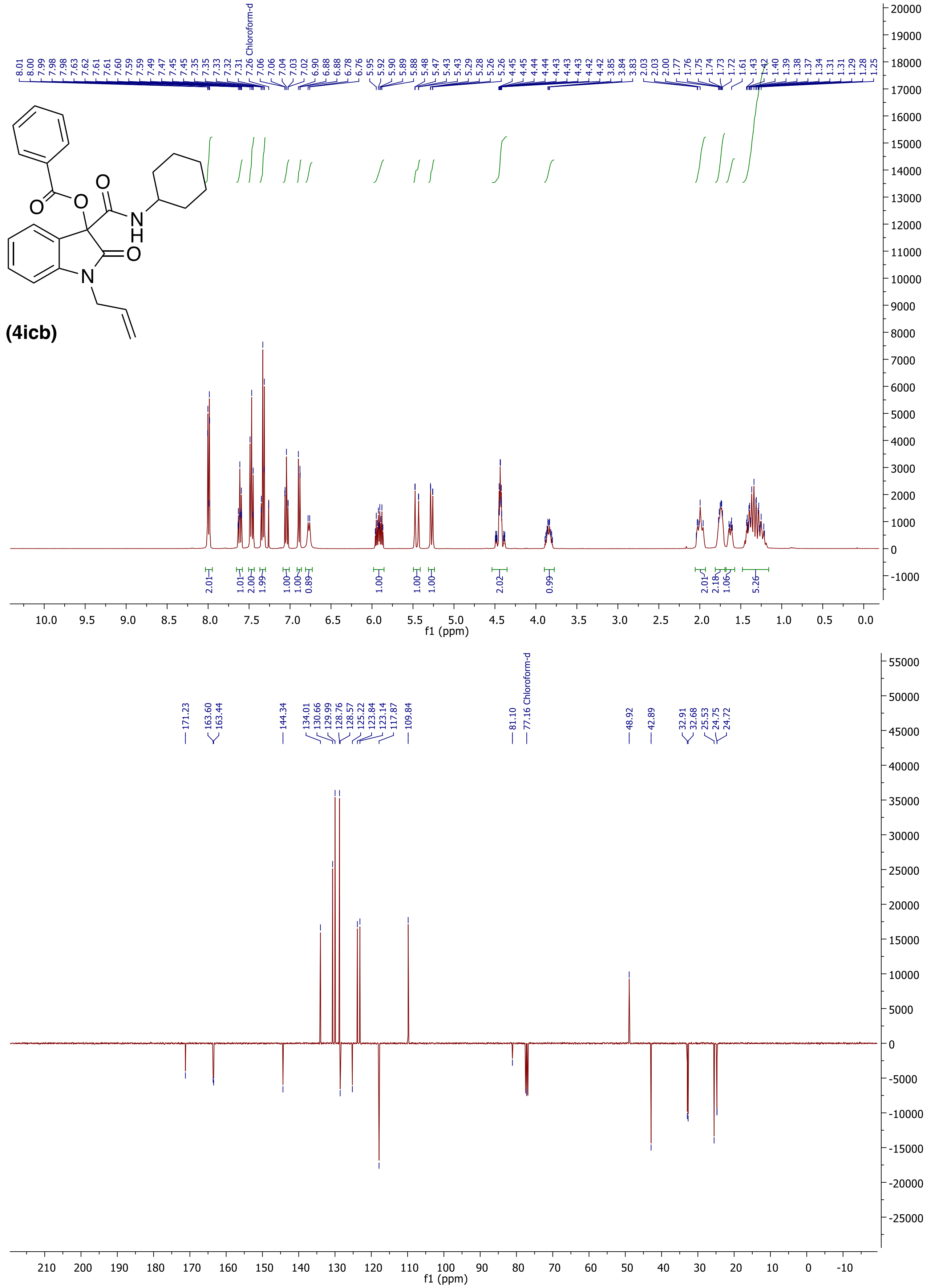

^{1}H and ^{13}C NMR spectra

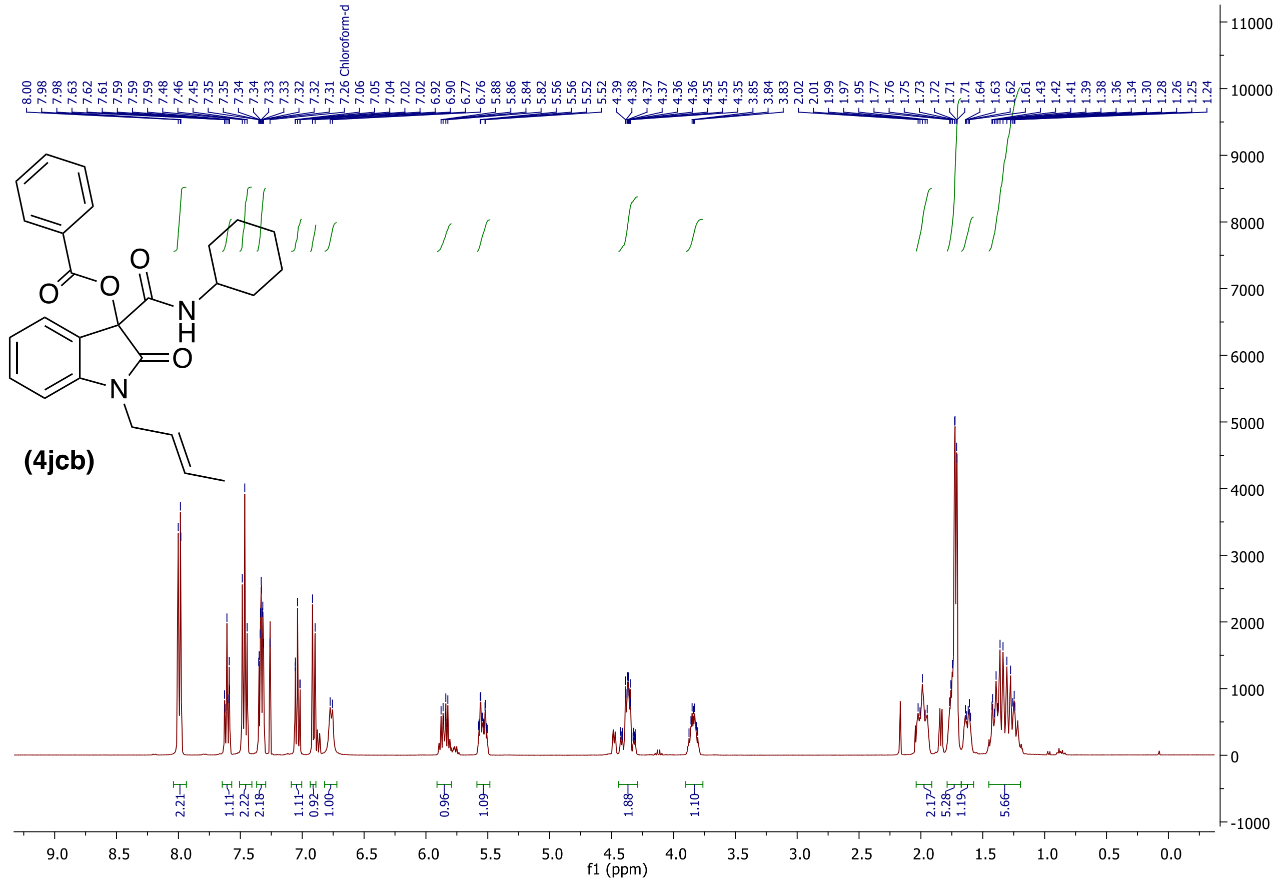

(4jcb)

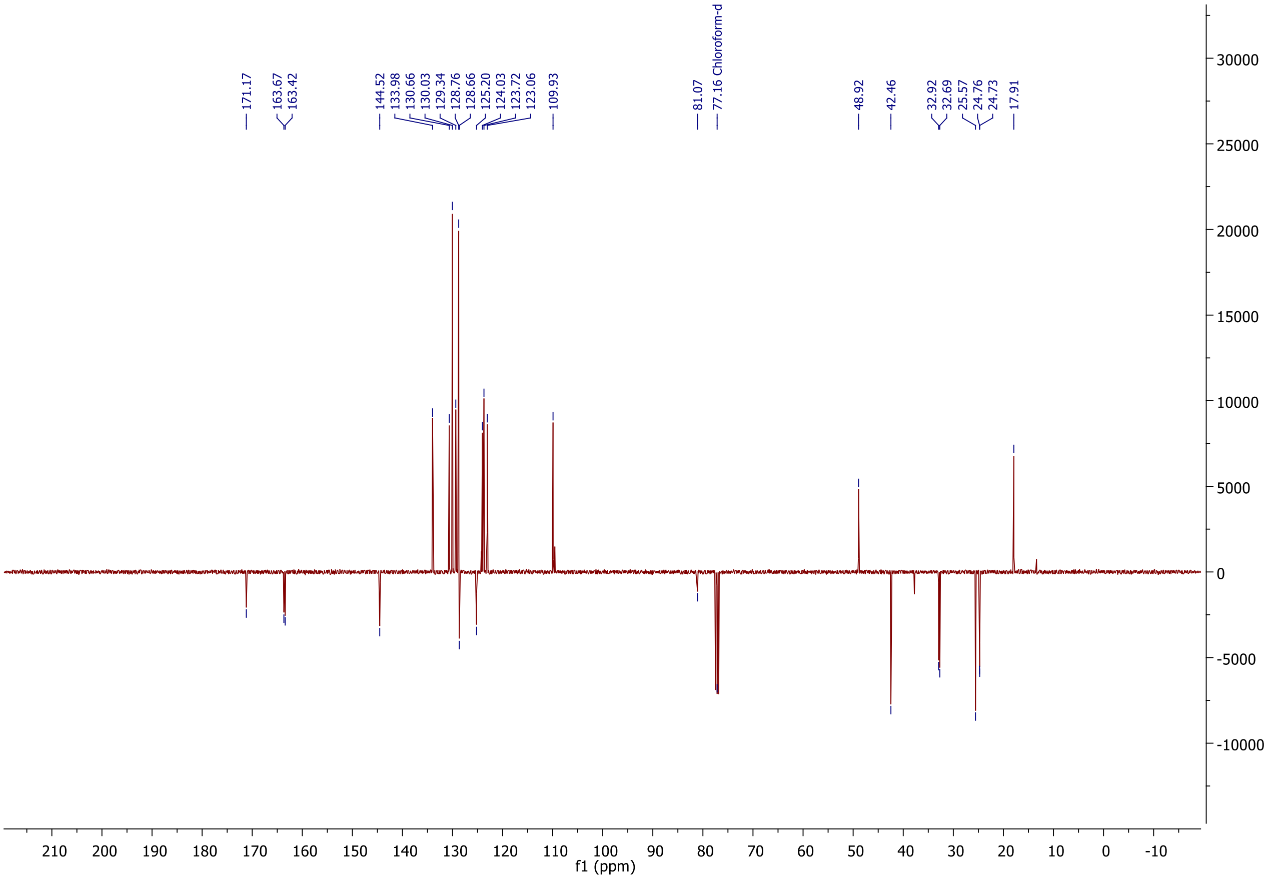

^1H and ^{13}C NMR spectra

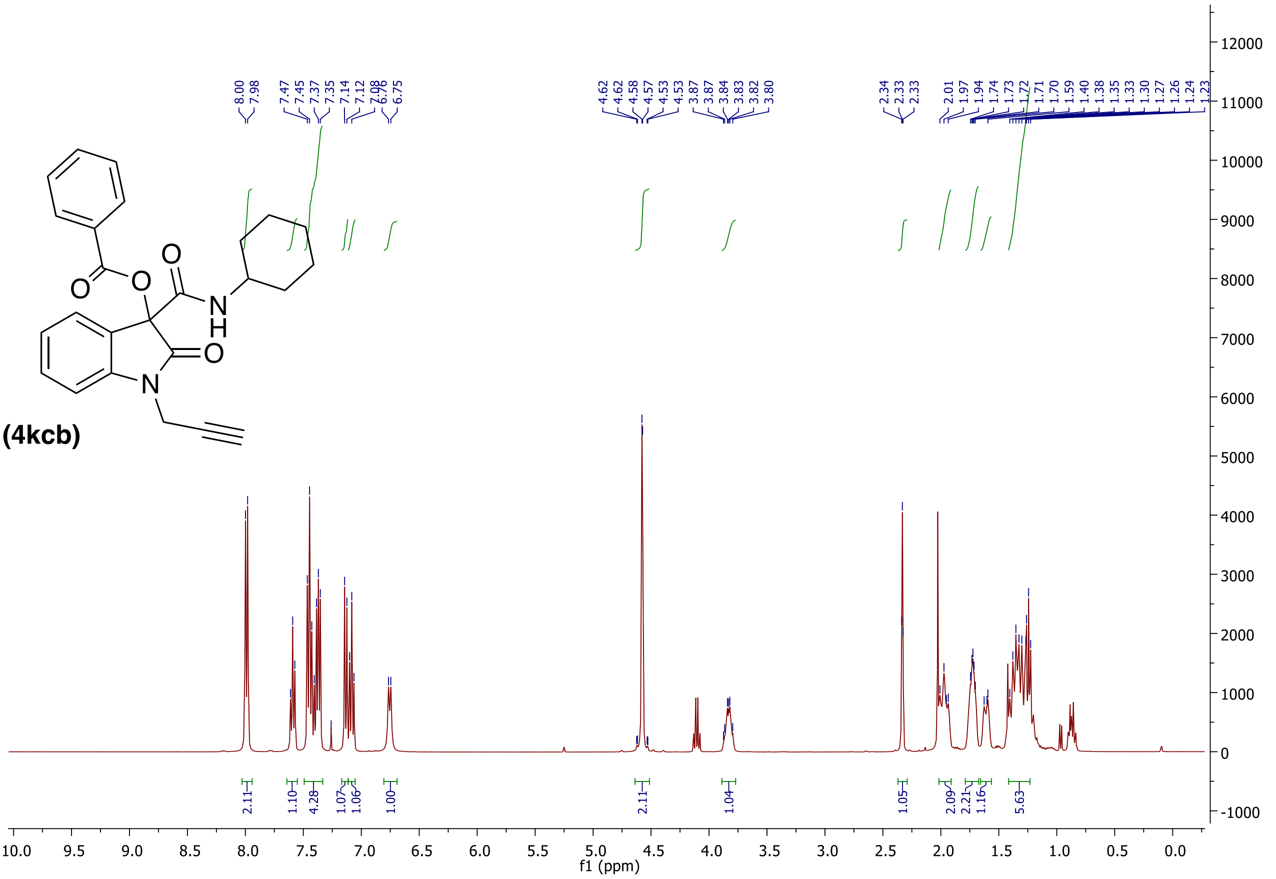

(4kcb)

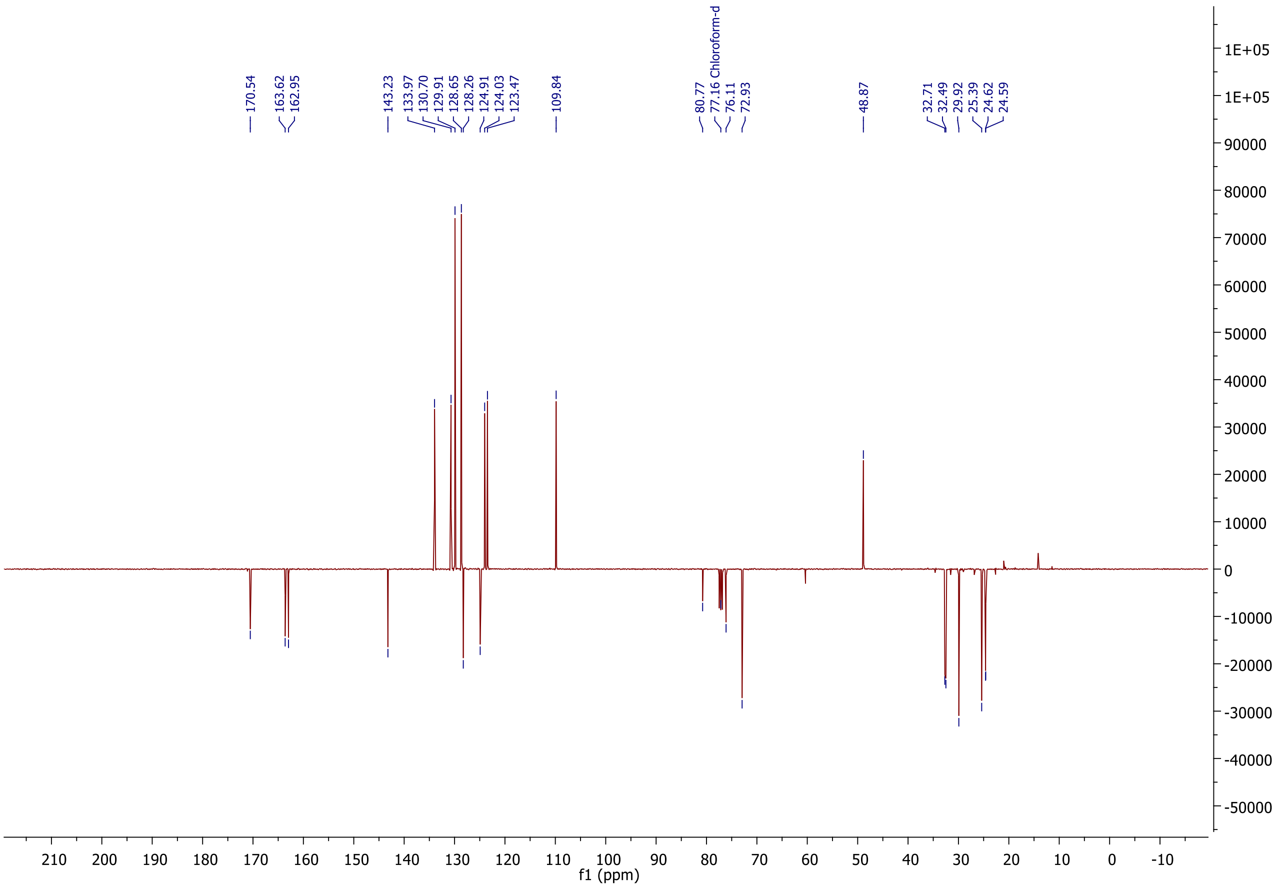

^{1}H and ^{13}C NMR spectra

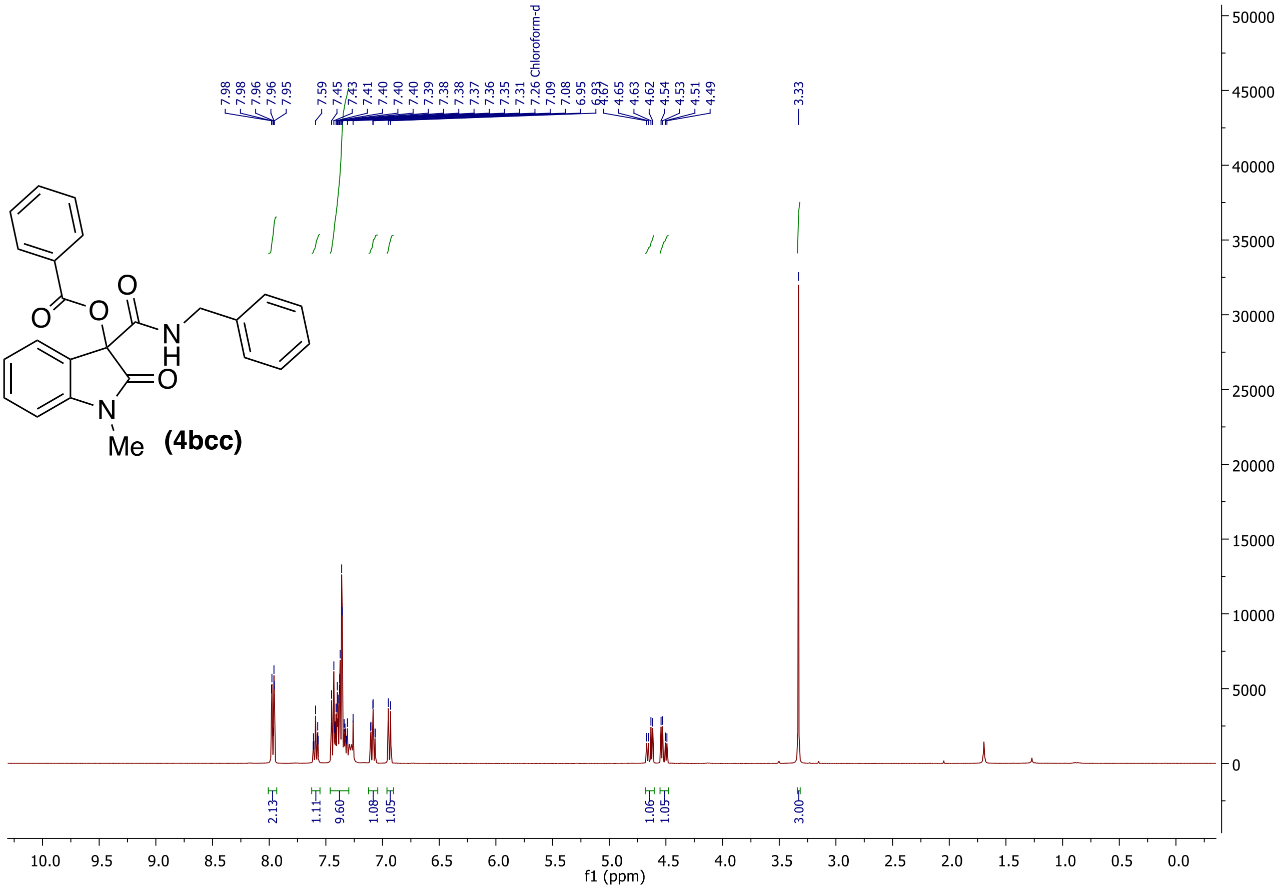

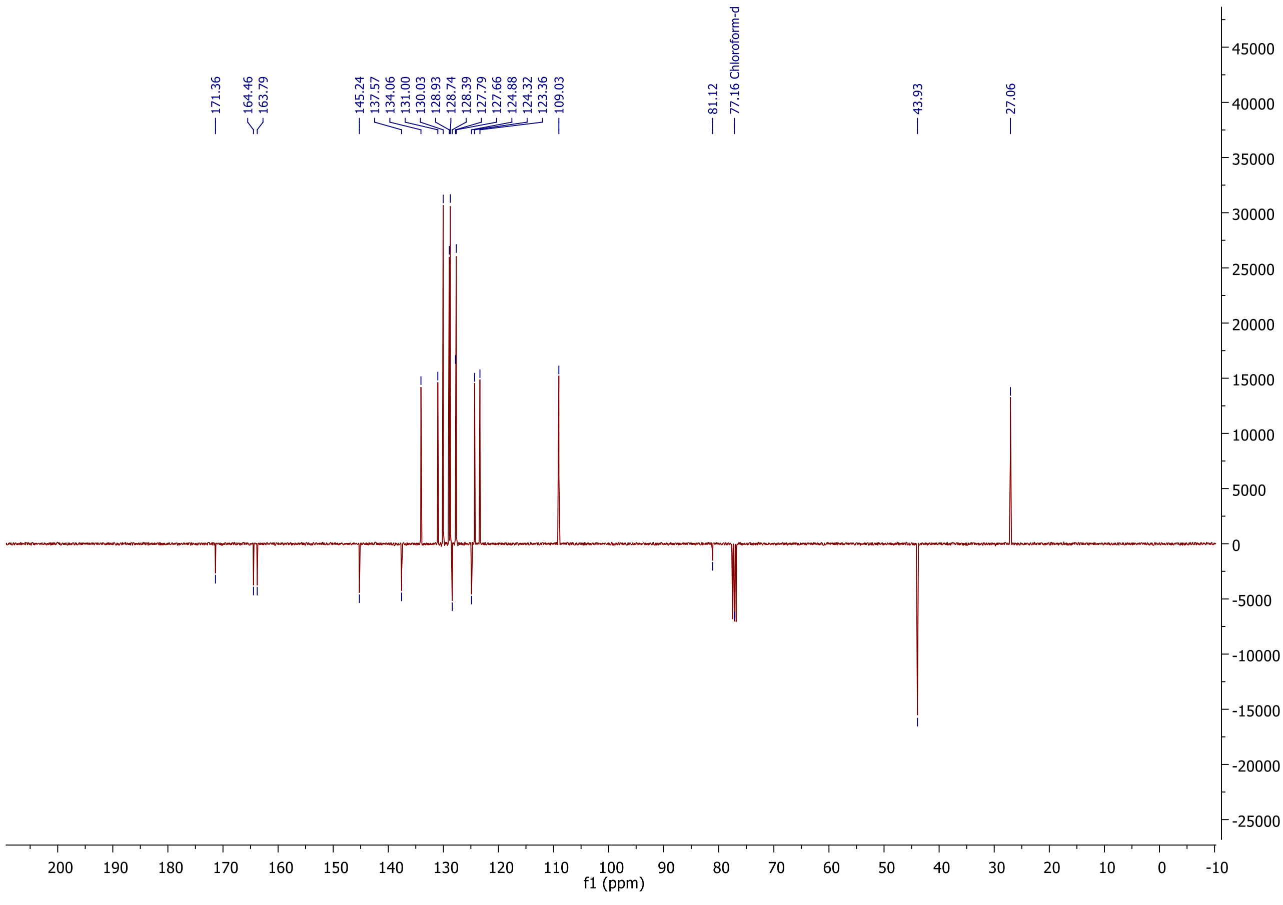

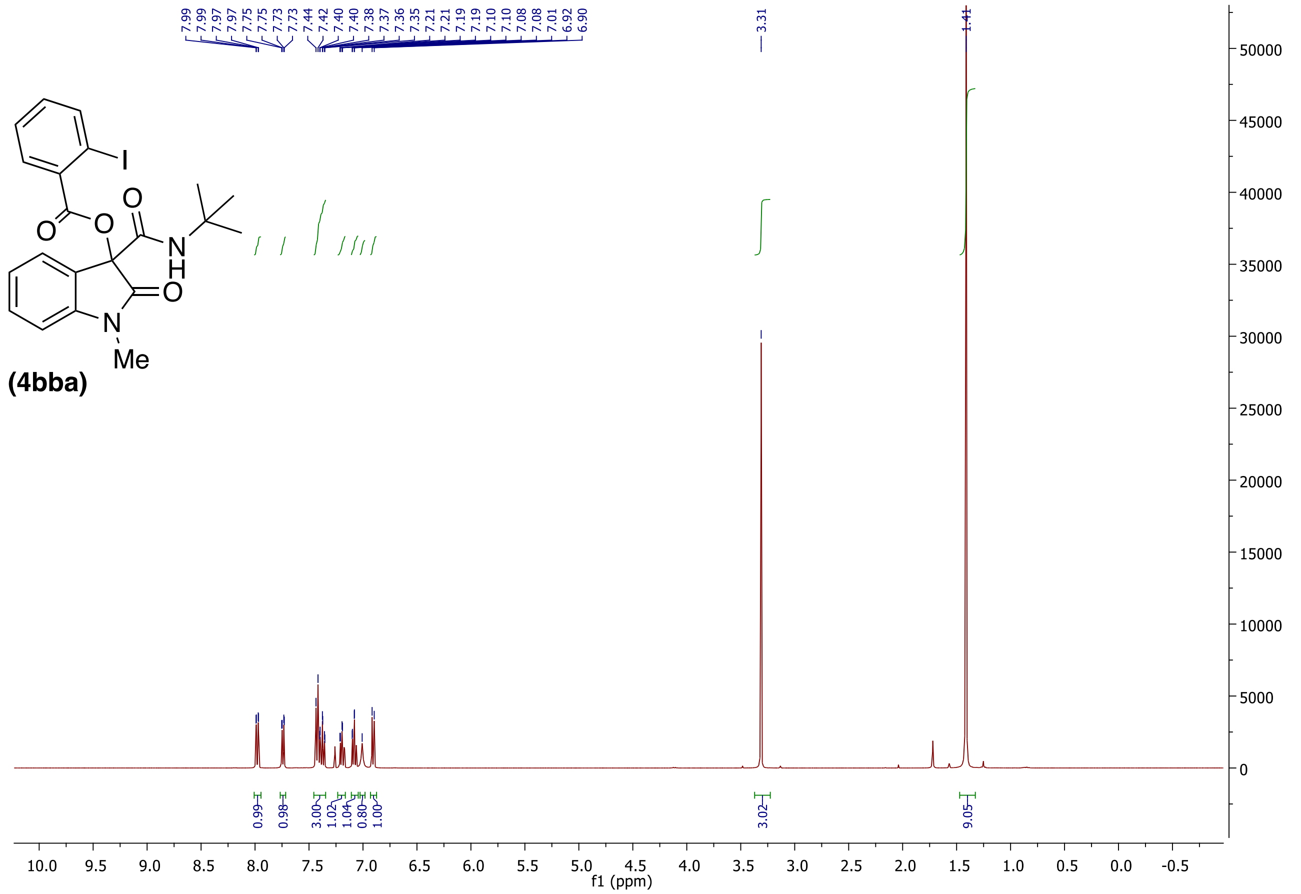

O
O
I
N
H
N
O
Me
(4bba)
7.99 7.99 7.97 7.97 7.75 7.75 7.73 7.73 7.44 7.42 7.40 7.40 7.38 7.37 7.36 7.35 7.21 7.21 7.19 7.19 7.10 7.10 7.08 7.08 7.01 6.92 6.90
3.31
1.41
0.99 0.98 3.00 1.02 1.04 0.80 1.00 3.02 9.05
f1 (ppm)

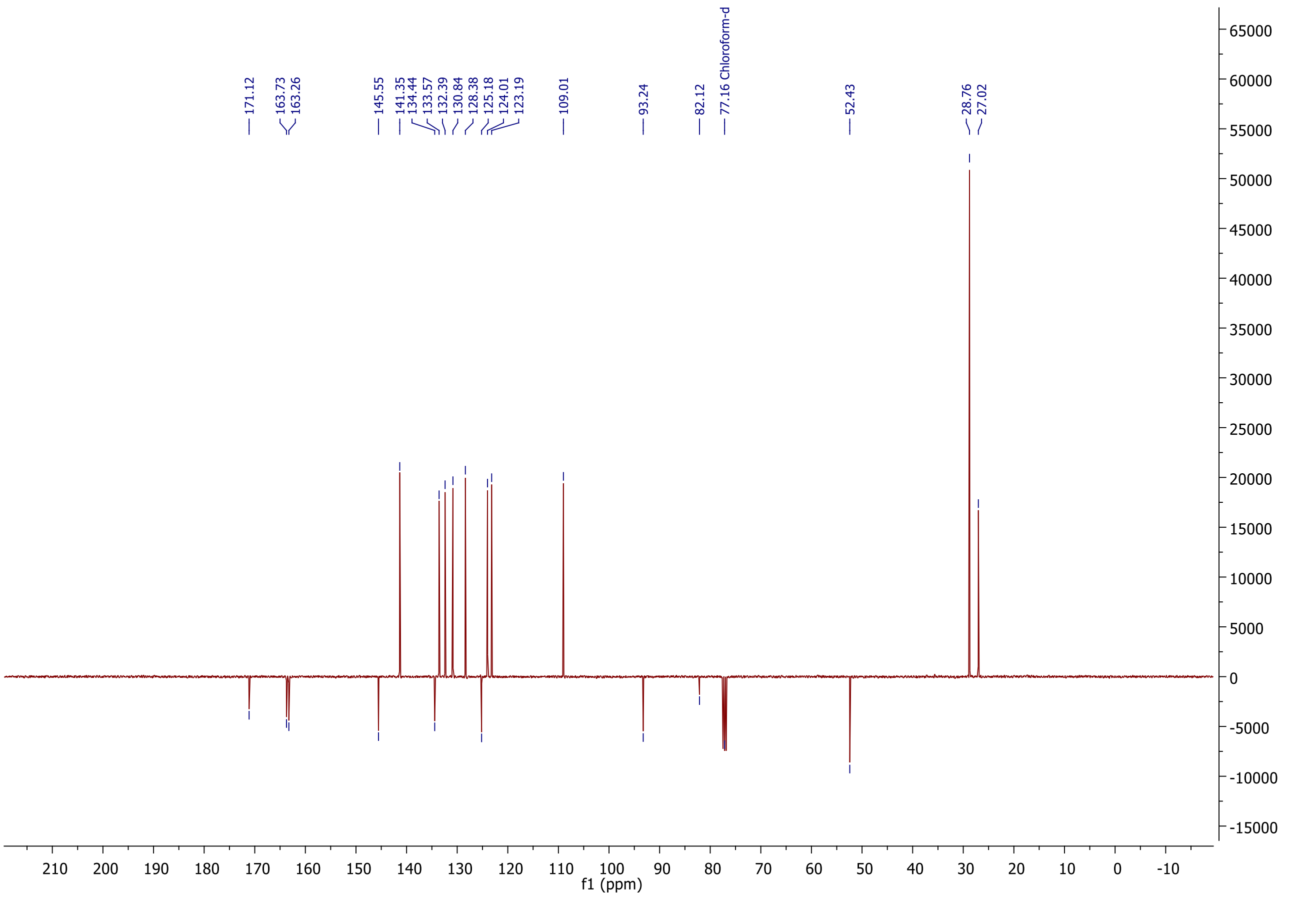

171.12
163.73 163.26
145.55 141.35 134.44 133.57 132.39 130.84 128.38 125.18 124.01 123.19
109.01
93.24
82.12
77.16 Chloroform-d
52.43
28.76 27.02
f1 (ppm)

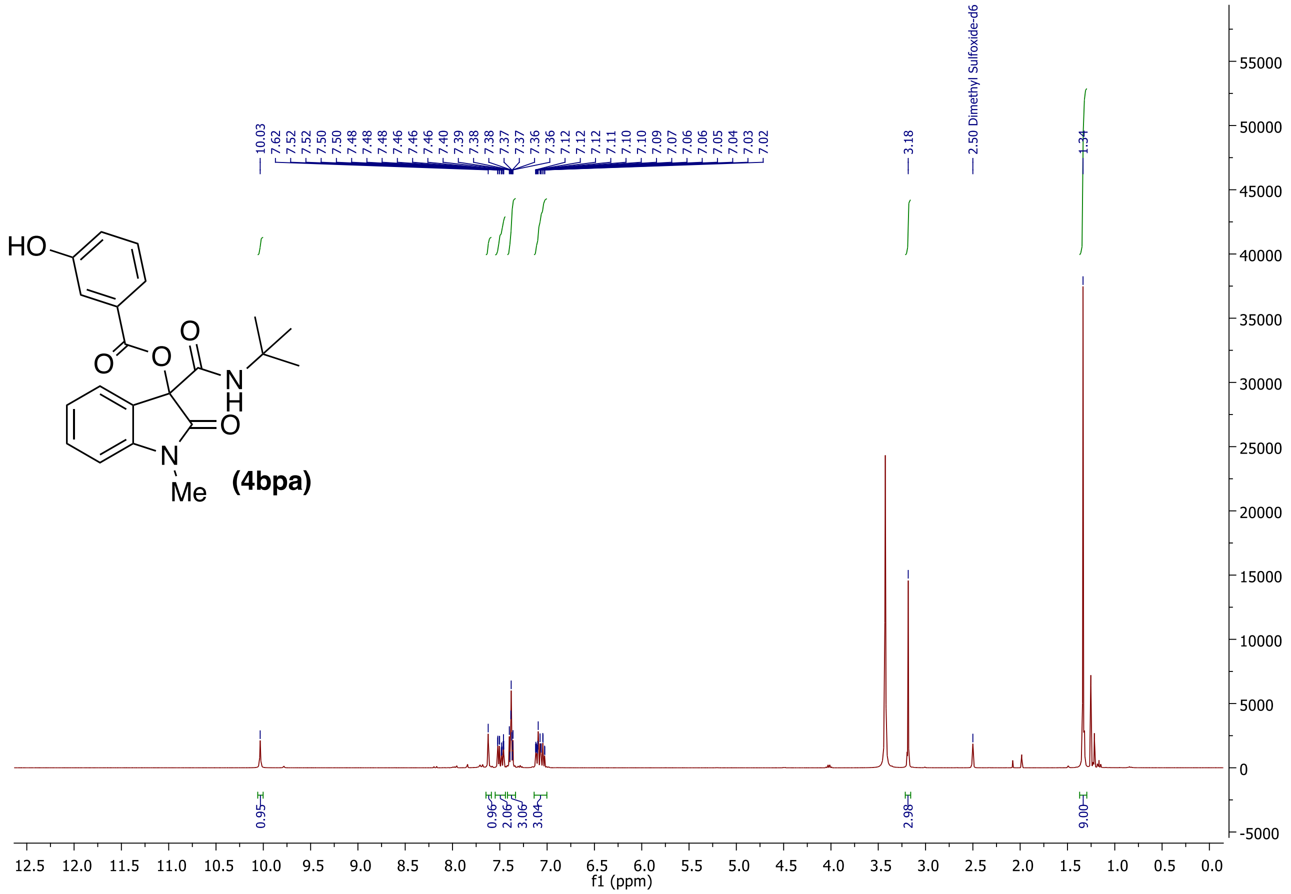

(4bpa)
10.03
7.62
7.52
7.52
7.50
7.50
7.48
7.48
7.46
7.46
7.46
7.40
7.39
7.38
7.38
7.37
7.37
7.36
7.36
7.12
7.12
7.12
7.11
7.10
7.10
7.09
7.07
7.06
7.06
7.05
7.04
7.03
7.02
3.18
2.50 Dimethyl Sulfoxide-d6
1.34
0.95
0.96
2.06
3.06
3.04
2.98
9.00
f1 (ppm)

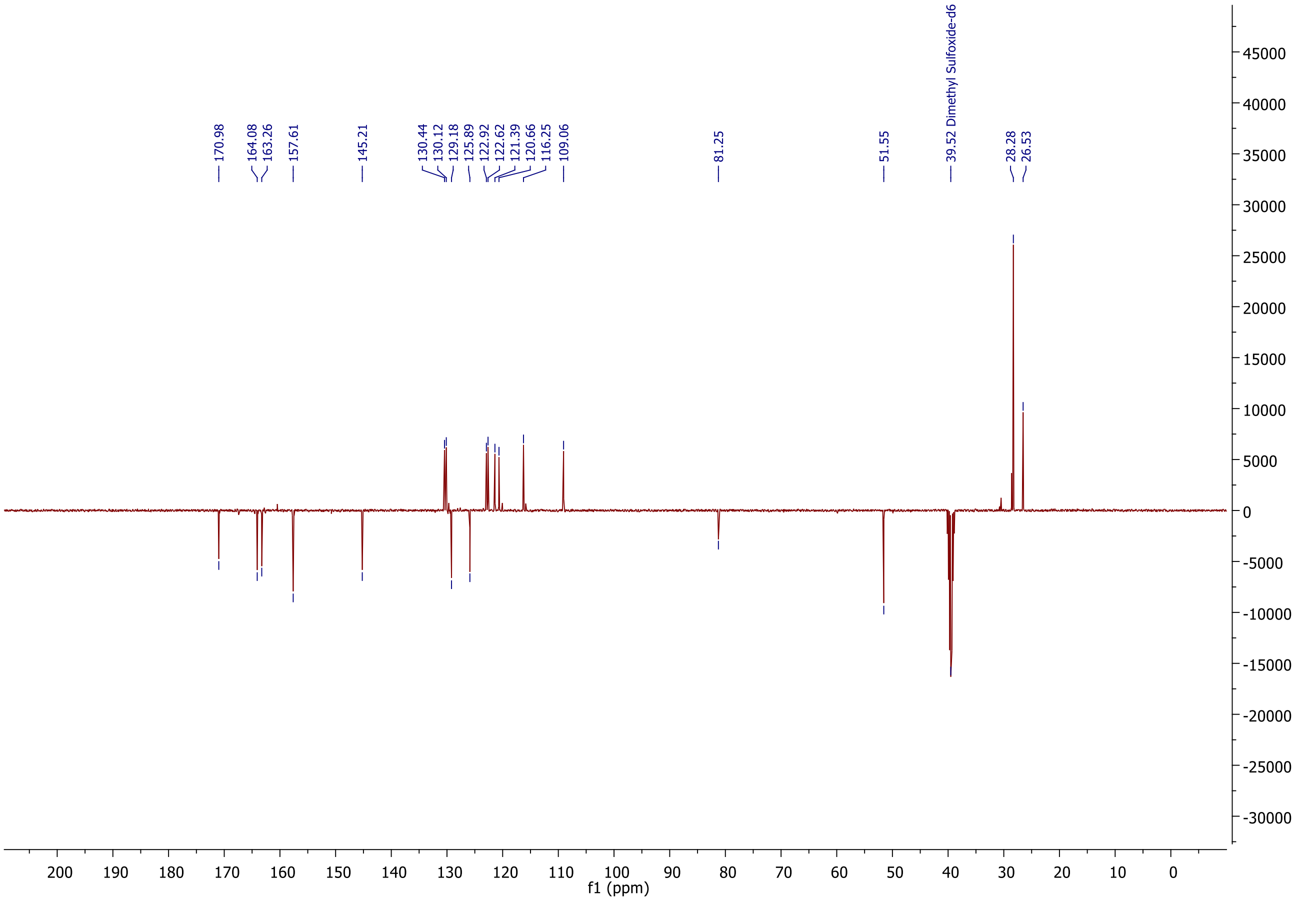
170.98
164.08
163.26
157.61
145.21
130.44
130.12
129.18
125.89
122.92
122.62
121.39
120.66
116.25
109.06
81.25
51.55
39.52 Dimethyl Sulfoxide-d6
28.28
26.53
f1 (ppm)

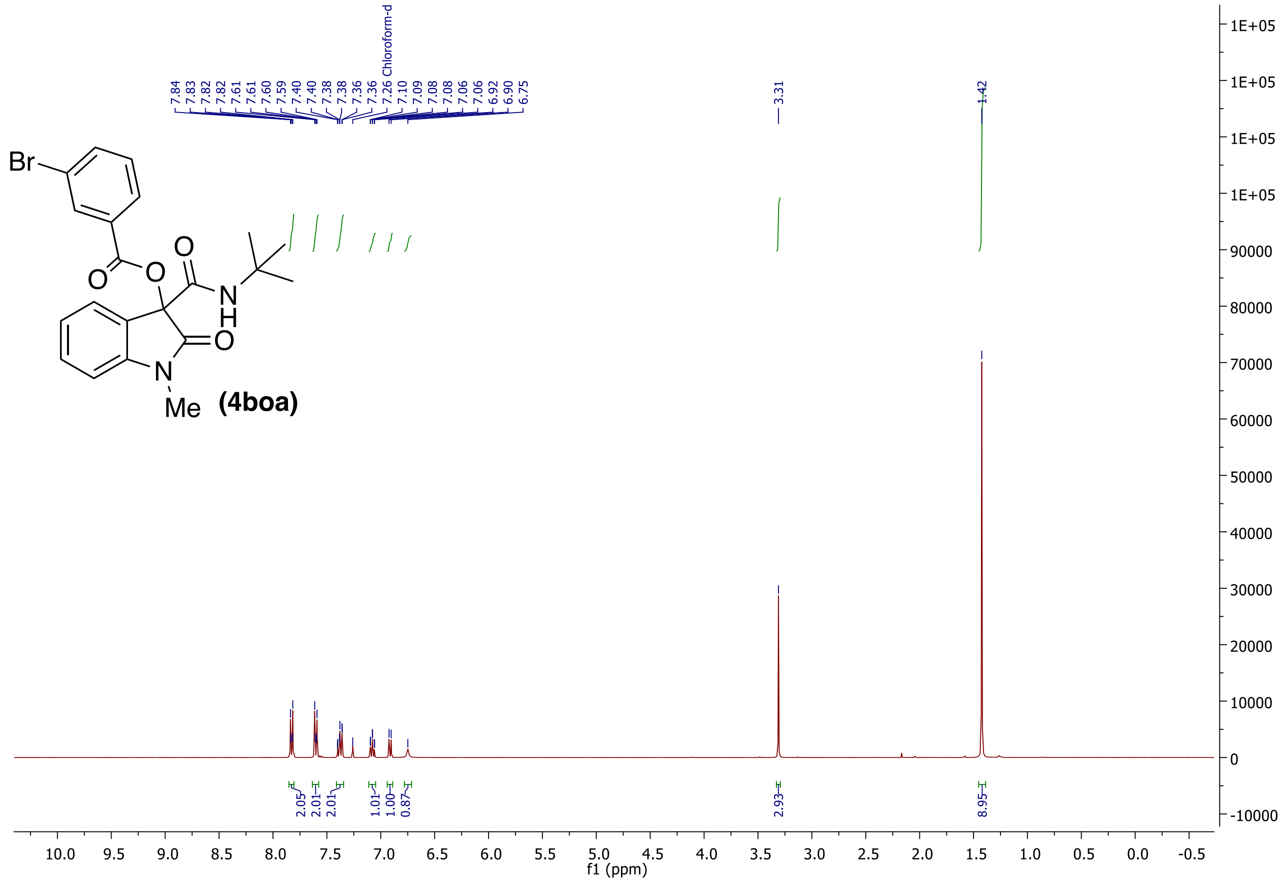
(4boa)
7.84
7.83
7.82
7.82
7.61
7.61
7.60
7.59
7.40
7.40
7.38
7.38
7.36
7.36
7.26 Chloroform-d
7.10
7.09
7.08
7.08
7.06
7.06
6.92
6.90
6.75
3.31
1.42
2.05
2.01
2.01
1.01
1.00
0.87
2.93
8.95
f1 (ppm)

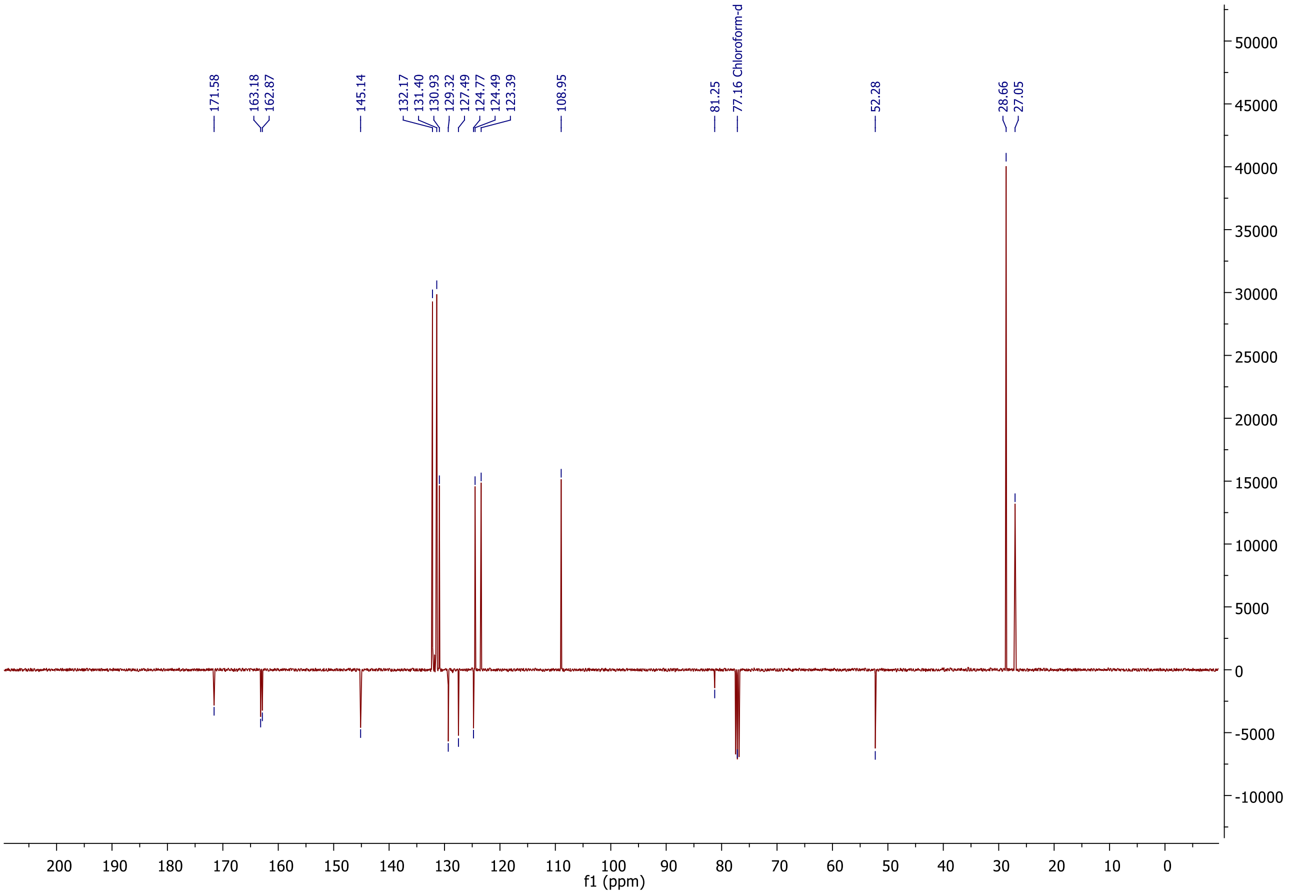
171.58
163.18
162.87
145.14
132.17
131.40
130.93
129.32
127.49
124.77
124.49
123.39
108.95
81.25
77.16 Chloroform-d
52.28
28.66
27.05
f1 (ppm)

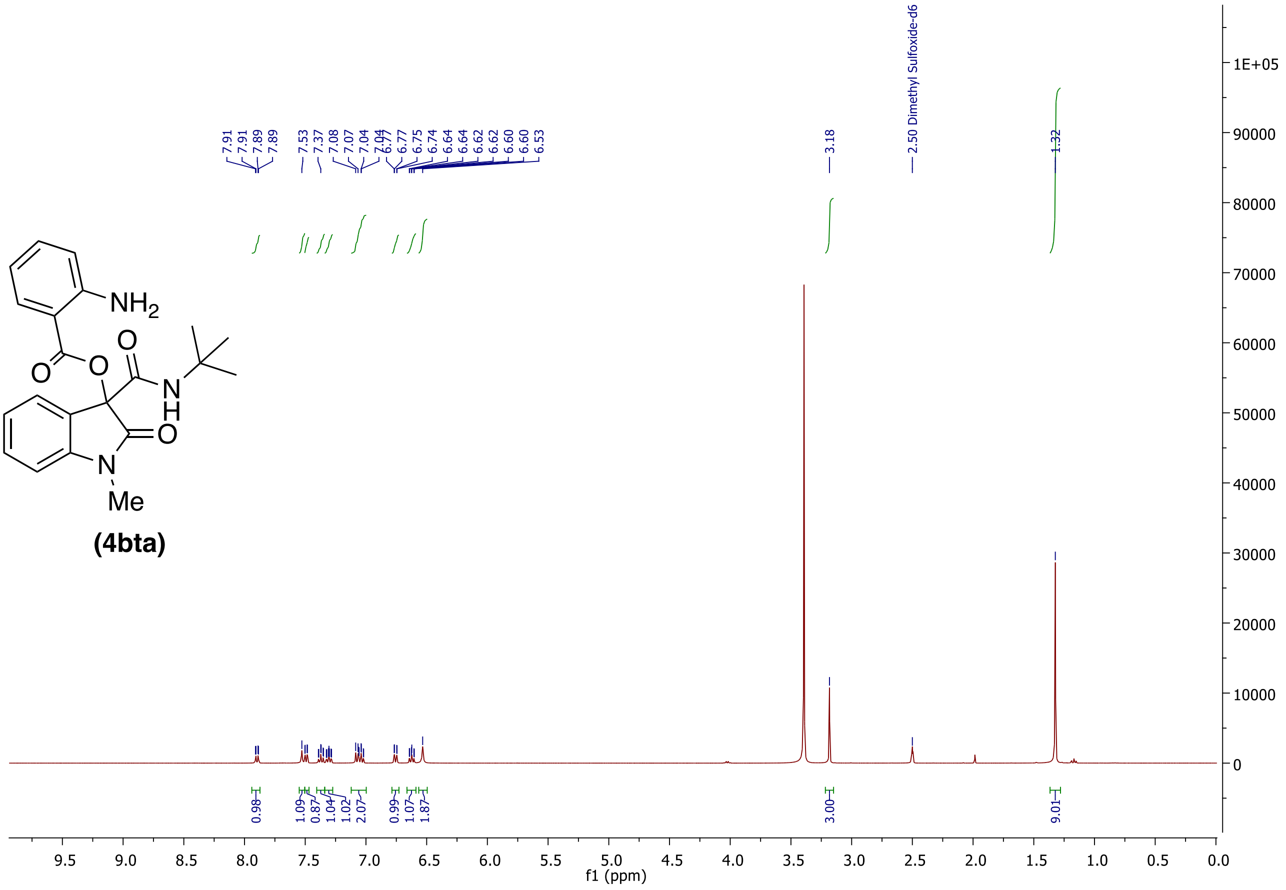

(4bta)

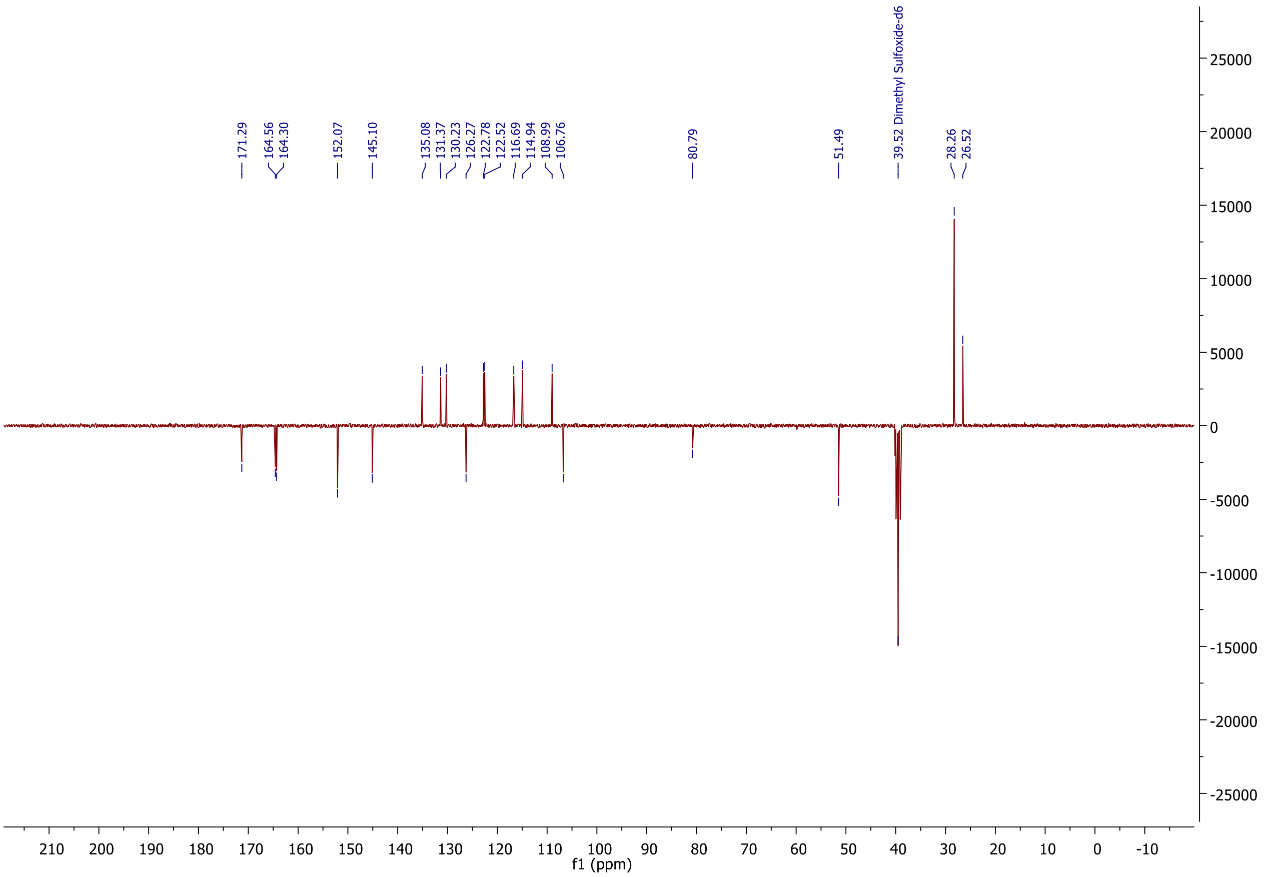

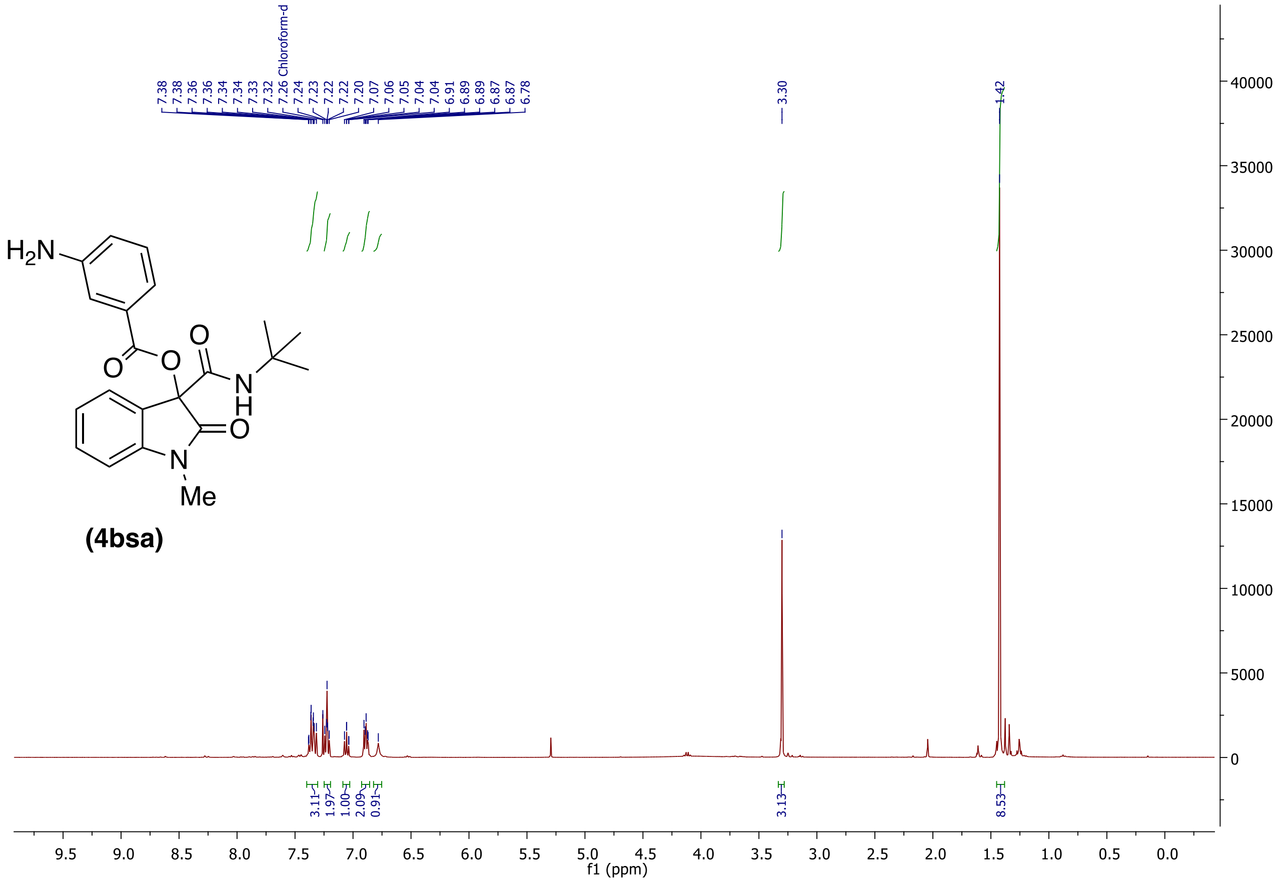

(4bsa)

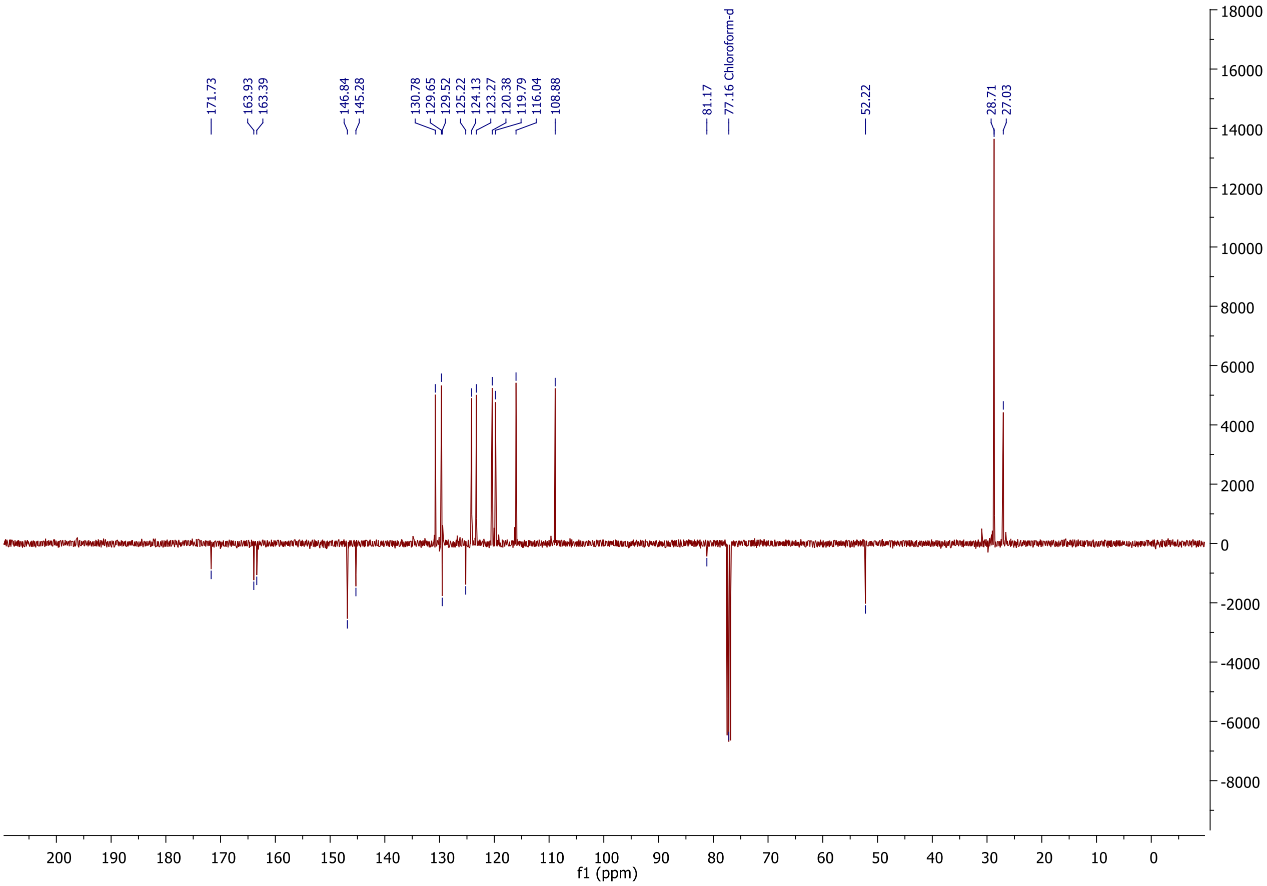

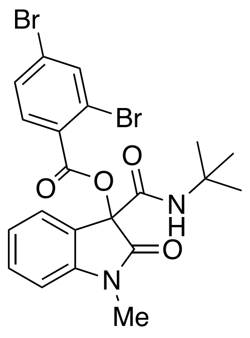
Br
Br
O
O
O
N
H
O
N
Me

(4bna)

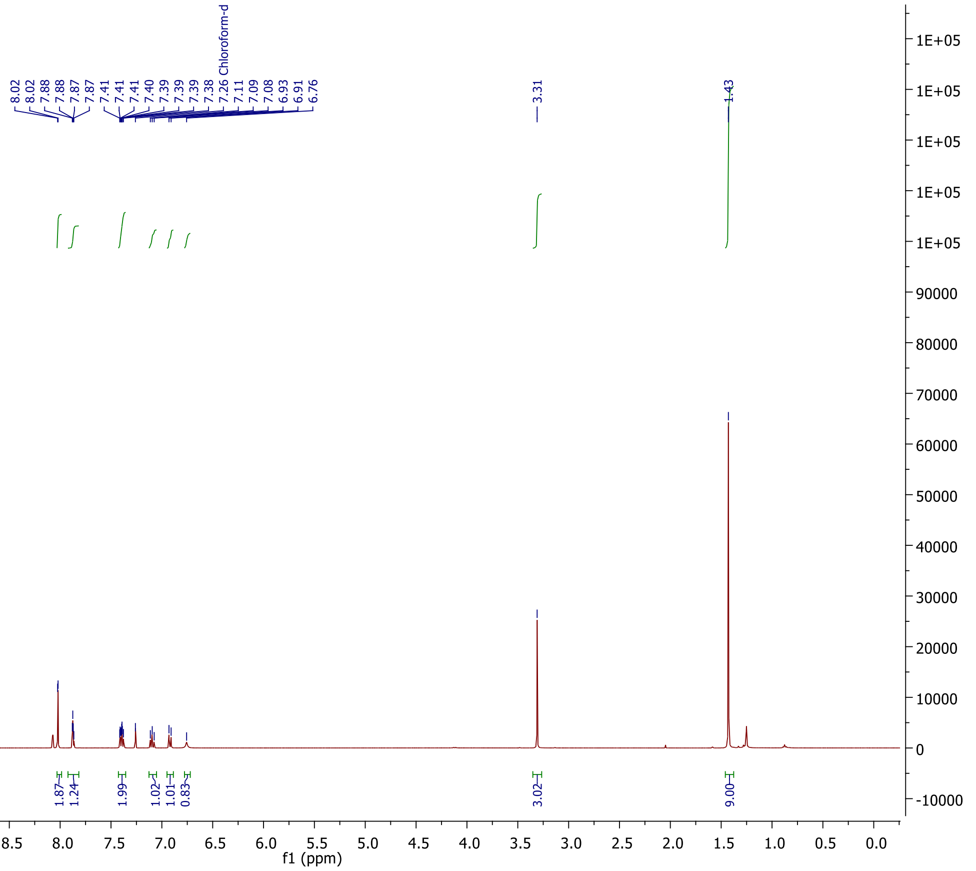
8.02
8.02
7.88
7.88
7.87
7.87
7.41
7.41
7.41
7.40
7.39
7.39
7.39
7.38
7.26 Chloroform-d
7.11
7.09
7.08
6.93
6.91
6.76
3.31
1.43
1.87
1.24
1.99
1.02
1.01
0.83
3.02
9.00
f1 (ppm)

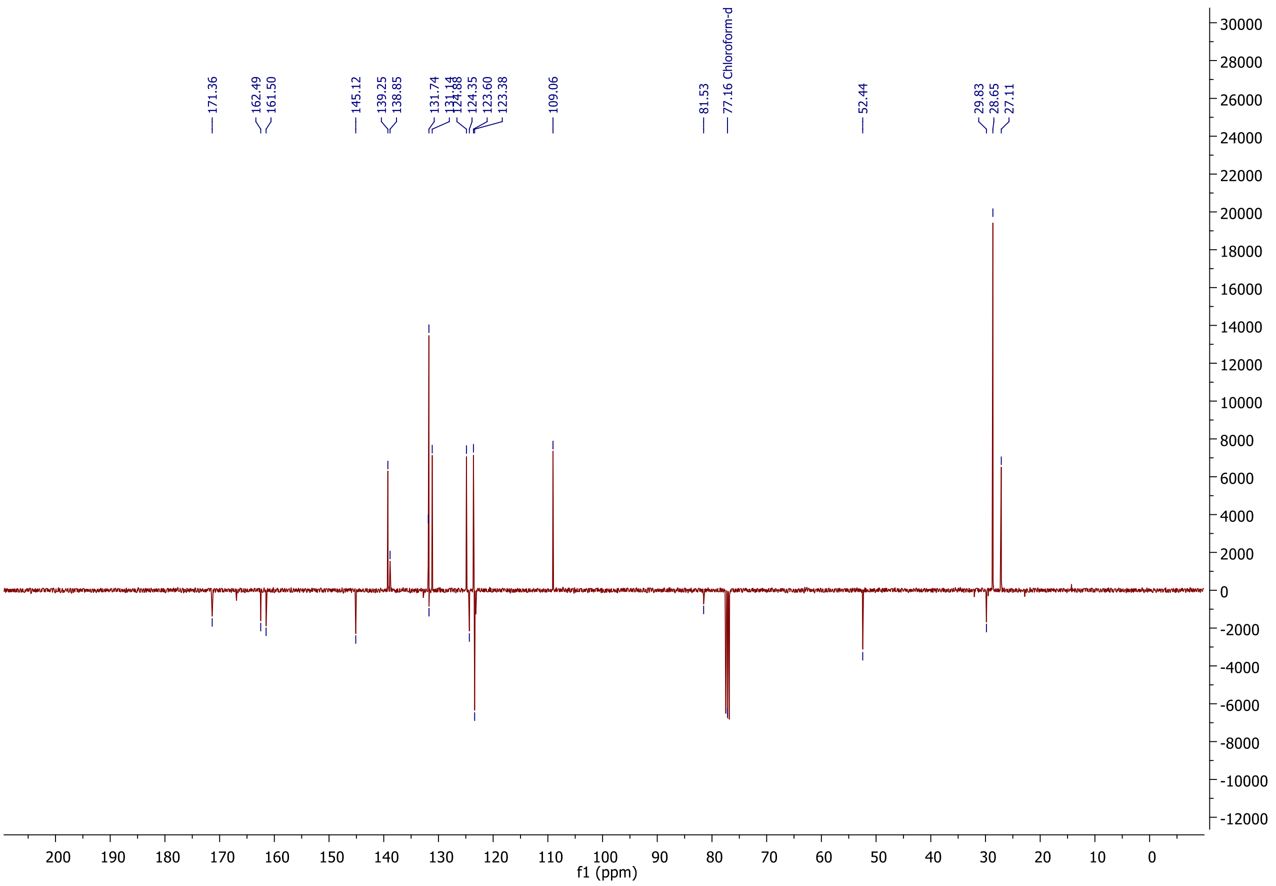
171.36
162.49
161.50
145.12
139.25
138.85
131.74
131.14
124.88
124.35
123.60
123.38
109.06
81.53
77.16 Chloroform-d
52.44
29.83
28.65
27.11
f1 (ppm)

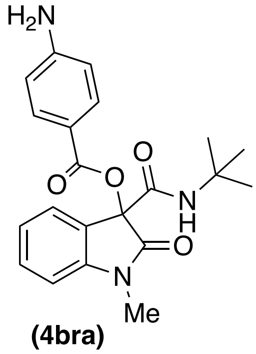
H2N
O
O
O
N
H
O
N
Me

(4bra)

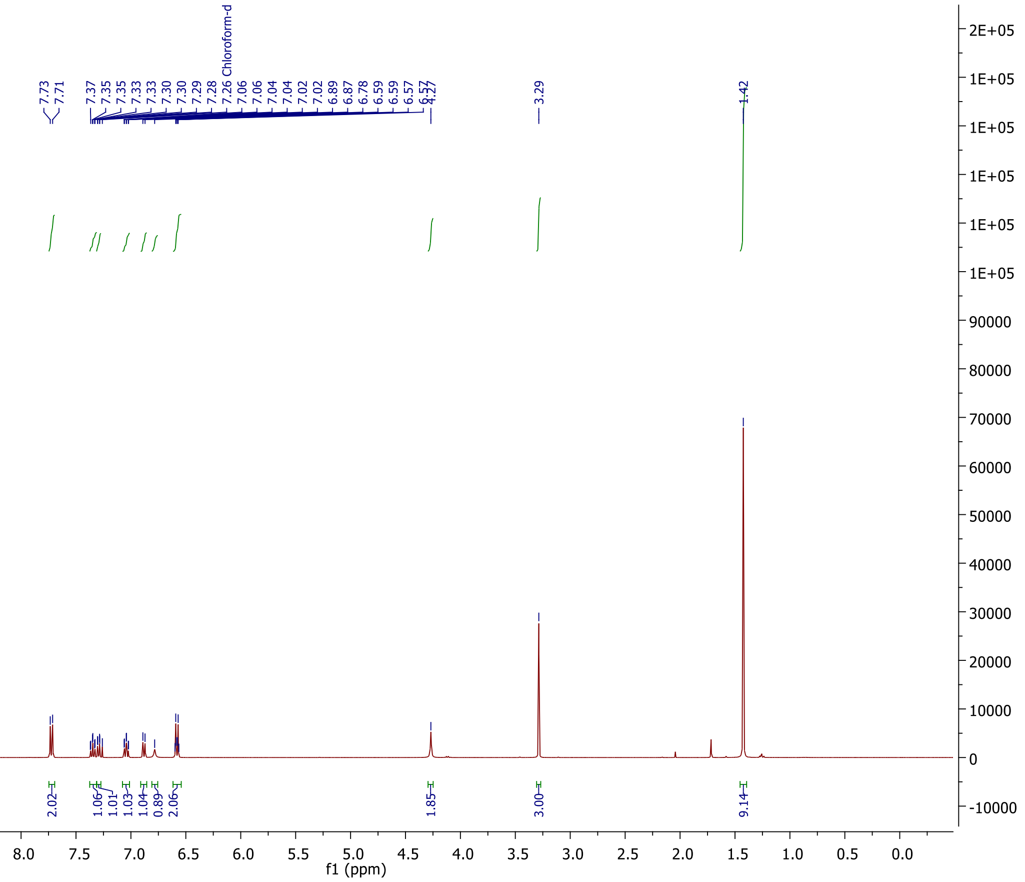
7.73
7.71
7.37
7.35
7.35
7.33
7.33
7.30
7.30
7.29
7.28
7.26 Chloroform-d
7.06
7.06
7.04
7.04
7.02
7.02
6.89
6.87
6.78
6.59
6.59
6.57
6.57
4.27
3.29
1.42
2.02
1.06
1.01
1.03
1.04
0.89
2.06
1.85
3.00
9.14
f1 (ppm)

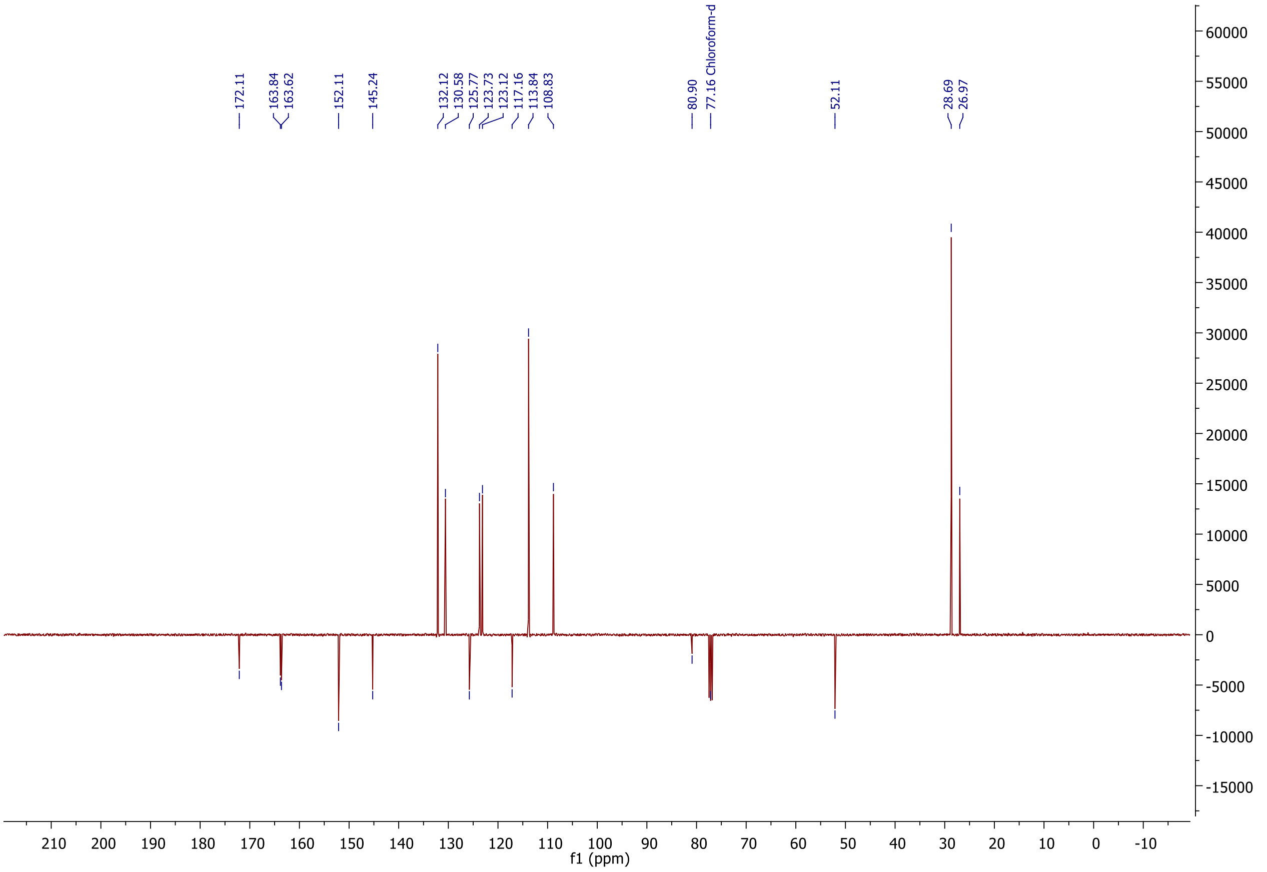
172.11
163.84
163.62
152.11
145.24
132.12
130.58
125.77
123.73
123.12
117.16
113.84
108.83
80.90
77.16 Chloroform-d
52.11
28.69
26.97
f1 (ppm)

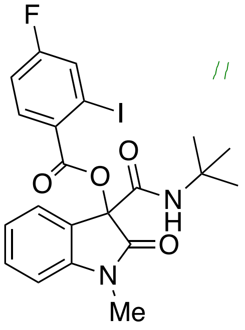
F
I
O
O
O
N
H
O
N
Me

(4bfa)

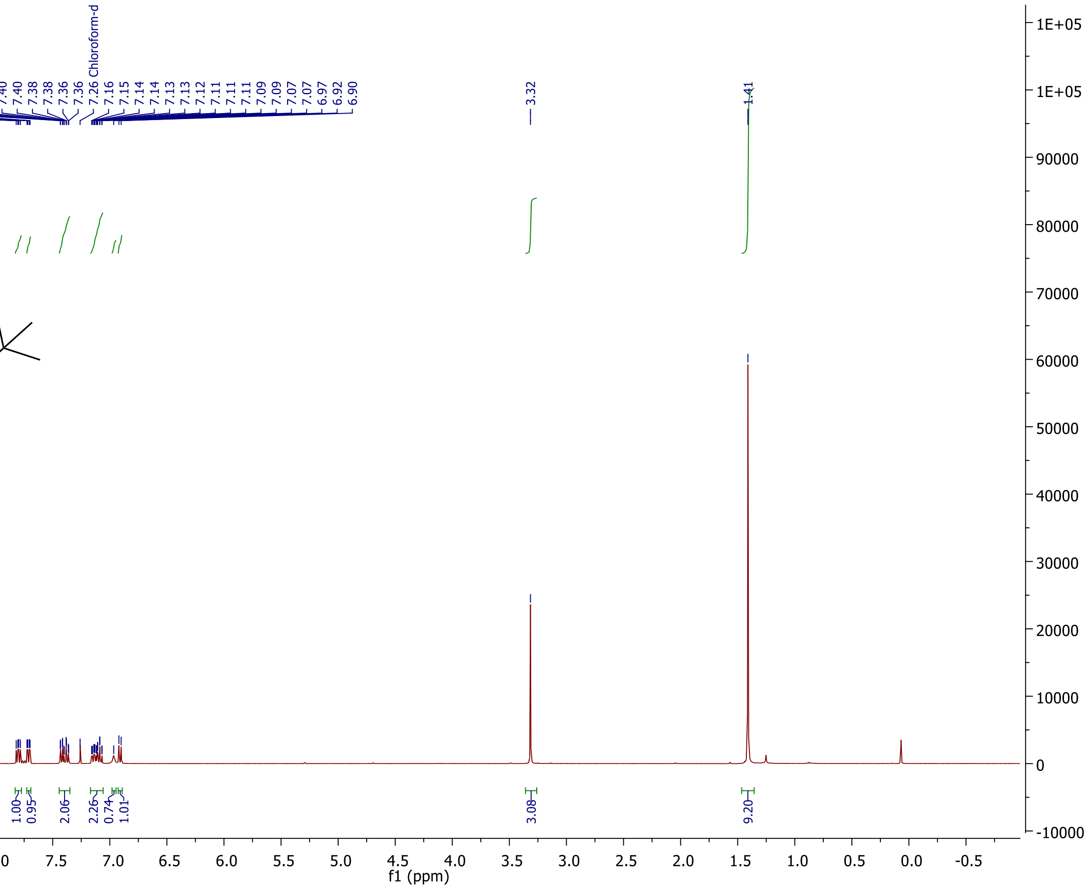
7.82
7.81
7.80
7.78
7.72
7.72
7.70
7.70
7.43
7.43
7.41
7.41
7.40
7.40
7.38
7.38
7.36
7.36
7.26 Chloroform-d
7.16
7.15
7.14
7.14
7.13
7.13
7.12
7.11
7.11
7.11
7.09
7.09
7.07
7.07
6.97
6.92
6.90
3.32
1.41
1.00
0.95
2.06
2.26
0.74
1.01
3.08
9.20
f1 (ppm)

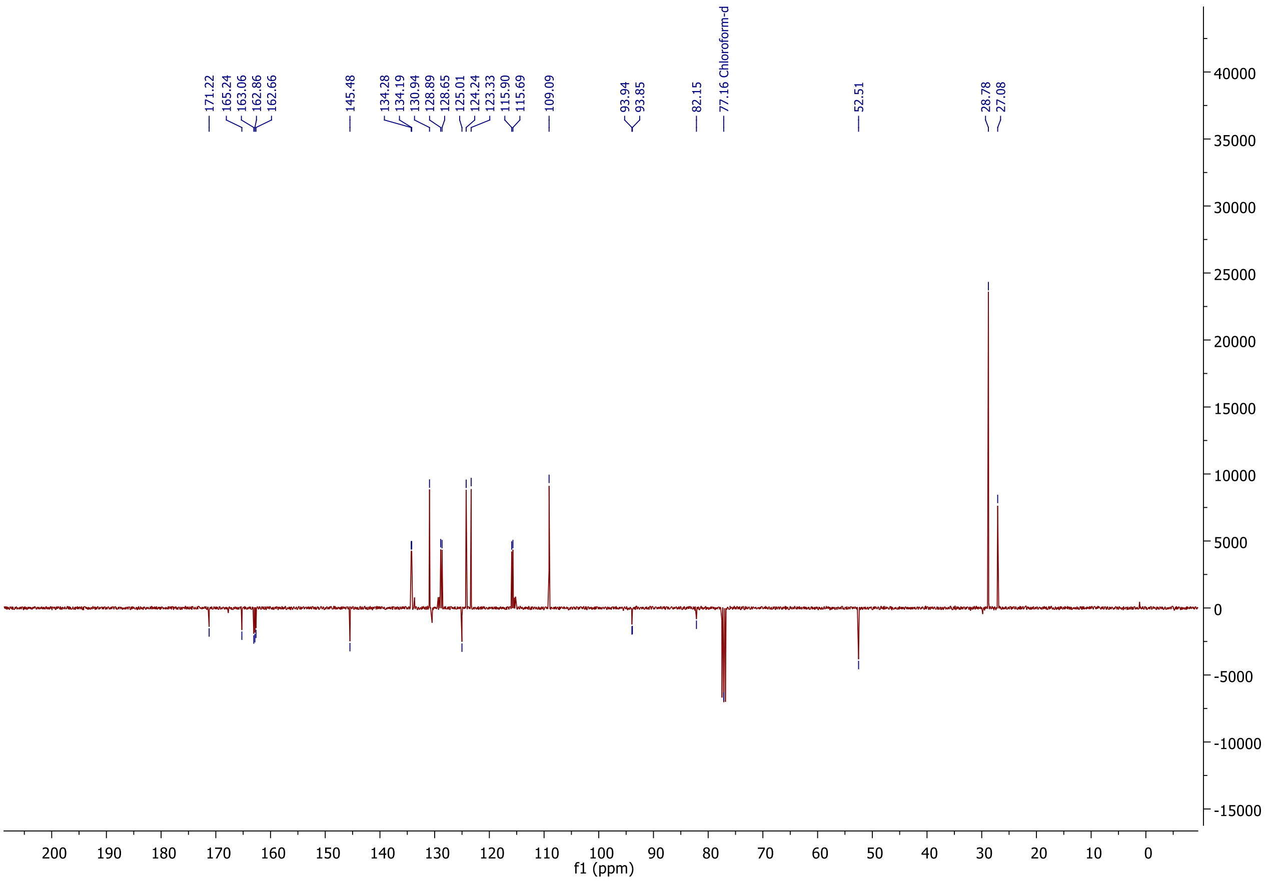
171.22
165.24
163.06
162.86
162.66
145.48
134.28
134.19
130.94
128.89
128.65
125.01
124.24
123.33
115.90
115.69
109.09
93.94
93.85
82.15
77.16 Chloroform-d
52.51
28.78
27.08
f1 (ppm)

^{1}H and ^{13}C NMR spectra

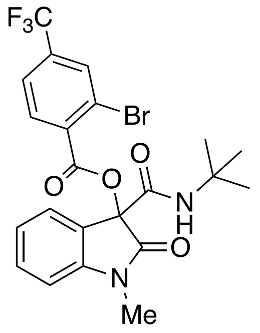

(4bga)

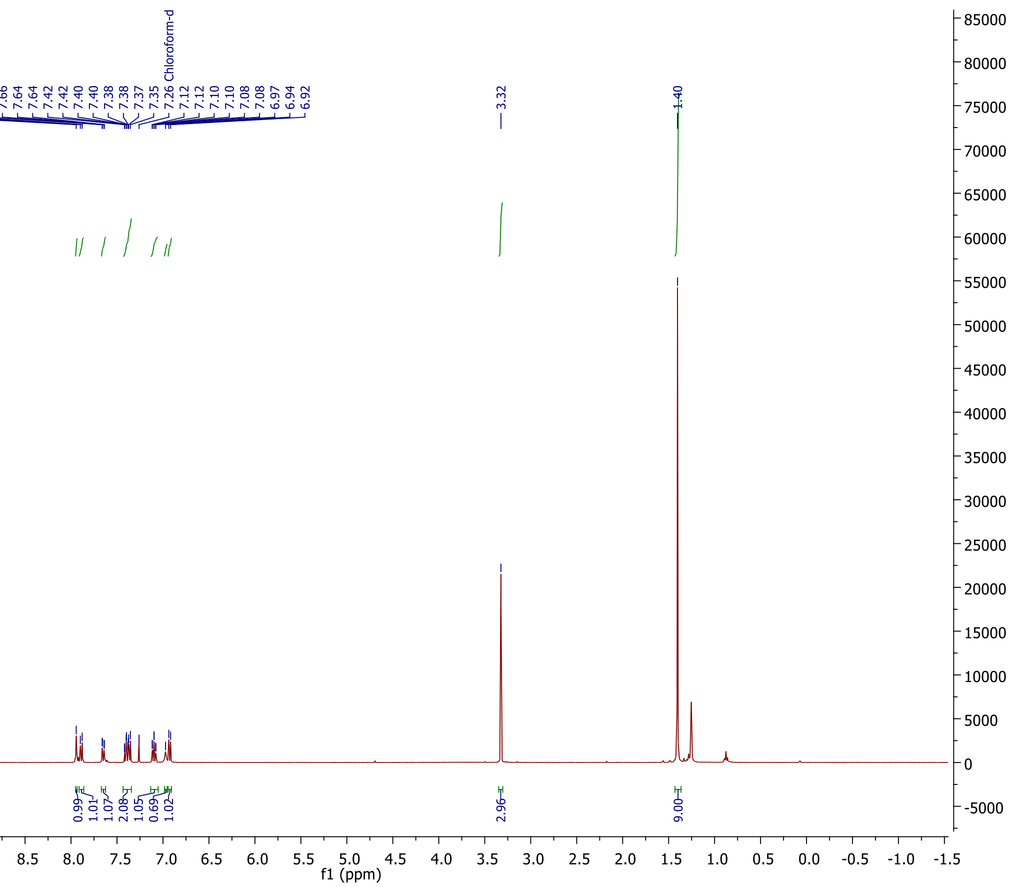

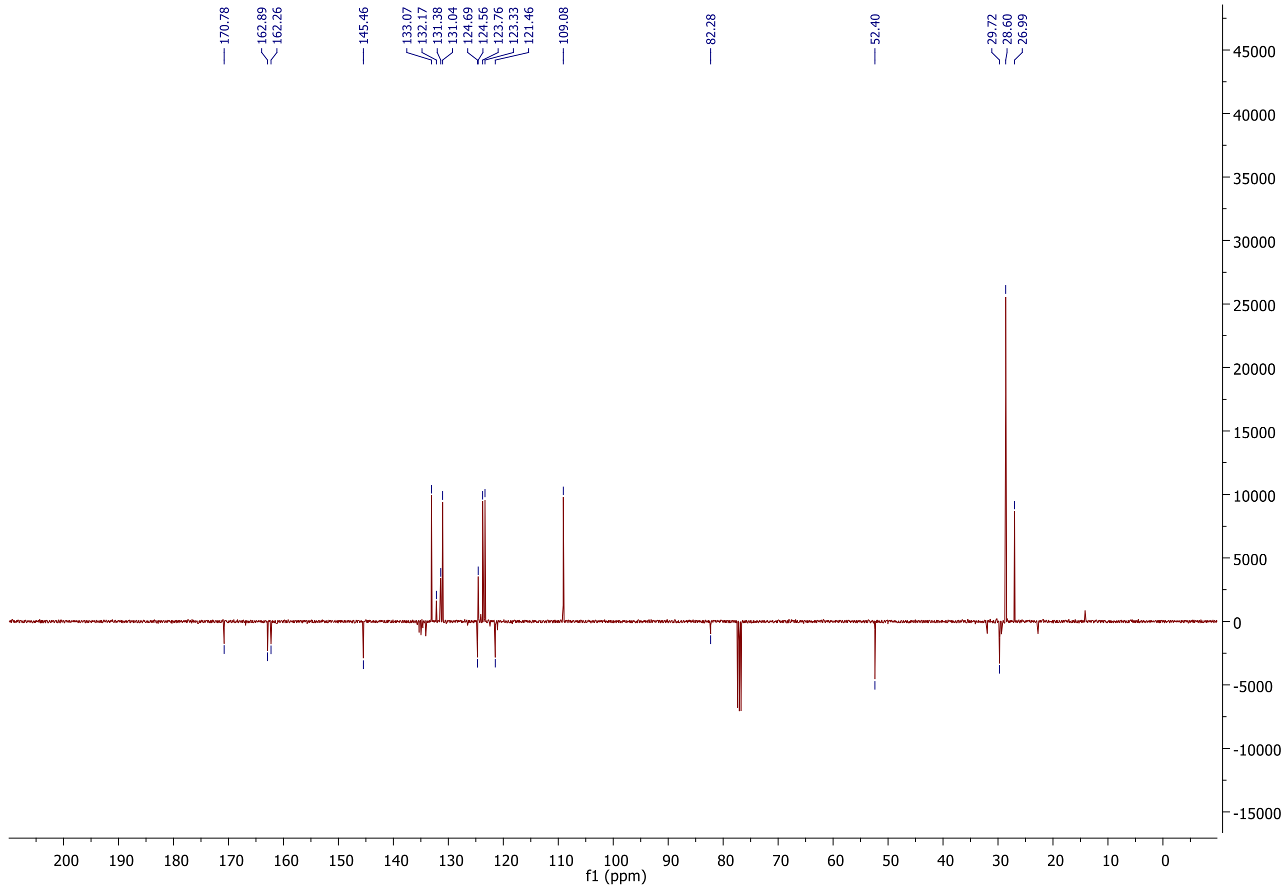

^{1}H and ^{13}C NMR spectra

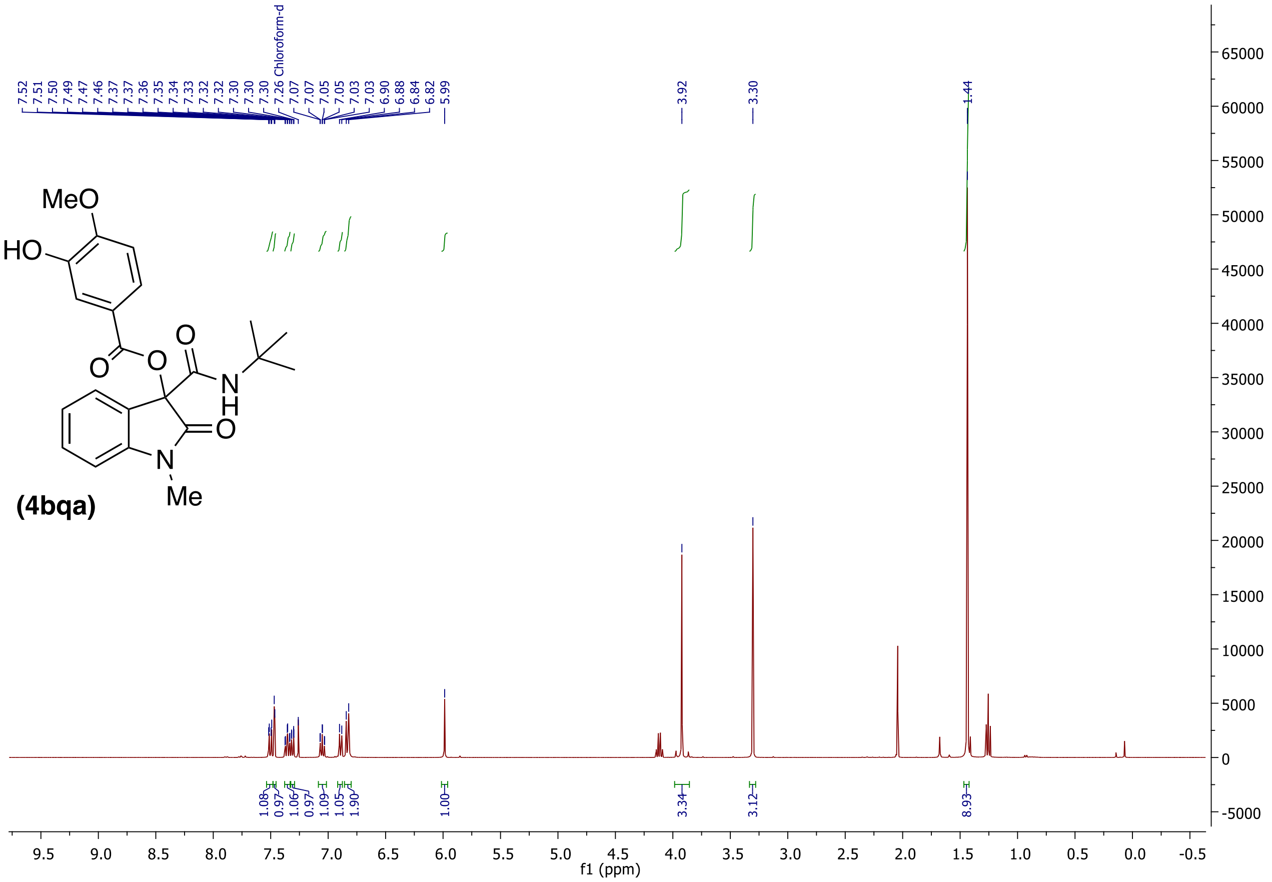

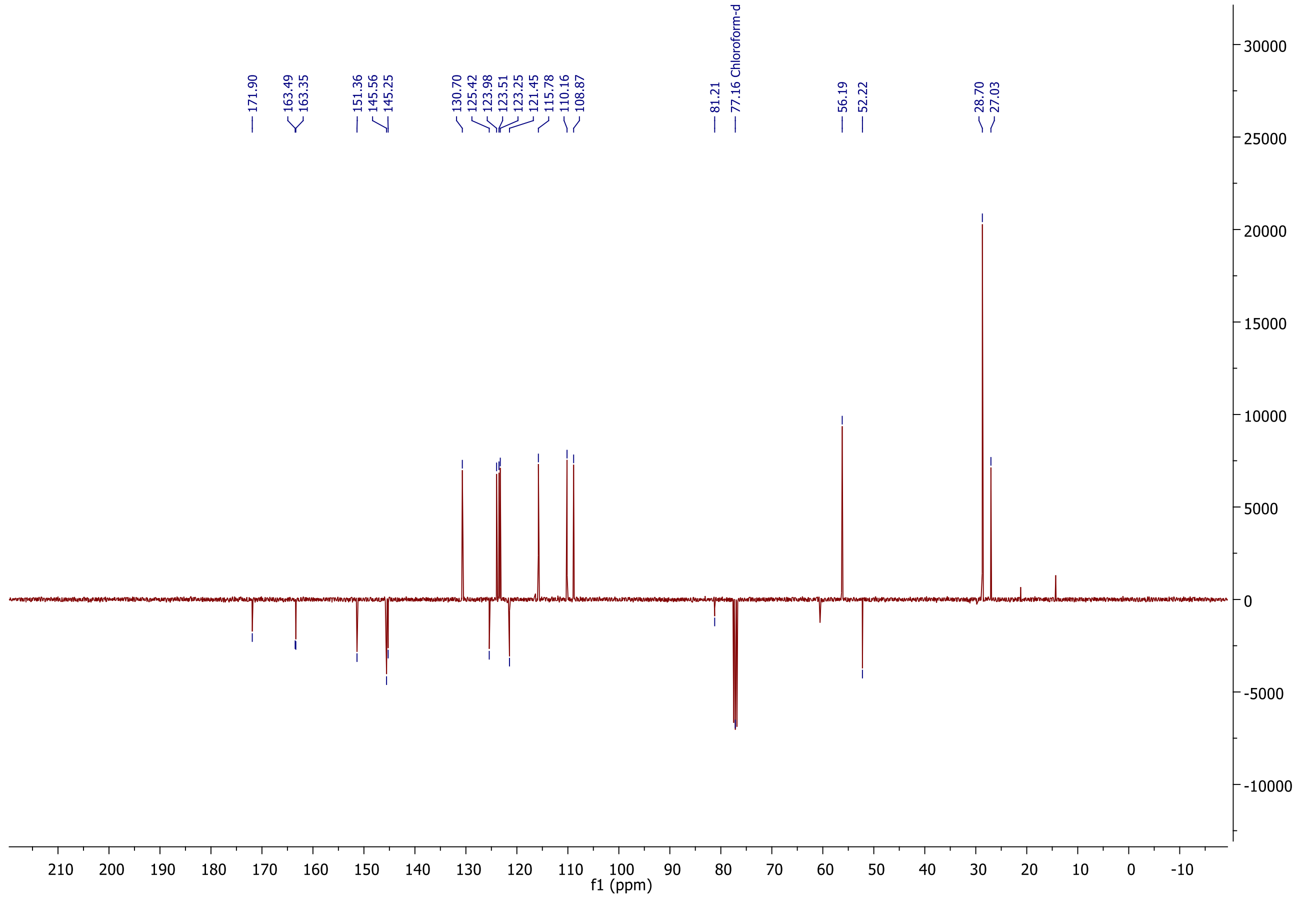

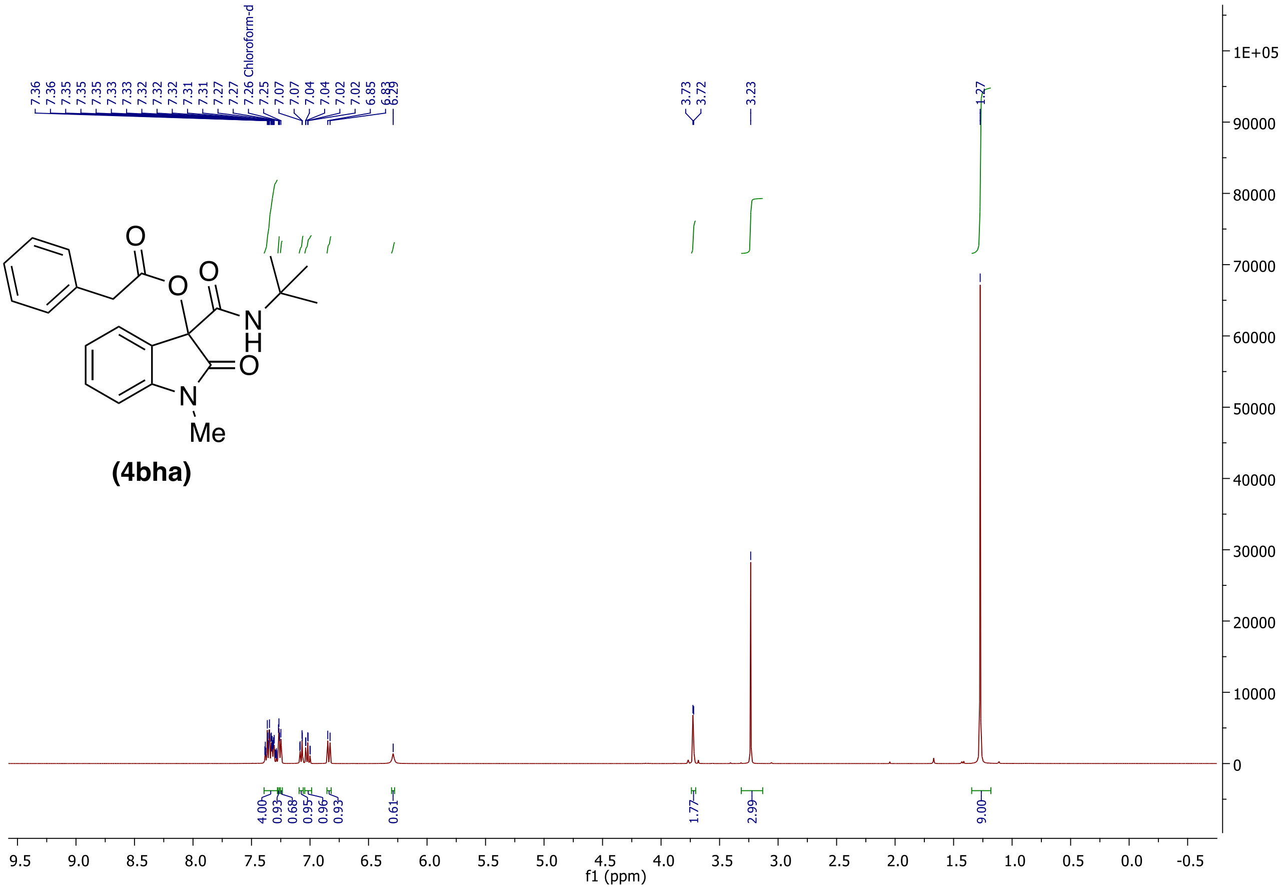

(4bha)

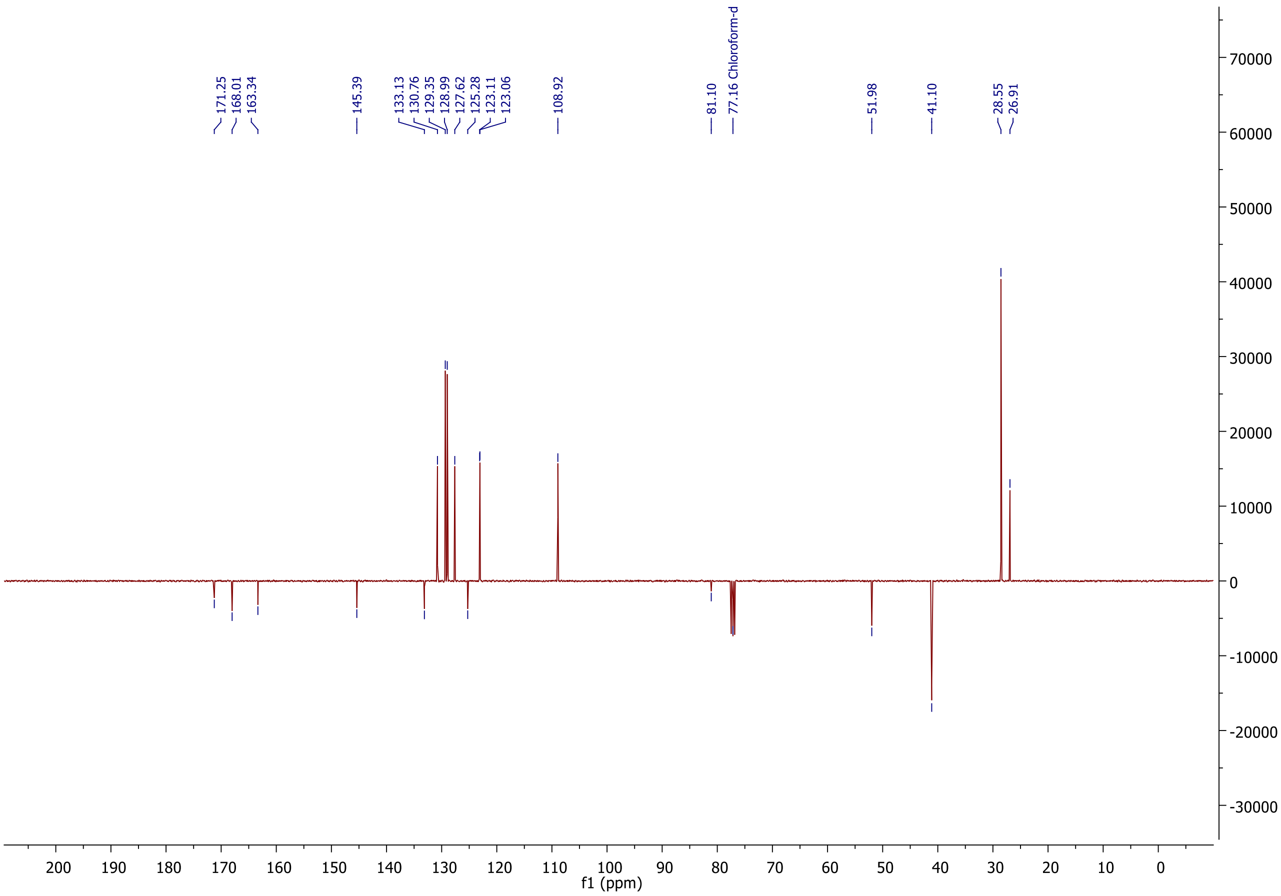

(4bia)

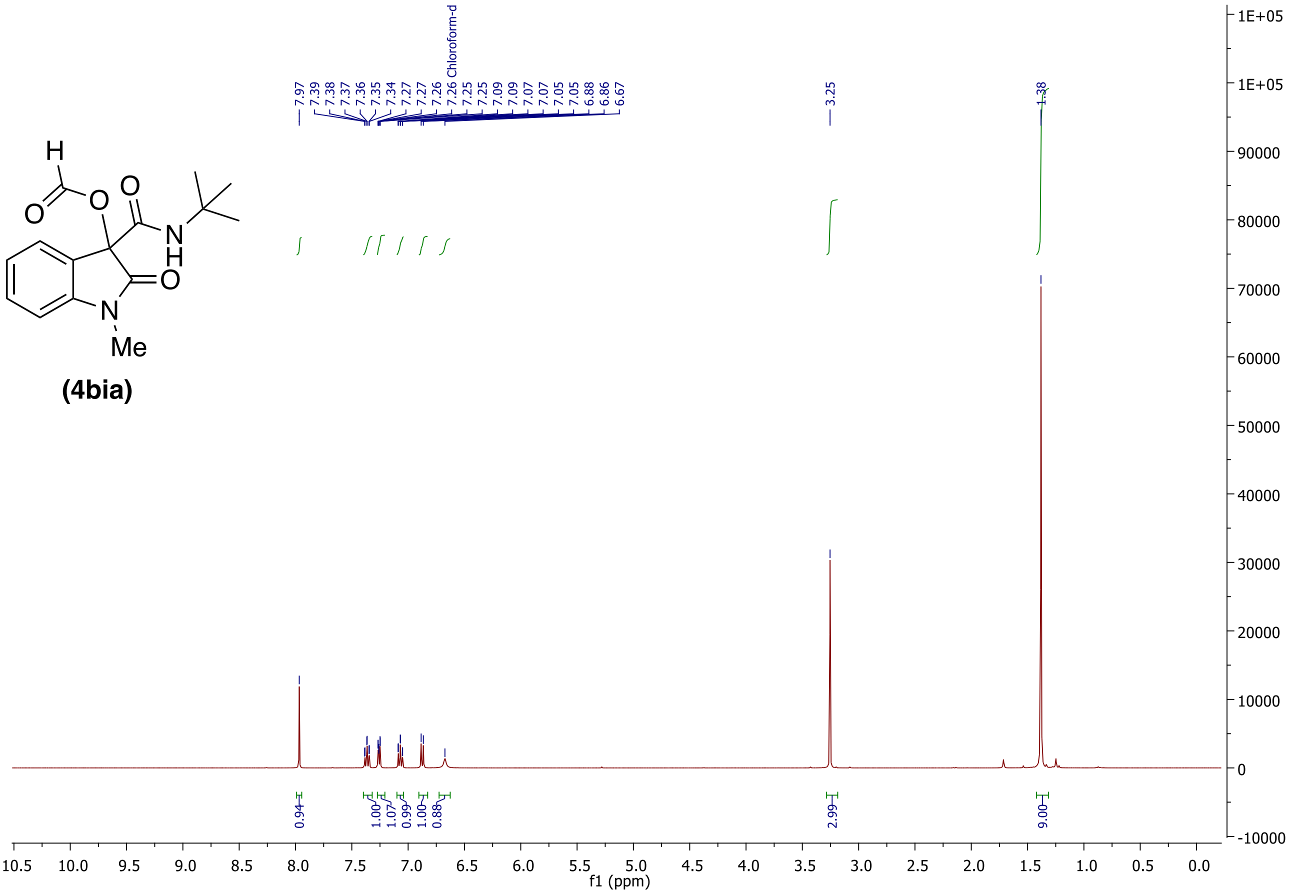
7.97
7.39
7.38
7.37
7.36
7.35
7.34
7.27
7.27
7.26 Chloroform-d
7.25
7.25
7.09
7.09
7.07
7.07
7.05
7.05
6.88
6.86
6.67
3.25
1.38
0.94
1.00
1.07
0.99
1.00
0.88
2.99
9.00
f1 (ppm)

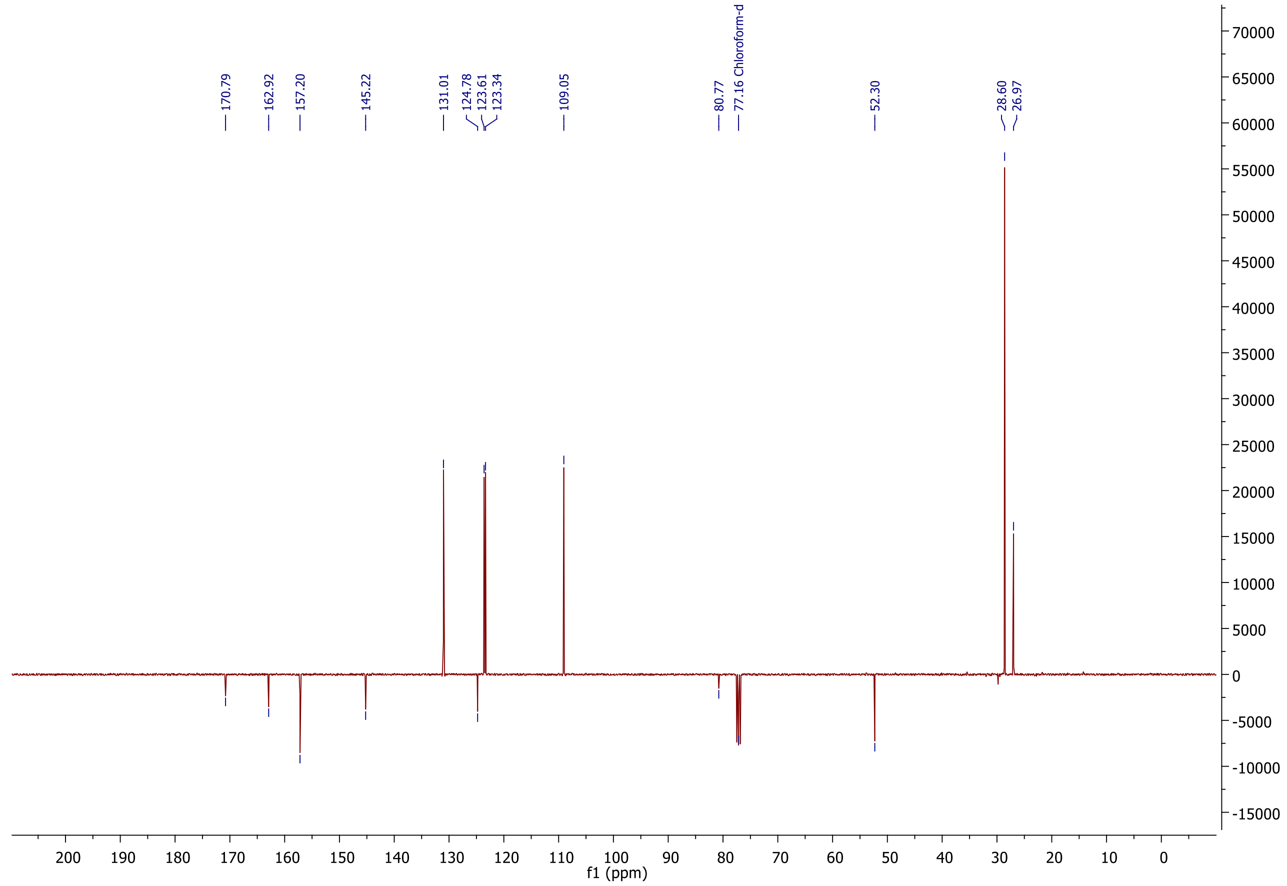
170.79
162.92
157.20
145.22
131.01
124.78
123.61
123.34
109.05
80.77
77.16 Chloroform-d
52.30
28.60
26.97
f1 (ppm)

(4bja)

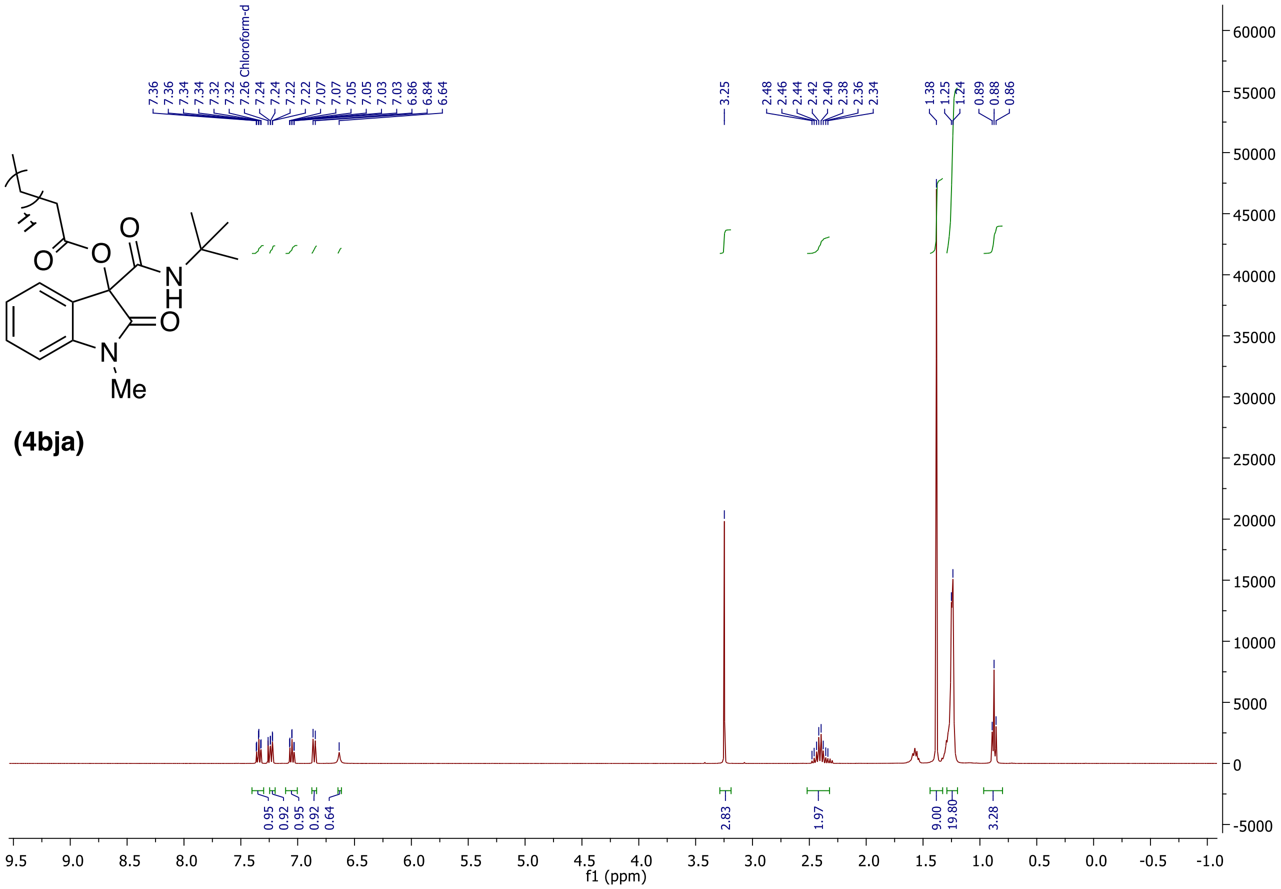
7.36
7.36
7.34
7.34
7.32
7.32
7.26 Chloroform-d
7.24
7.24
7.22
7.22
7.07
7.05
7.05
7.03
7.03
6.86
6.84
6.64
3.25
2.48
2.46
2.44
2.42
2.40
2.38
2.36
2.34
1.38
1.25
1.24
0.89
0.88
0.86
0.95
0.92
0.95
0.92
0.64
2.83
1.97
9.00
19.80
3.28
f1 (ppm)

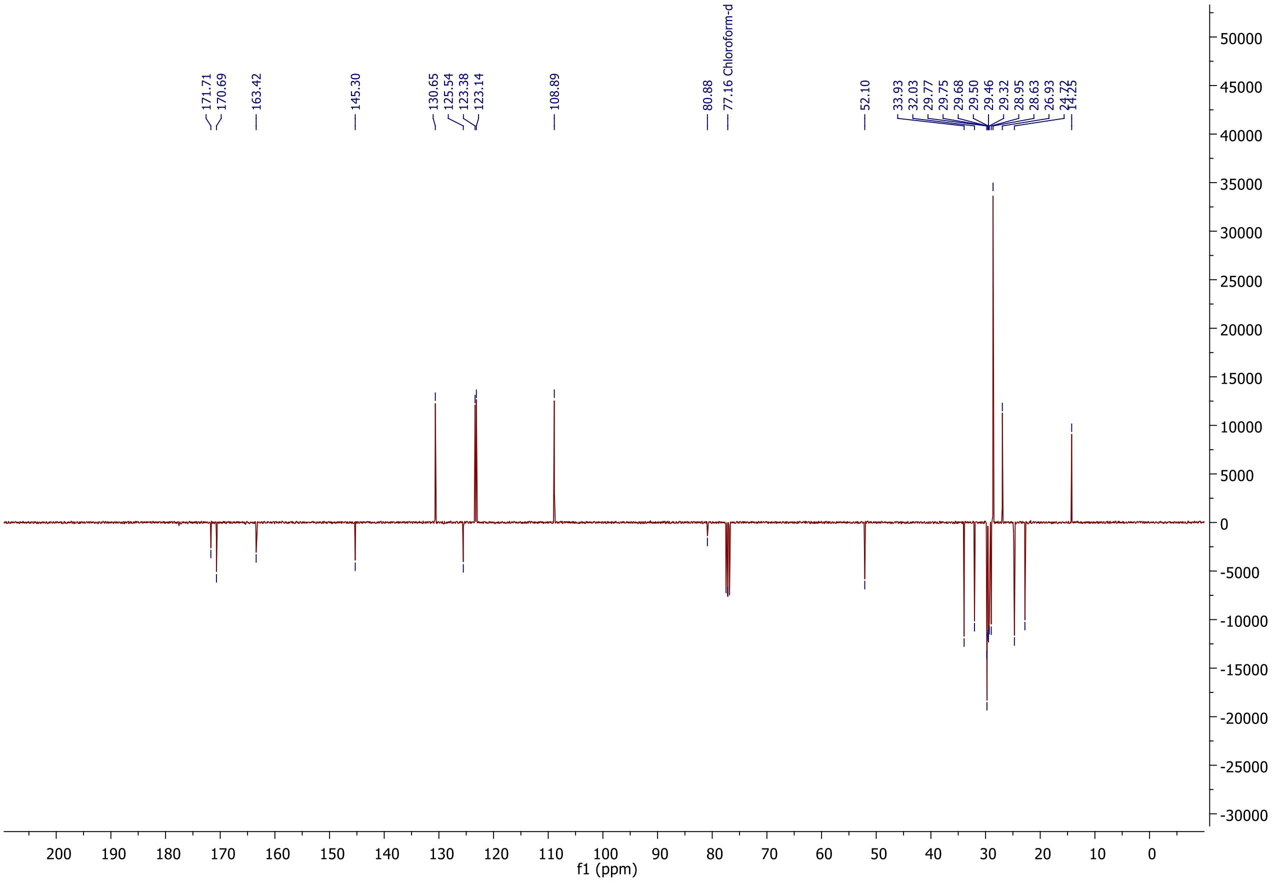
171.71
170.69
163.42
145.30
130.65
125.54
123.38
123.14
108.89
80.88
77.16 Chloroform-d
52.10
33.93
32.03
29.77
29.75
29.68
29.50
29.46
29.32
28.95
28.63
26.93
24.72
14.25
f1 (ppm)

^1H and ^{13}C NMR spectra

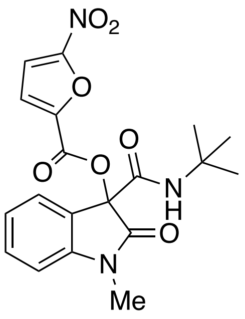

(4bma)

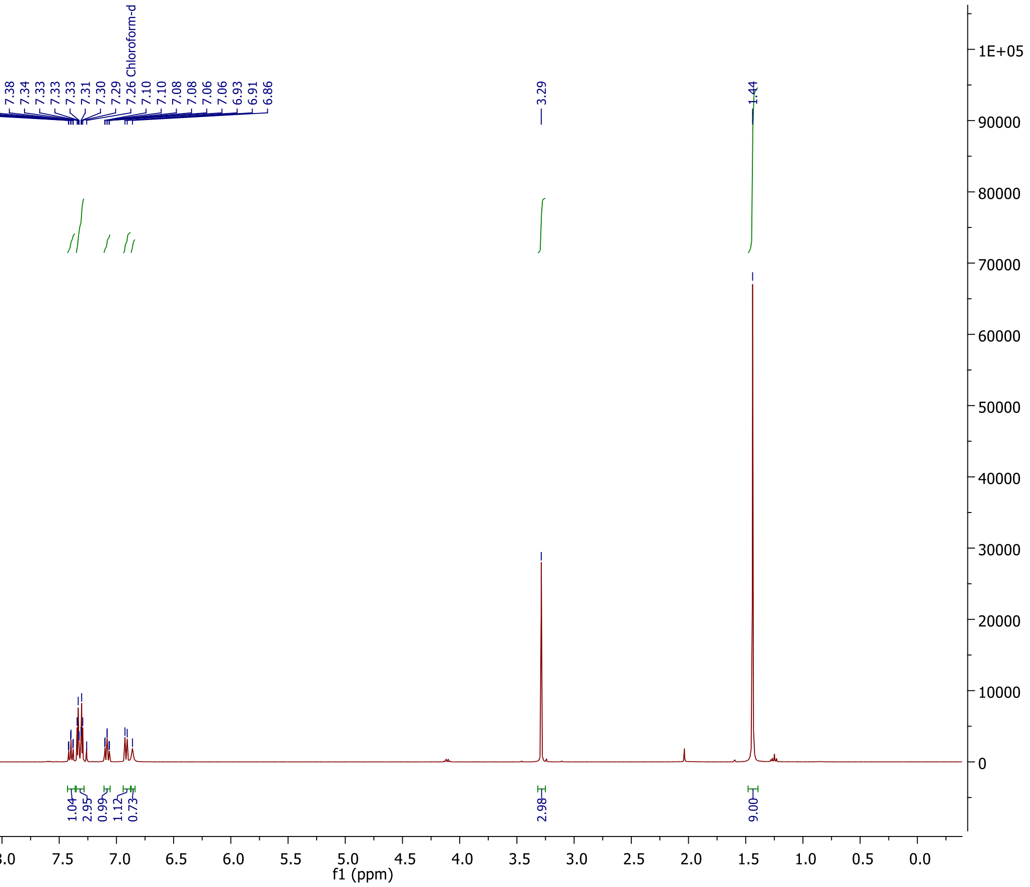

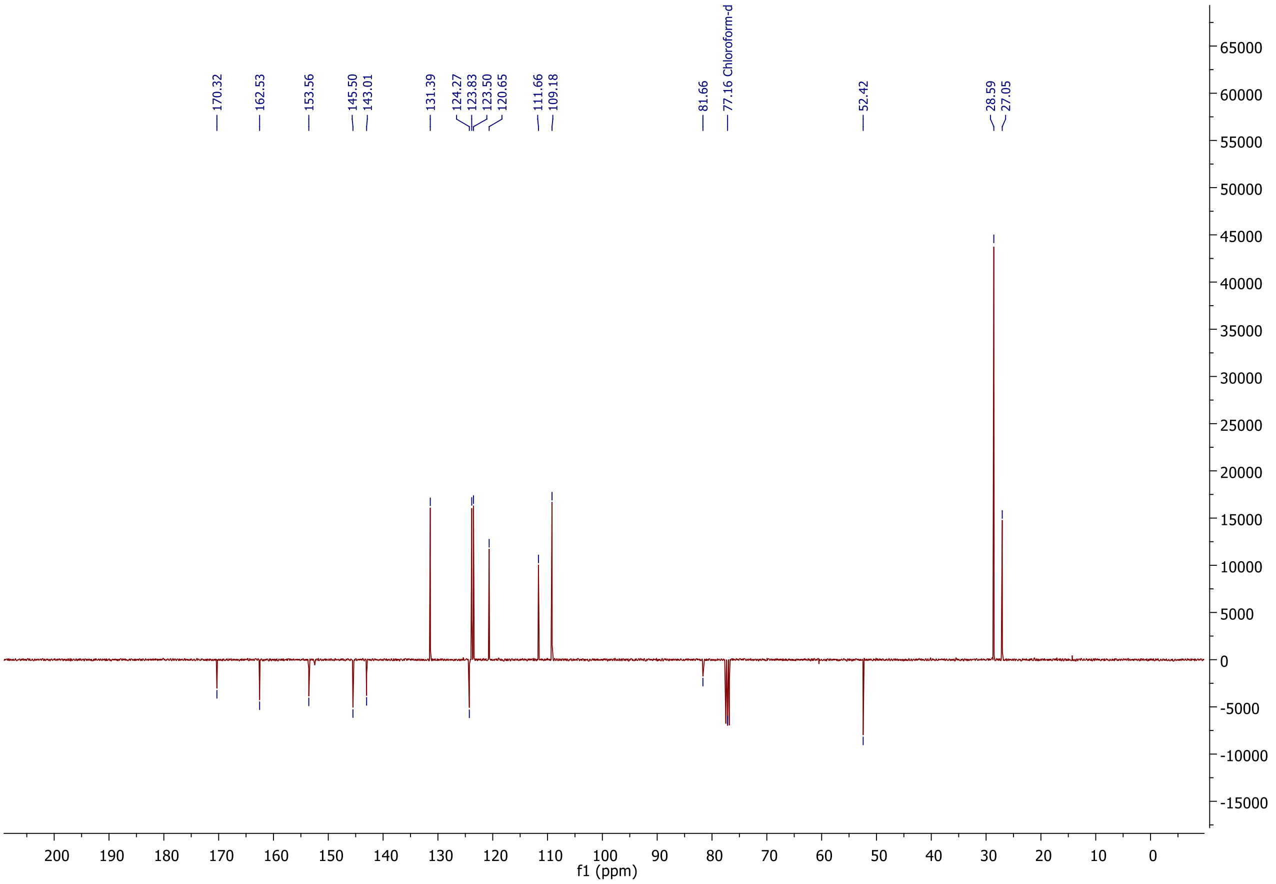

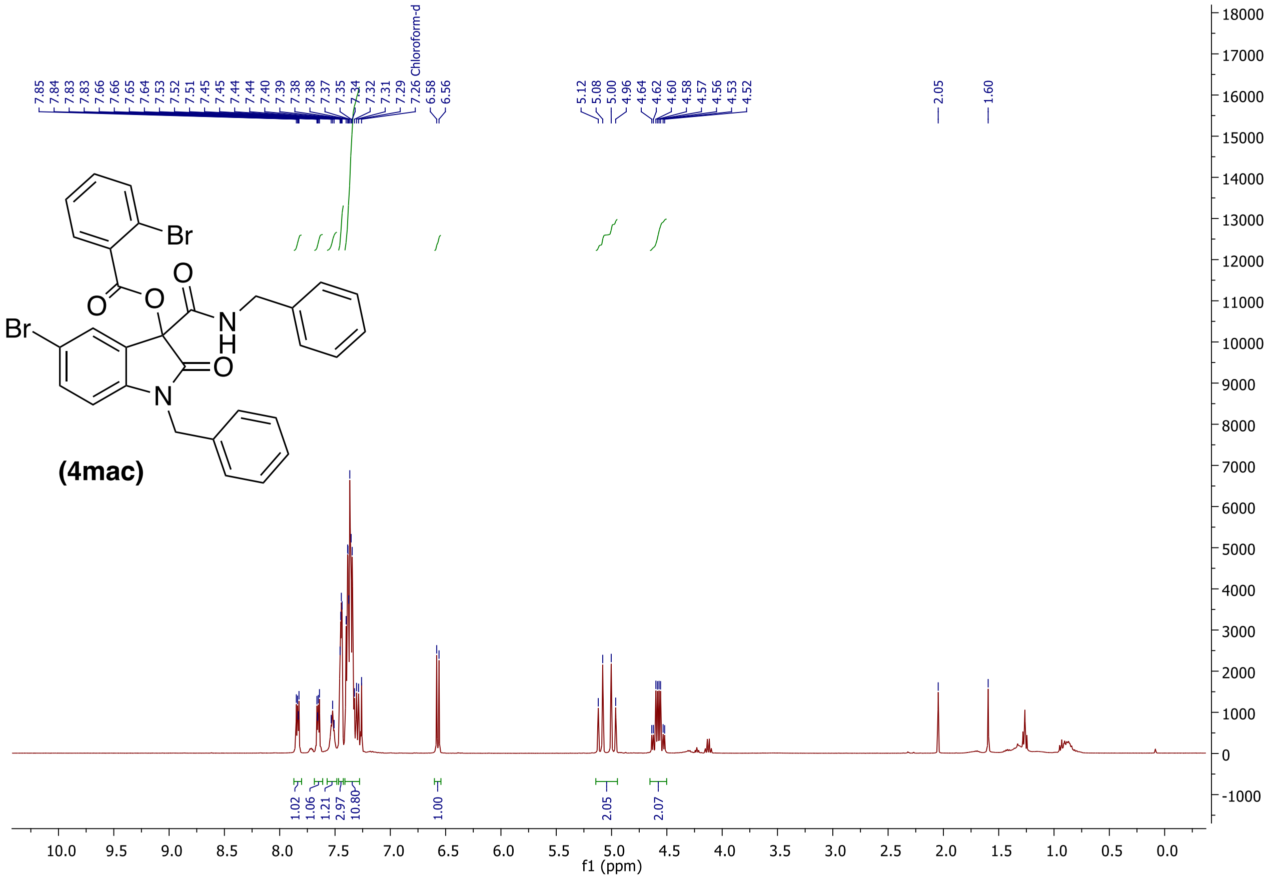
Br
O
O
O
Br
N
H
O
N
(4mac)

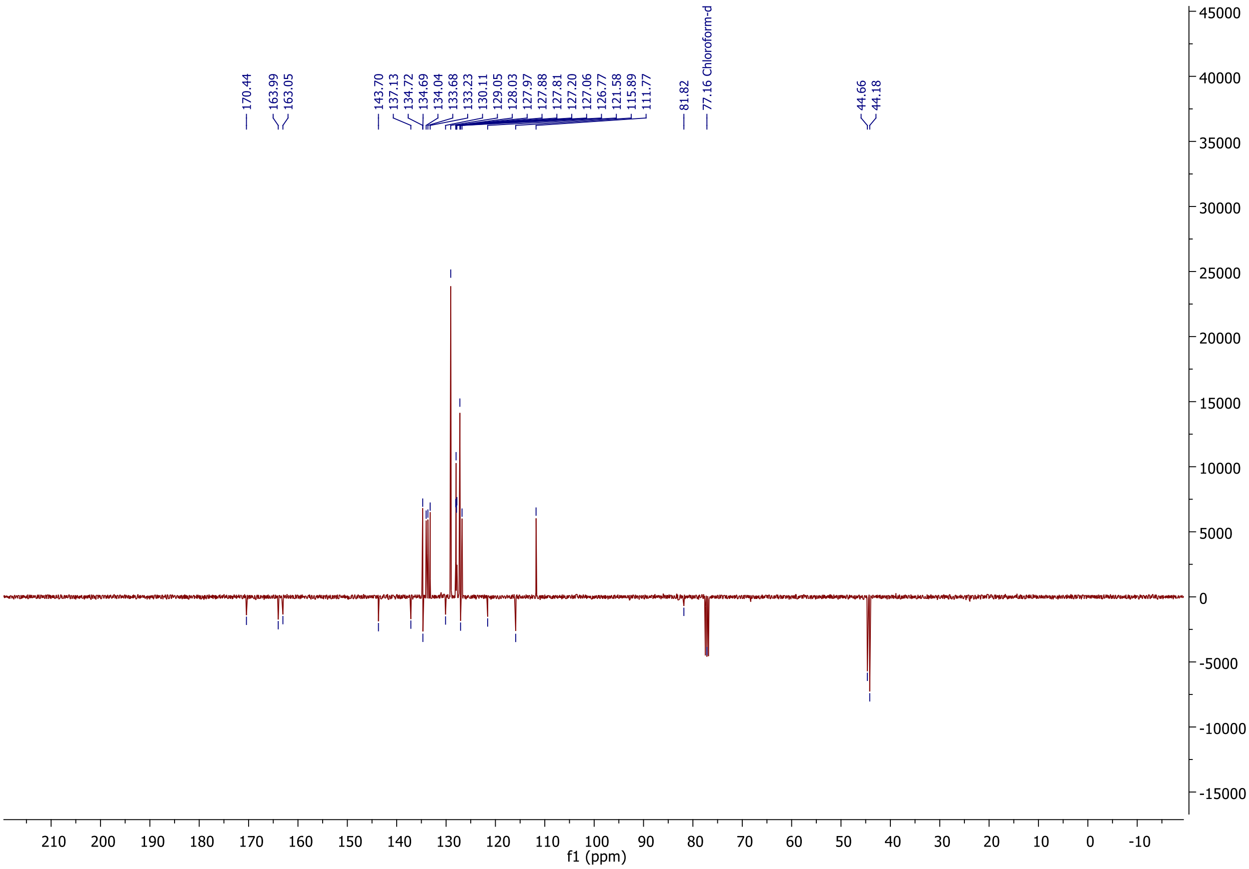

^{1}H and ^{13}C NMR spectra

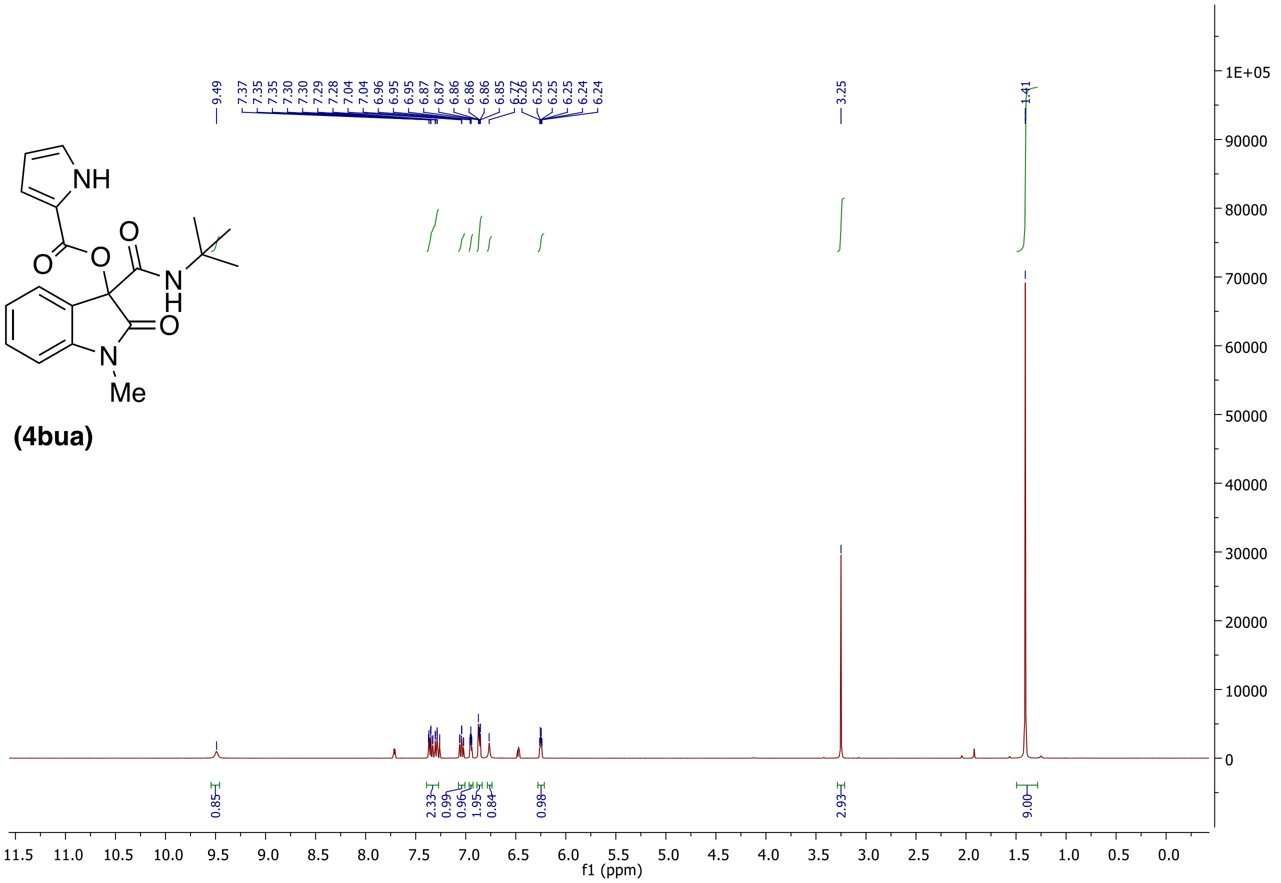

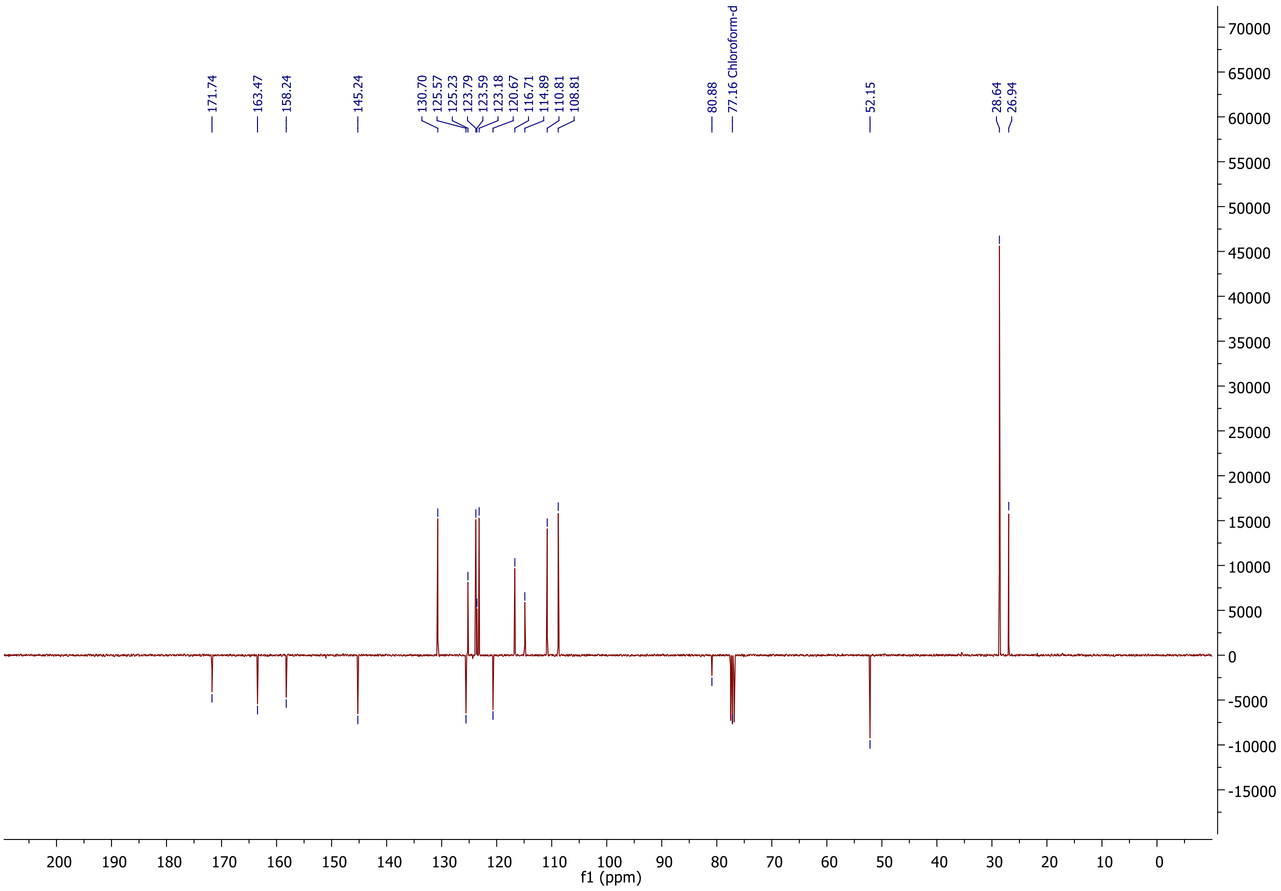

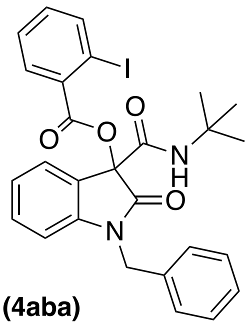

(4aba)

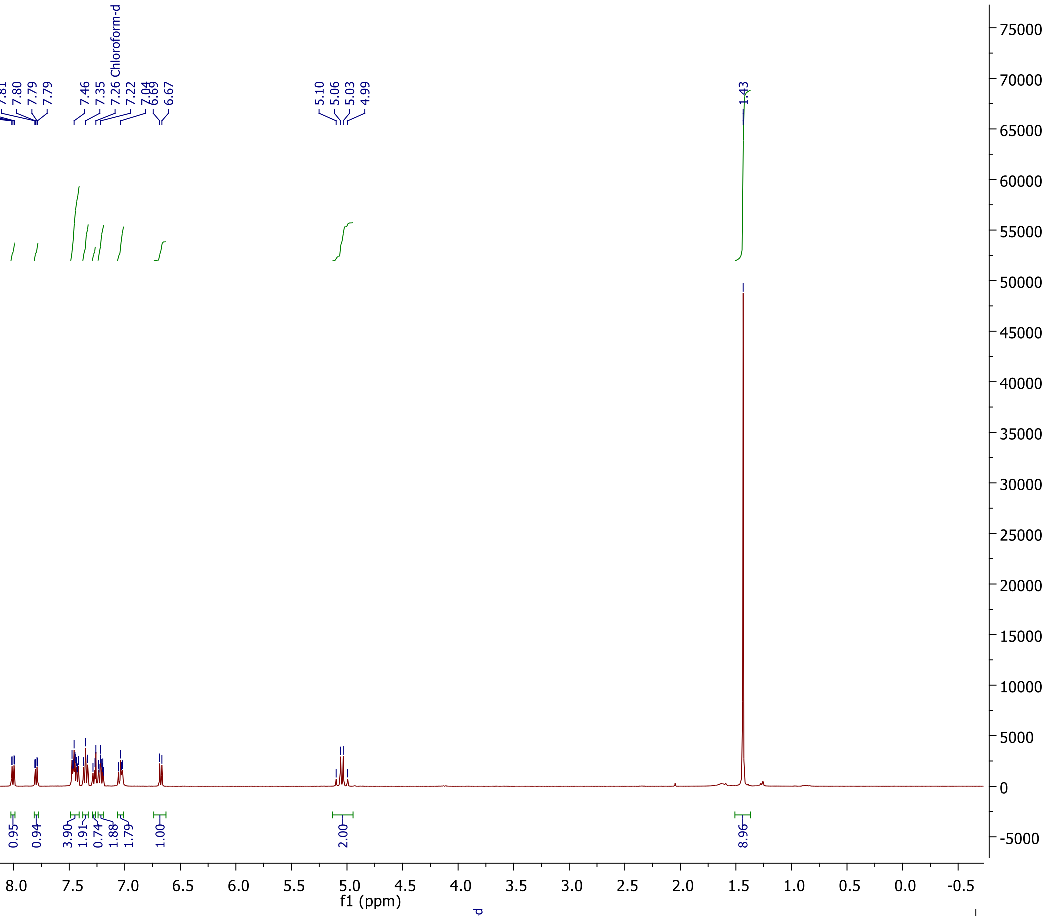

8.02
8.01
8.00
7.99
7.81
7.80
7.79
7.79
7.46
7.35
7.26 Chloroform-d
7.22
7.04
6.69
6.67
5.10
5.06
5.03
4.99
1.43
0.95
0.94
3.90
1.91
0.74
1.88
1.79
1.00
2.00
8.96
f1 (ppm)

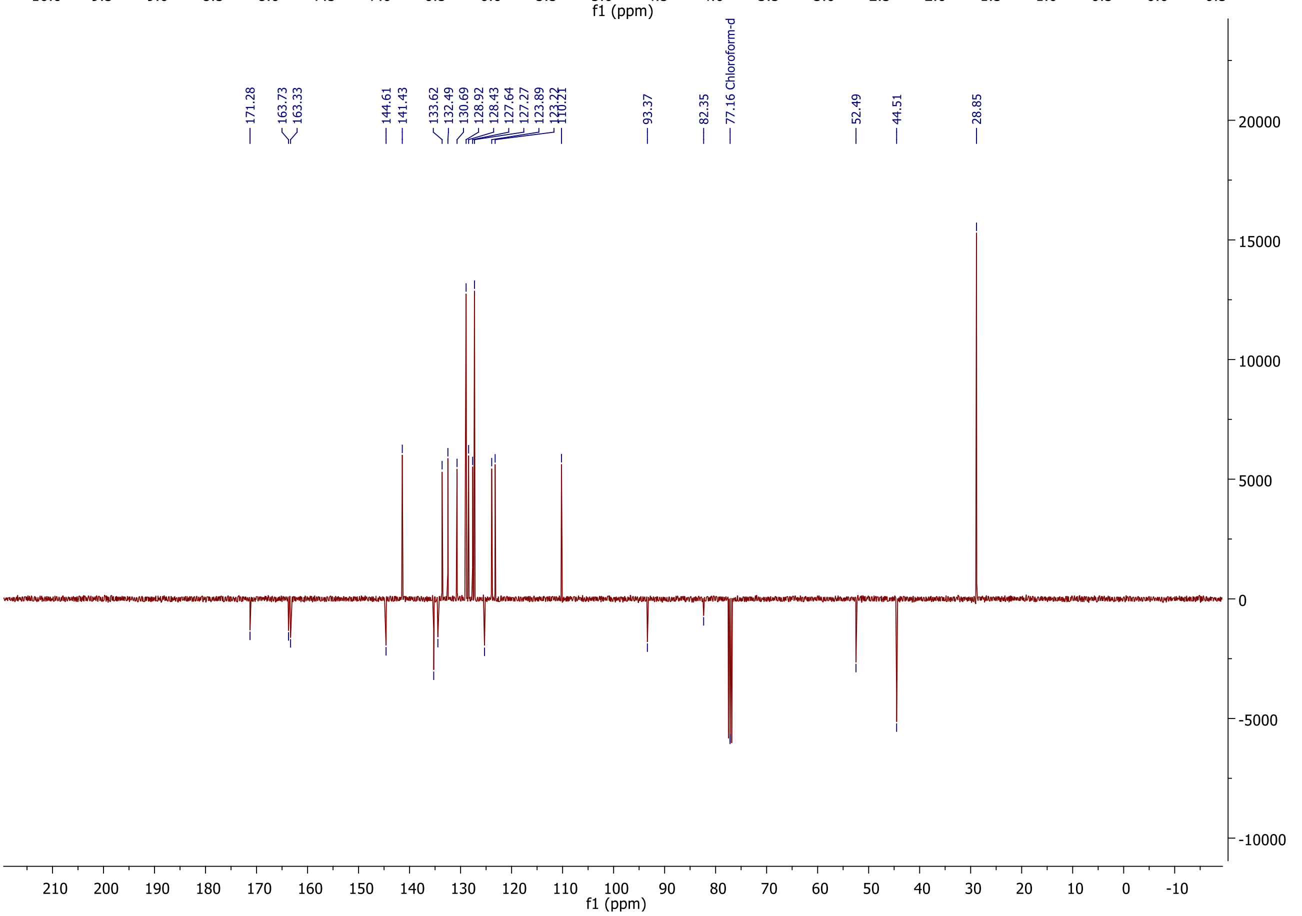

171.28
163.73
163.33
144.61
141.43
133.62
132.49
130.69
128.92
128.43
127.64
127.27
123.89
123.22
110.21
93.37
82.35
77.16 Chloroform-d
52.49
44.51
28.85
f1 (ppm)

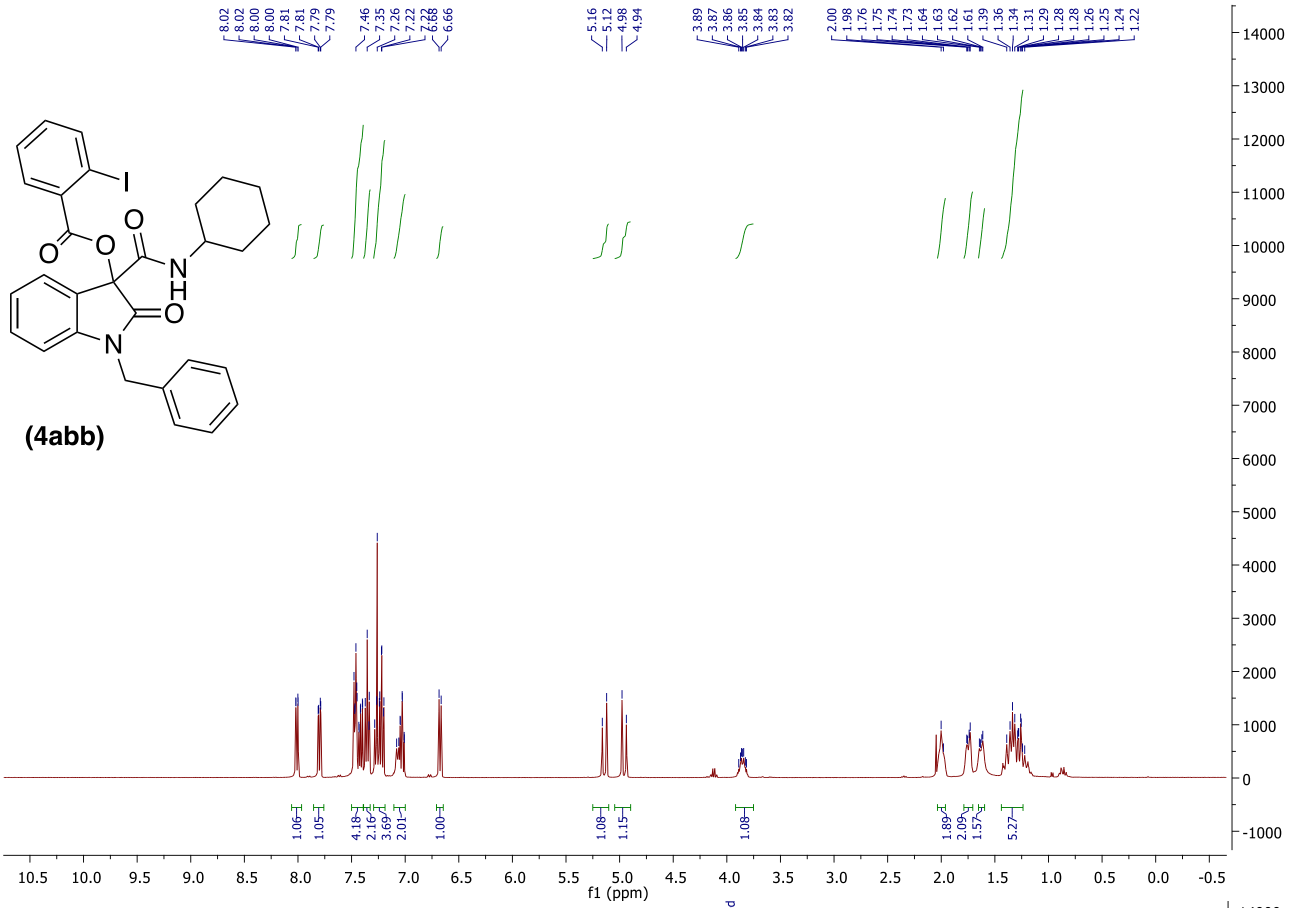

(4abb)

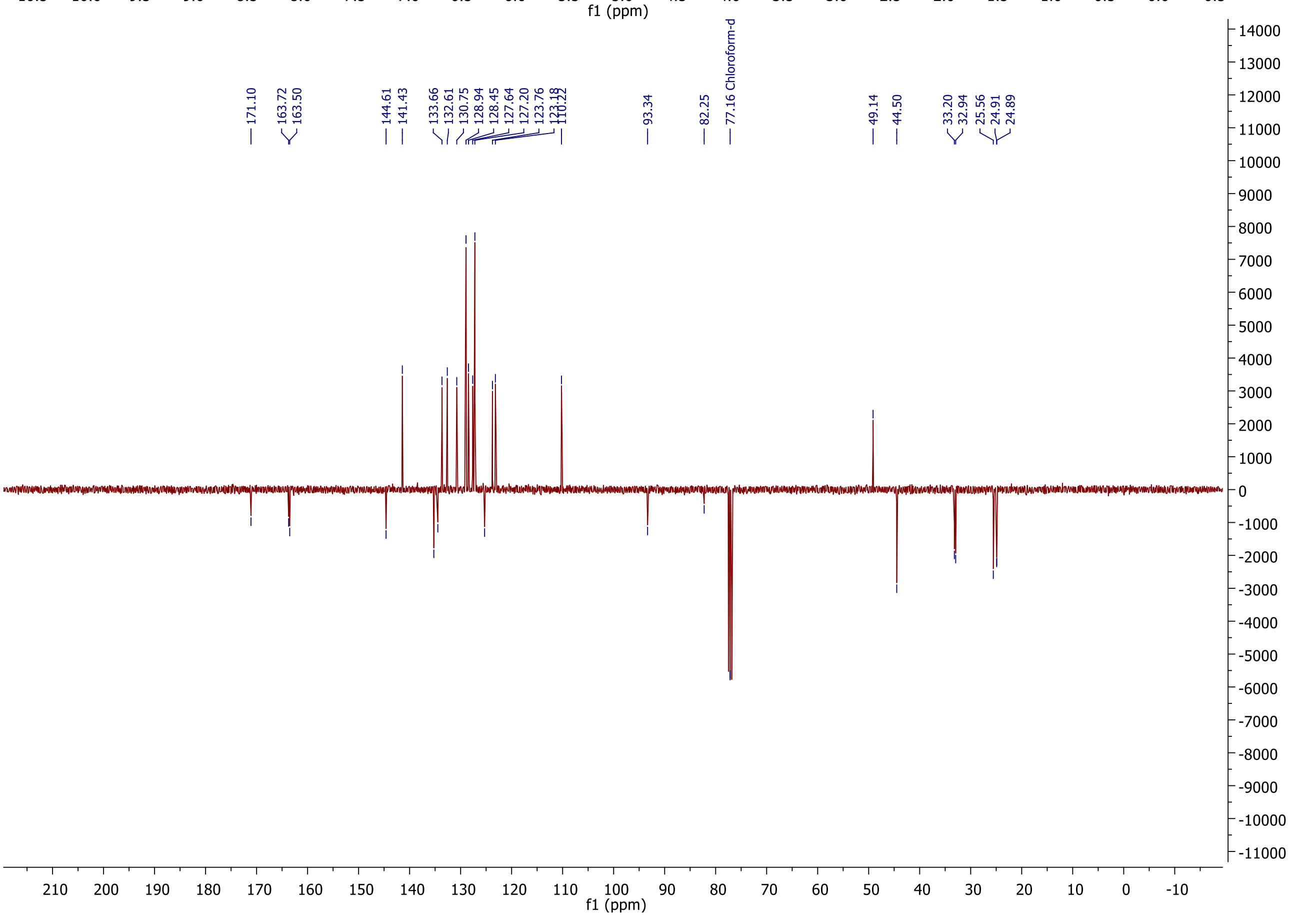

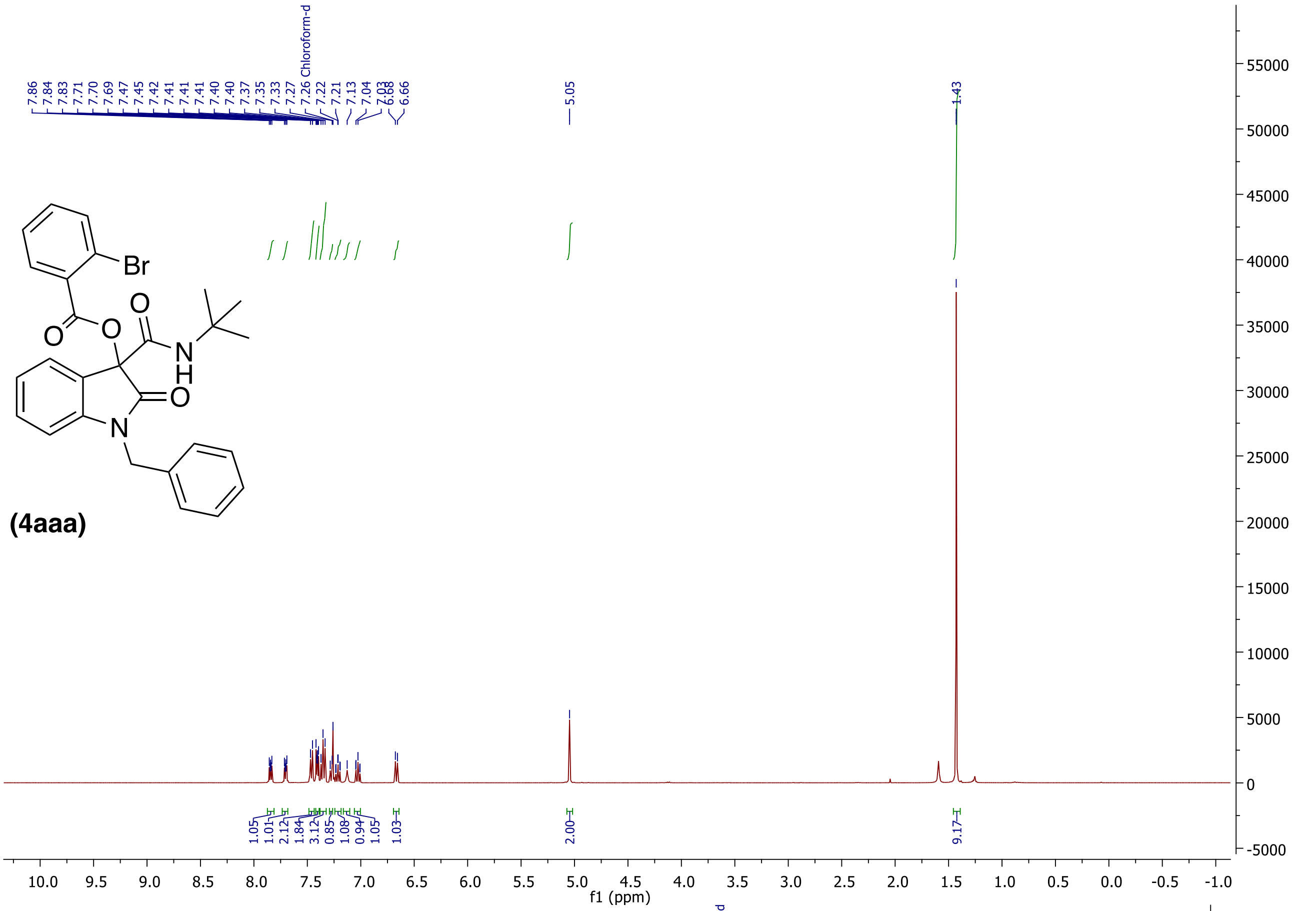
7.86
7.84
7.83
7.71
7.70
7.69
7.47
7.45
7.42
7.41
7.41
7.41
7.40
7.40
7.37
7.35
7.33
7.27
7.26 Chloroform-d
7.22
7.21
7.13
7.04
7.03
6.68
6.66
5.05
1.43
Br
O
O
O
N
H
O
N
1.05
1.01
2.12
1.84
3.12
0.85
1.08
0.94
1.05
1.03
2.00
9.17
f1 (ppm)

(4aaa)

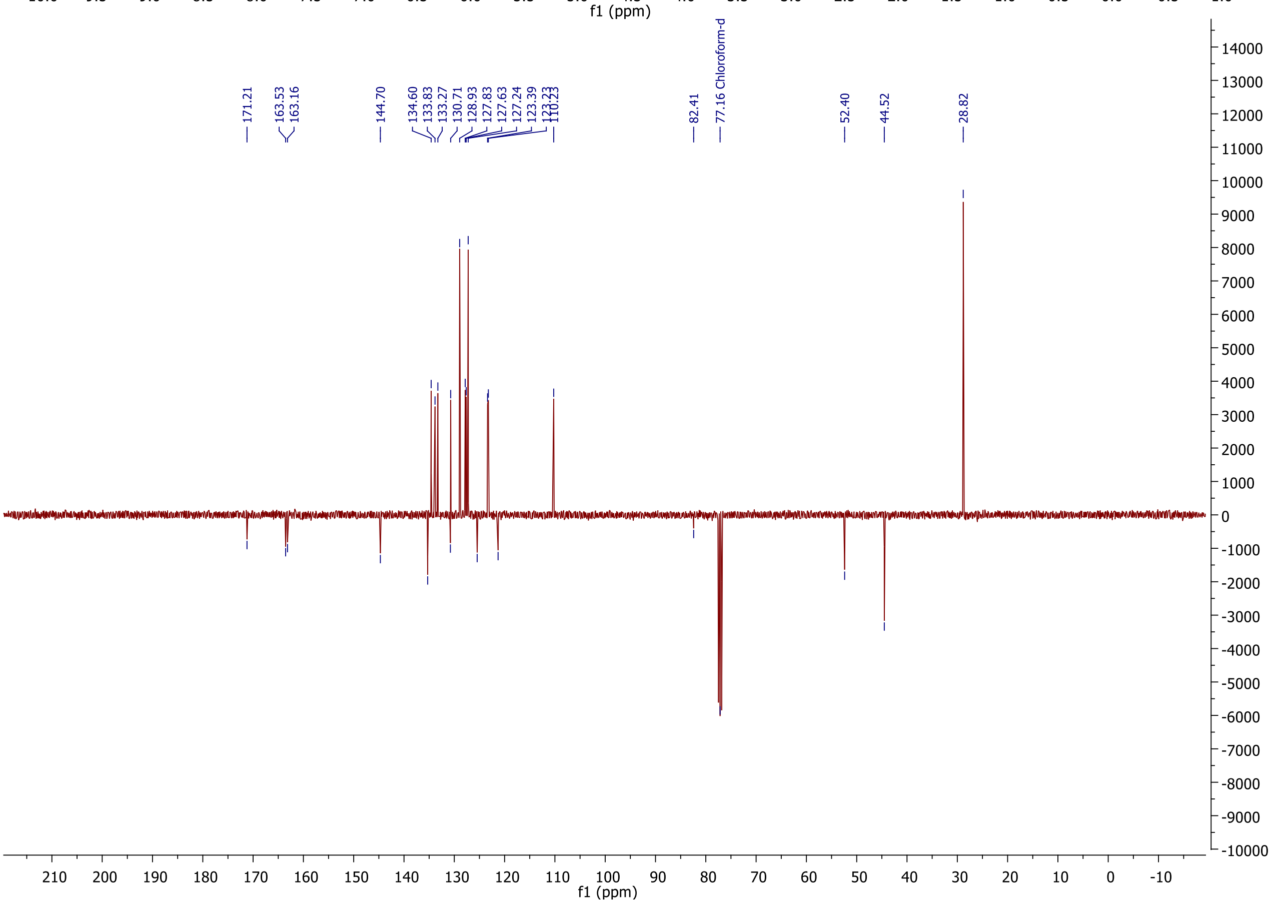
171.21
163.53
163.16
144.70
134.60
133.83
133.27
130.71
128.93
127.83
127.63
127.24
123.39
123.23
110.23
82.41
77.16 Chloroform-d
52.40
44.52
28.82
f1 (ppm)

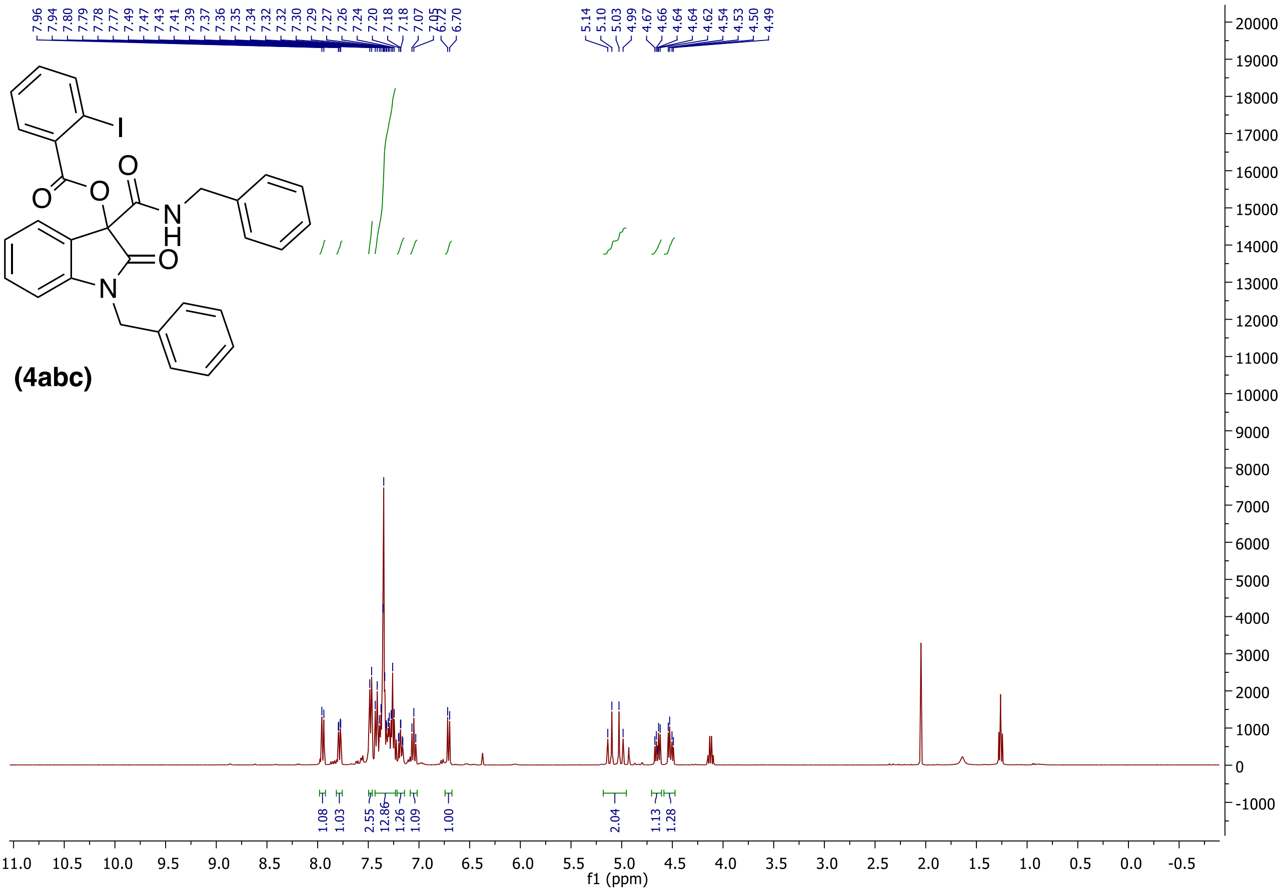
7.96 7.94 7.80 7.79 7.78 7.77 7.49 7.47 7.43 7.41 7.39 7.37 7.36 7.35 7.34 7.32 7.32 7.30 7.29 7.27 7.26 7.24 7.20 7.18 7.18 7.07 7.05 6.72 6.70
5.14 5.10 5.03 4.99 4.67 4.66 4.64 4.64 4.62 4.54 4.53 4.50 4.49
(4abc)
1.08 1.03 2.55 12.86 1.26 1.09 1.00 2.04 1.13 1.28
f1 (ppm)

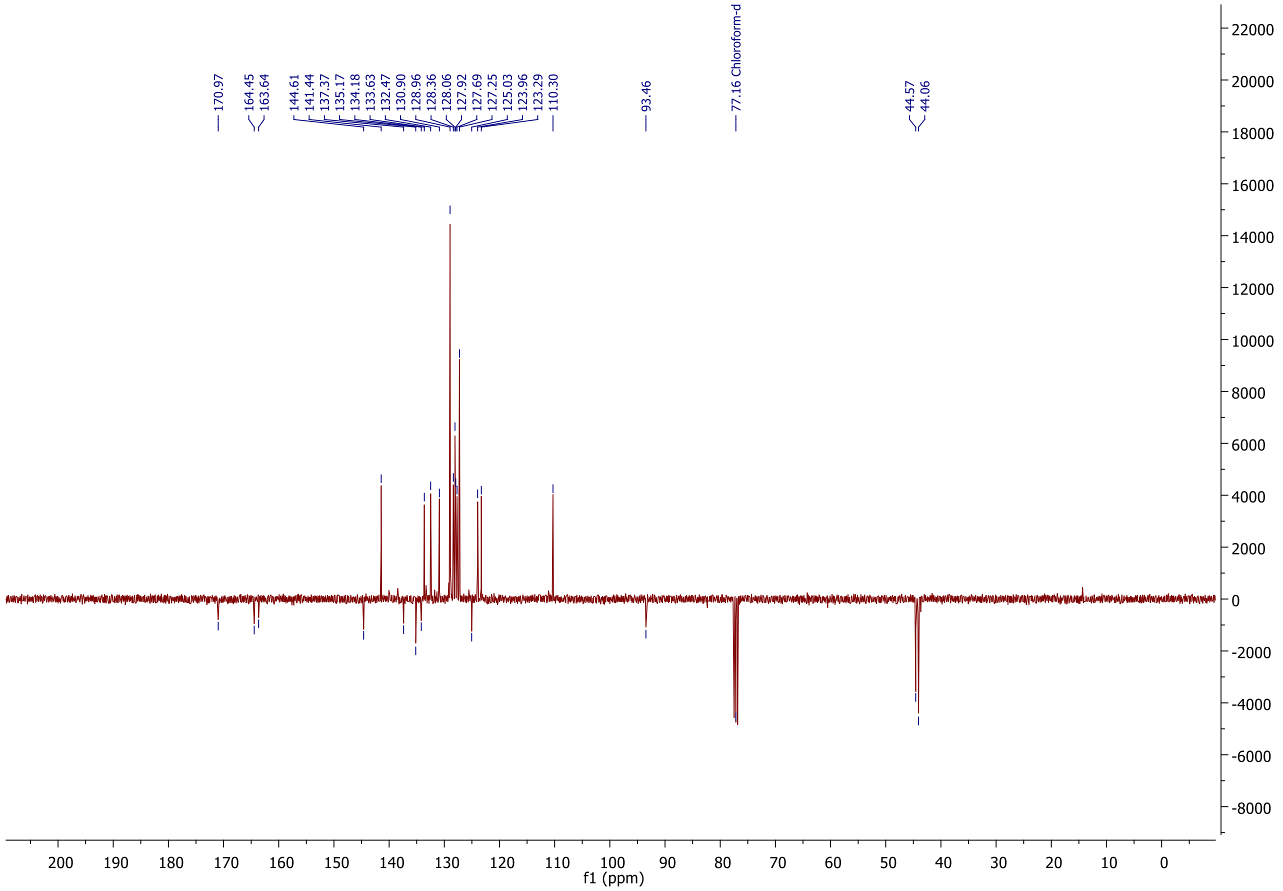
170.97 164.45 163.64 144.61 141.44 137.37 135.17 134.18 133.63 132.47 130.90 128.96 128.36 128.06 127.92 127.69 127.25 125.03 123.96 123.29 110.30 93.46 77.16 Chloroform-d 44.57 44.06
f1 (ppm)

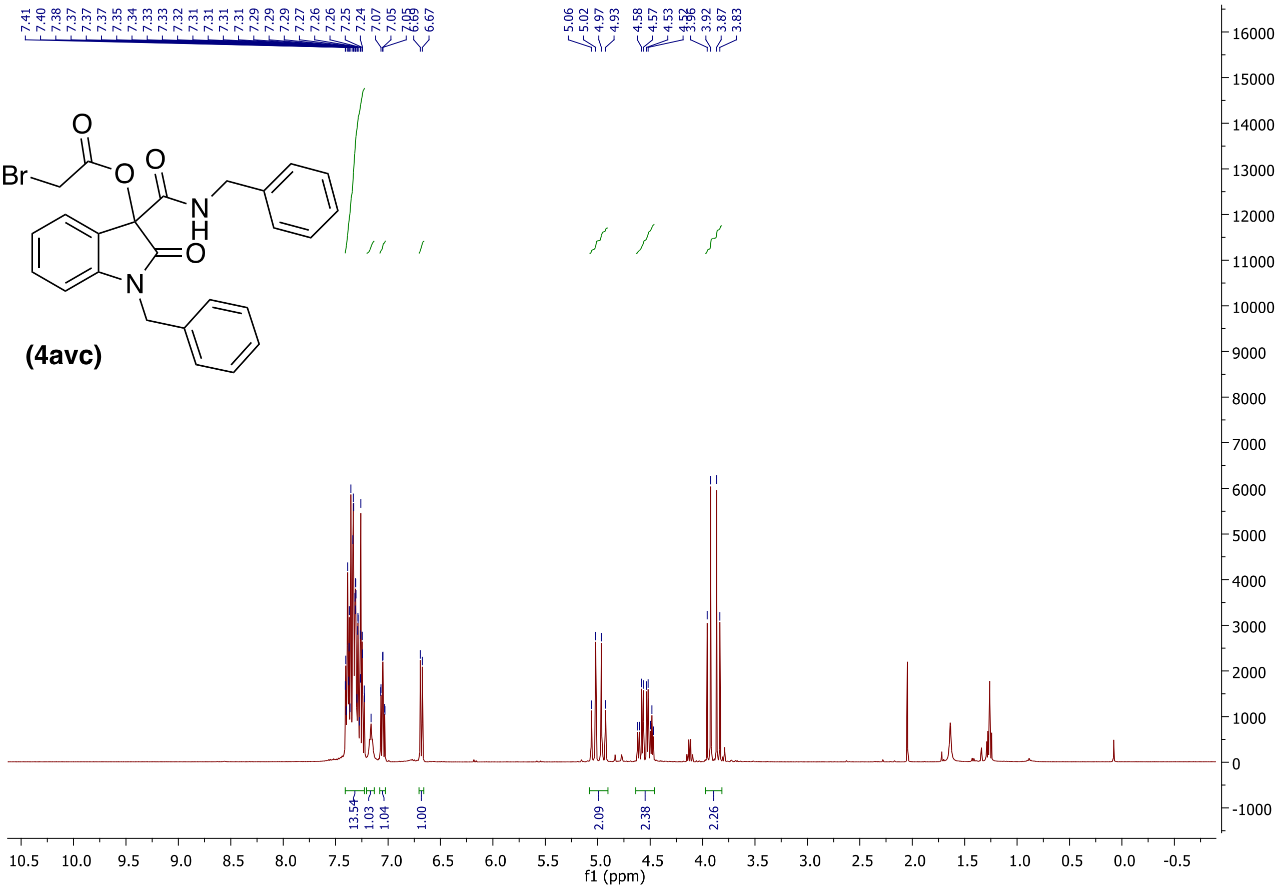

7.41
7.40
7.38
7.37
7.37
7.37
7.35
7.34
7.33
7.33
7.32
7.31
7.31
7.31
7.29
7.29
7.29
7.27
7.26
7.26
7.25
7.24
7.07
7.05
6.69
6.67
5.06
5.02
4.97
4.93
4.58
4.57
4.53
4.52
3.96
3.92
3.87
3.83
Br
O
O
O
N
H
O
N
13.54
1.03
1.04
1.00
2.09
2.38
2.26
f1 (ppm)

(4avc)

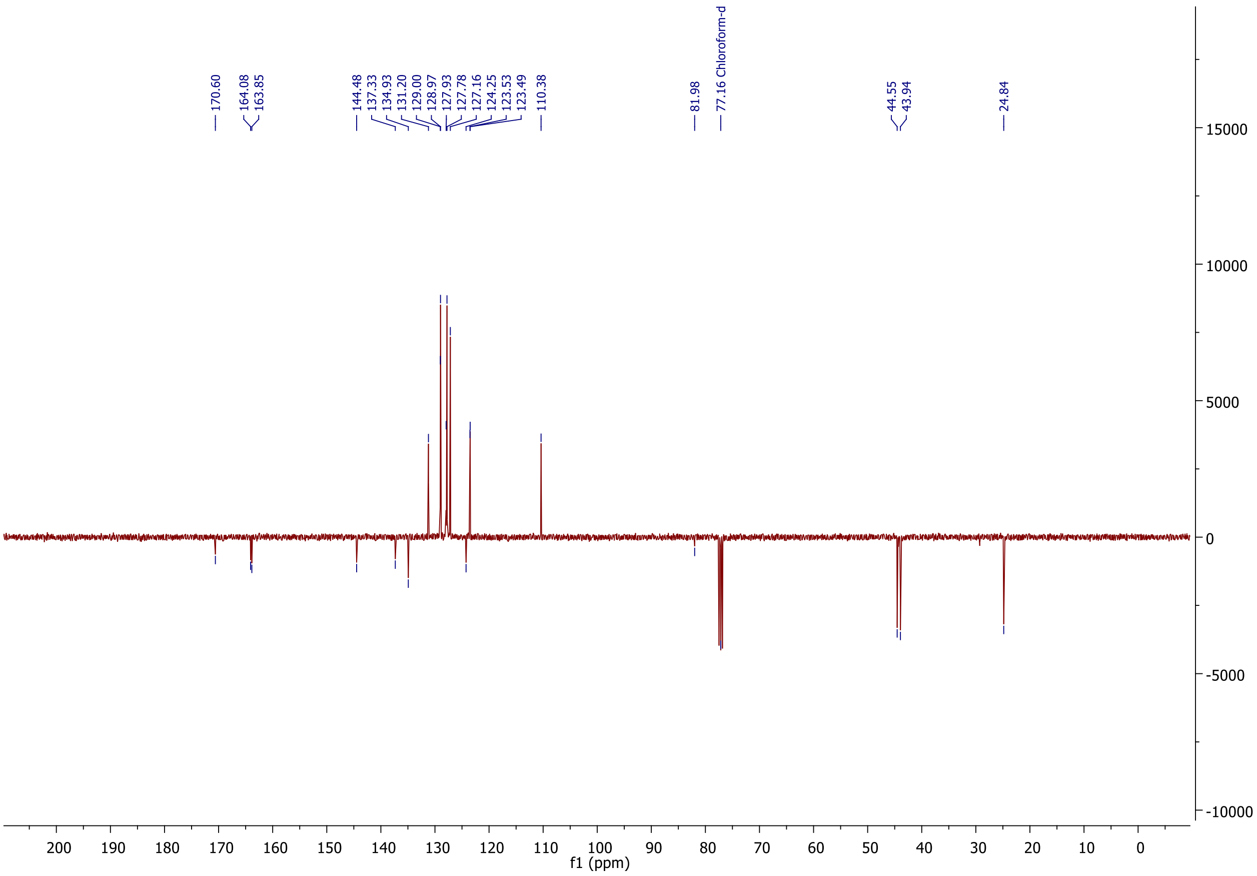

170.60
164.08
163.85
144.48
137.33
134.93
131.20
129.00
128.97
127.93
127.78
127.16
124.25
123.53
123.49
110.38
81.98
77.16 Chloroform-d
44.55
43.94
24.84
f1 (ppm)

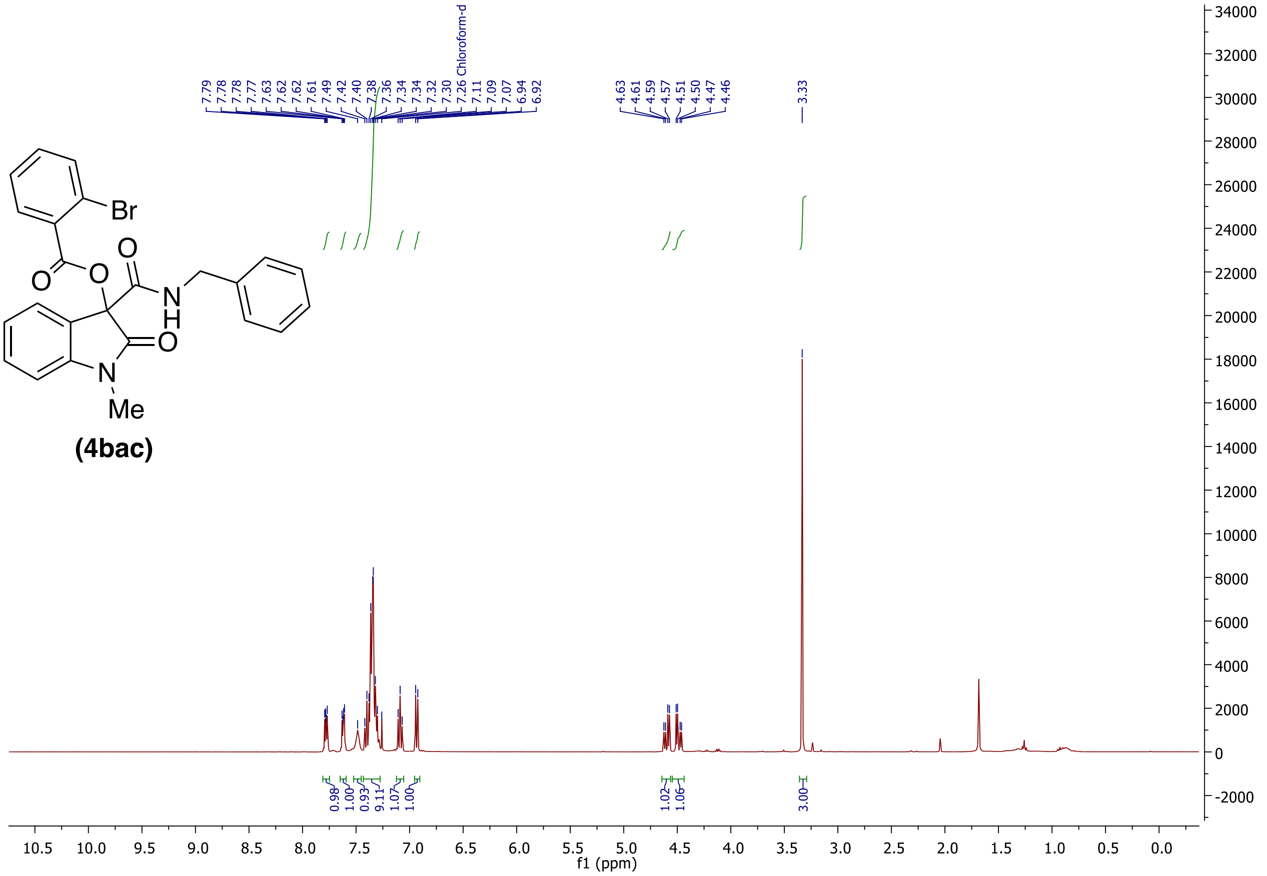

(4bac)

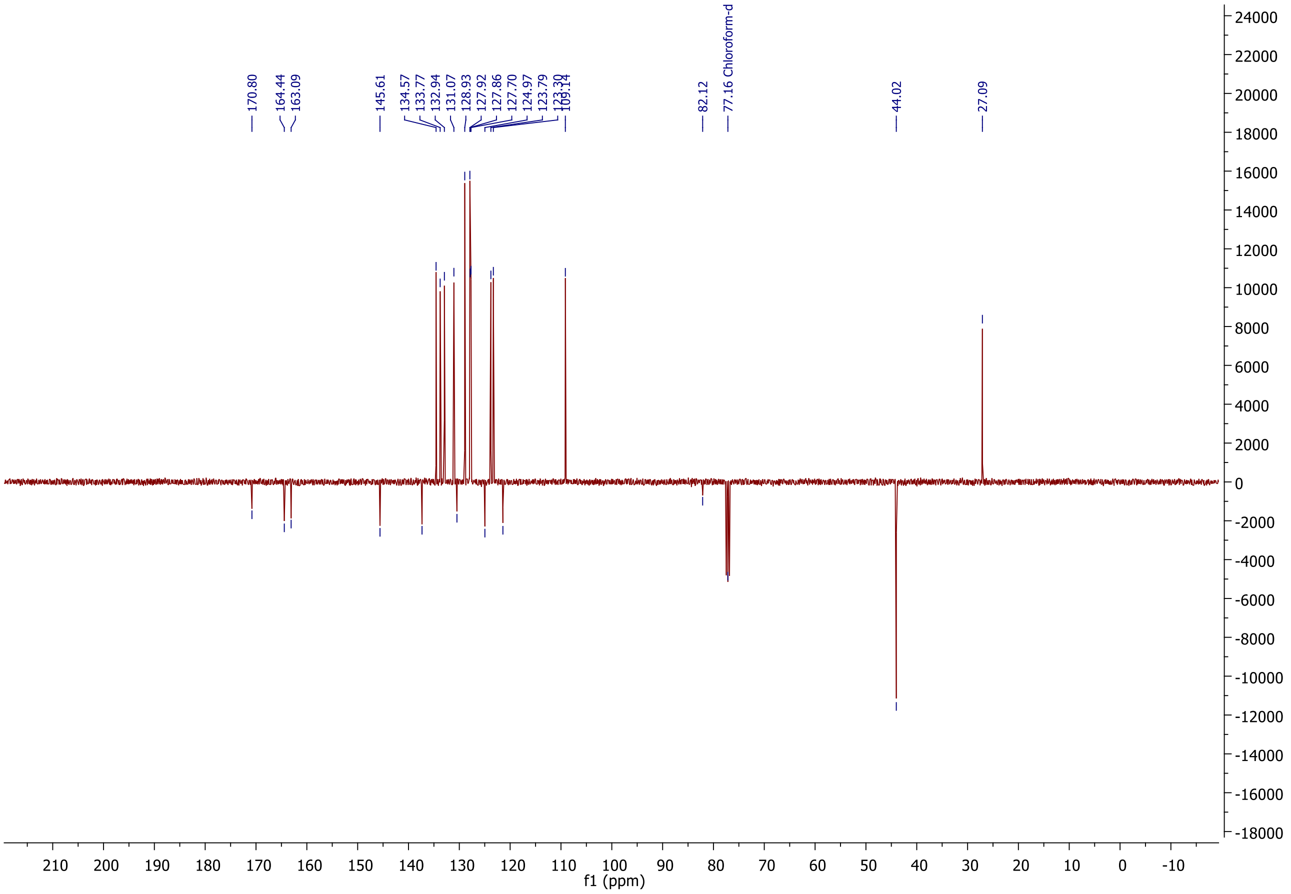

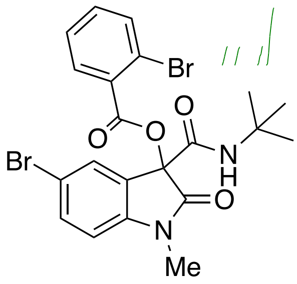

(4faa)

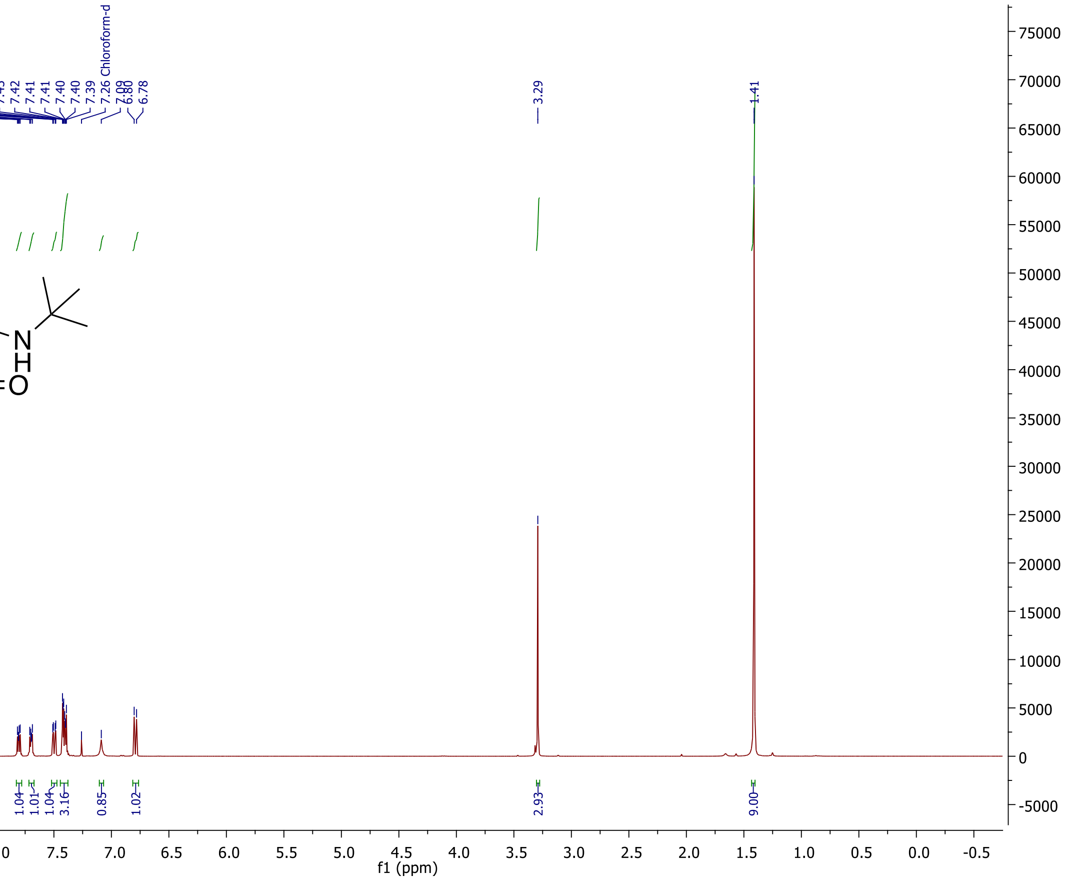
7.82
7.81
7.80
7.79
7.71
7.70
7.69
7.51
7.50
7.49
7.48
7.43
7.42
7.41
7.41
7.40
7.40
7.39
7.26 Chloroform-d
6.78
3.29
1.41
1.04
1.01
1.04
3.16
0.85
1.02
2.93
9.00
f1 (ppm)

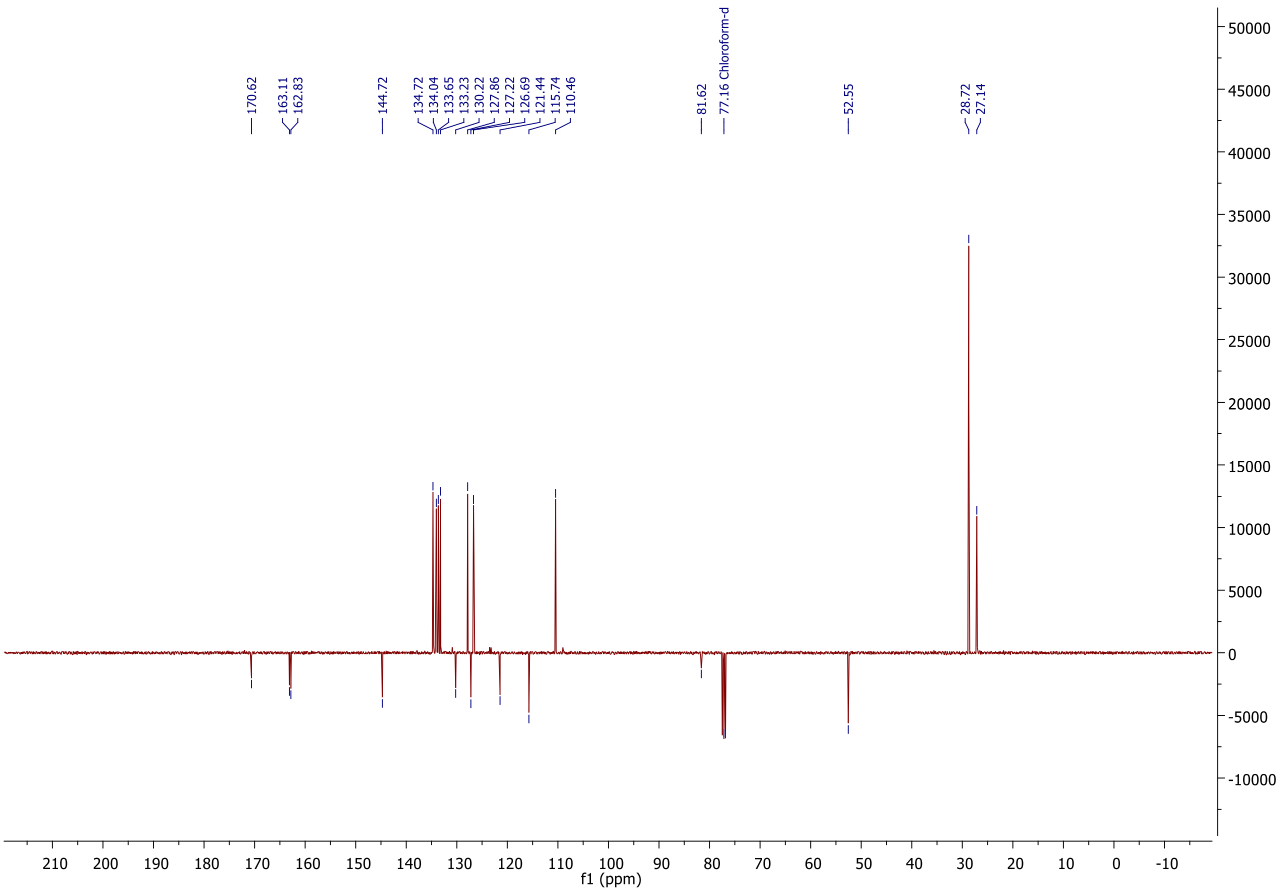
170.62
163.11
162.83
144.72
134.72
134.04
133.65
133.23
130.22
127.86
127.22
126.69
121.44
115.74
110.46
81.62
77.16 Chloroform-d
52.55
28.72
27.14
f1 (ppm)

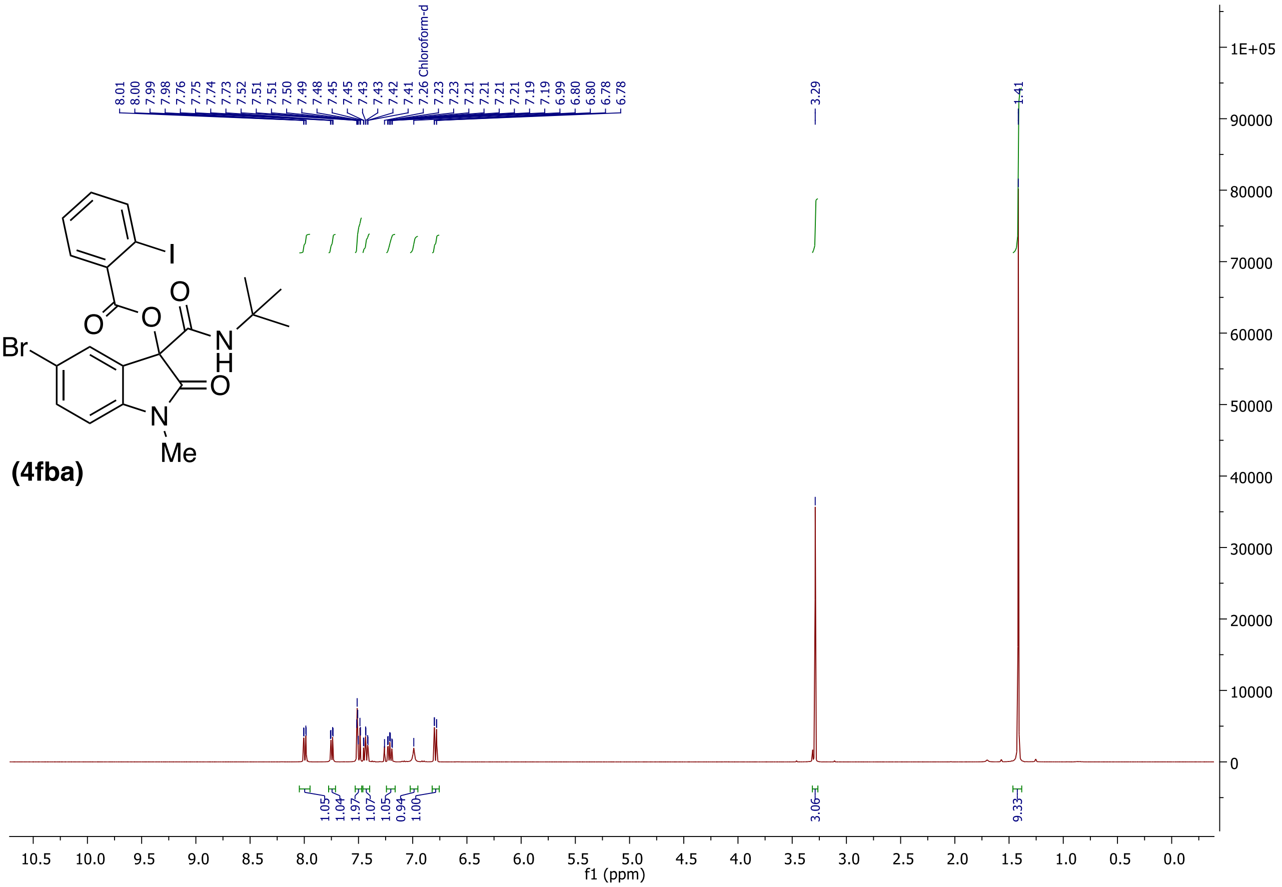

(4fba)

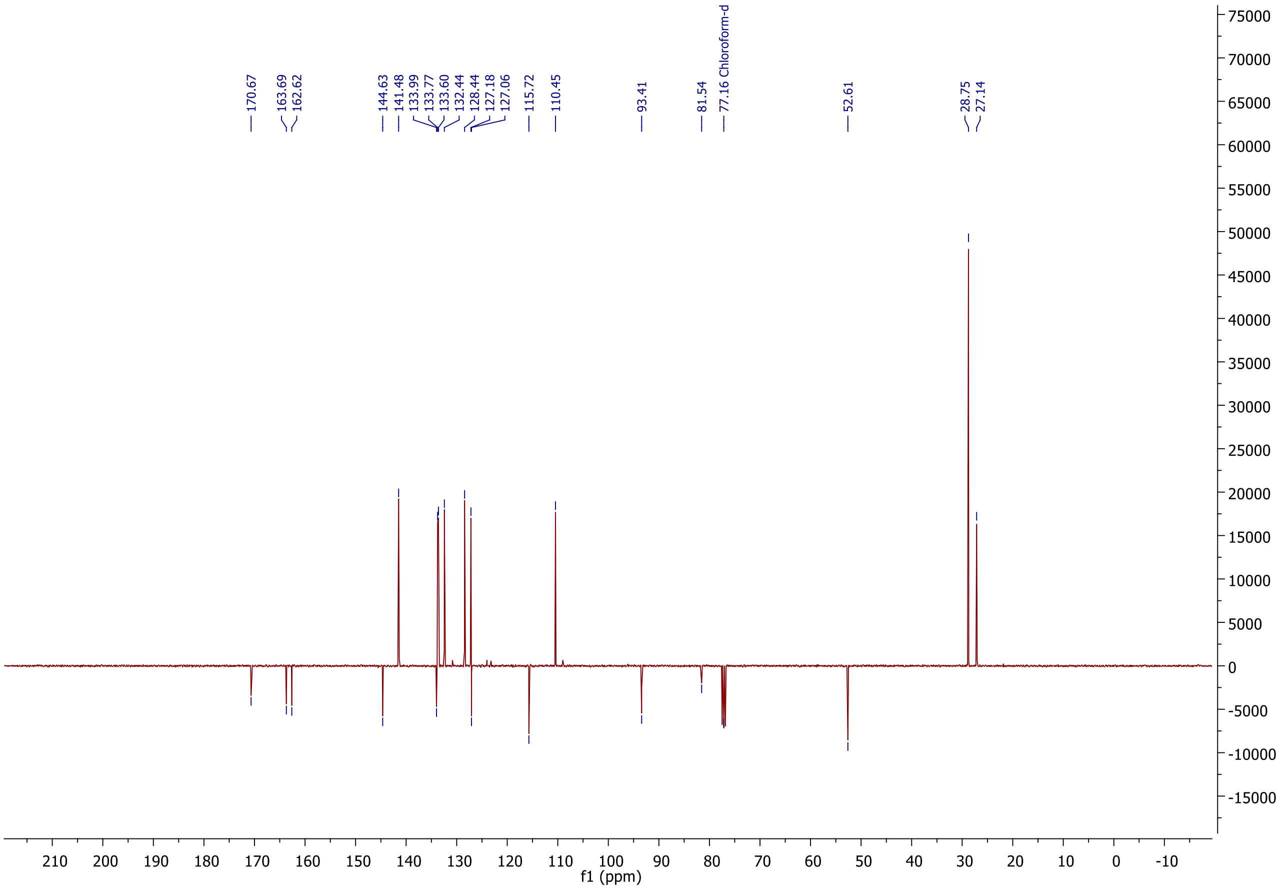

^{1}H and ^{13}C NMR spectra

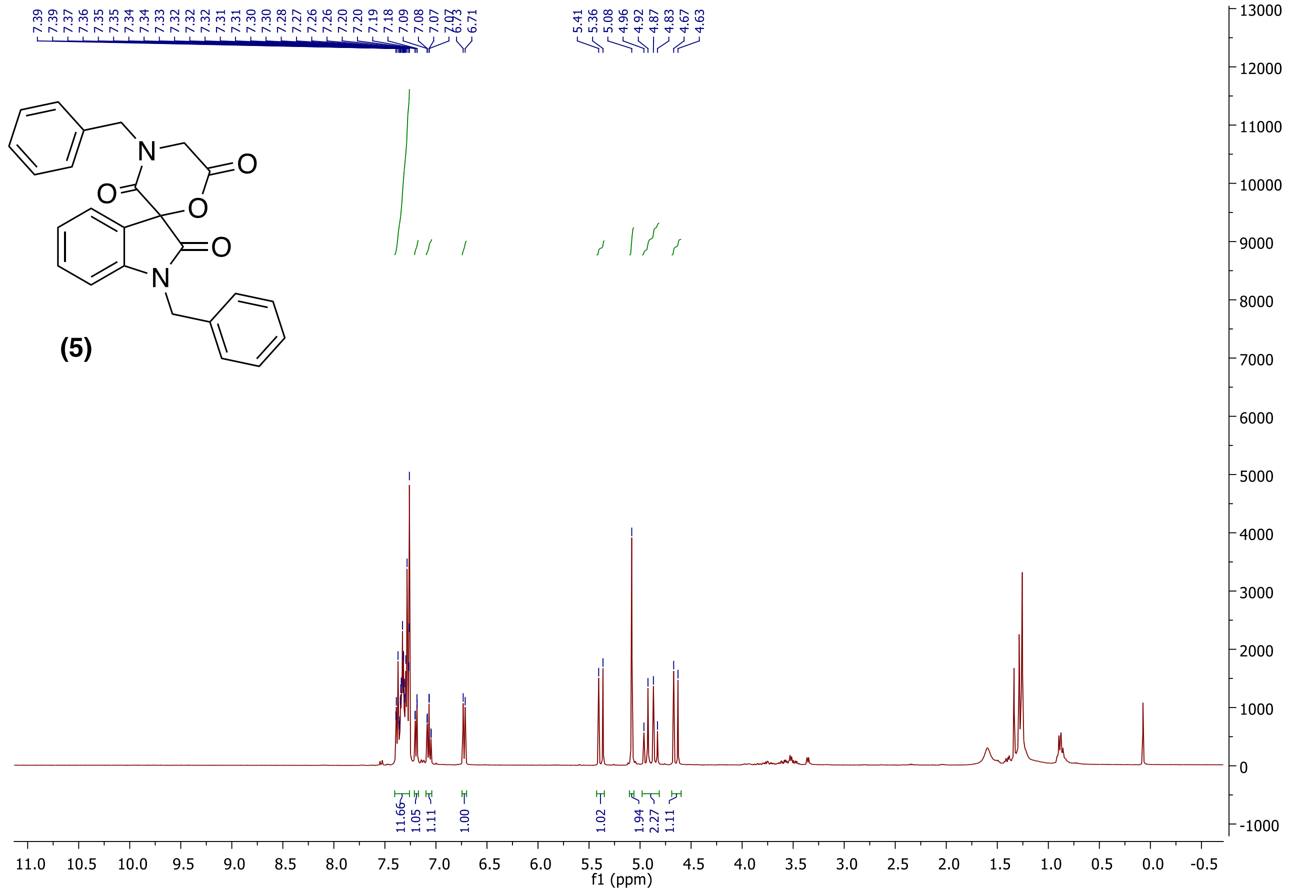

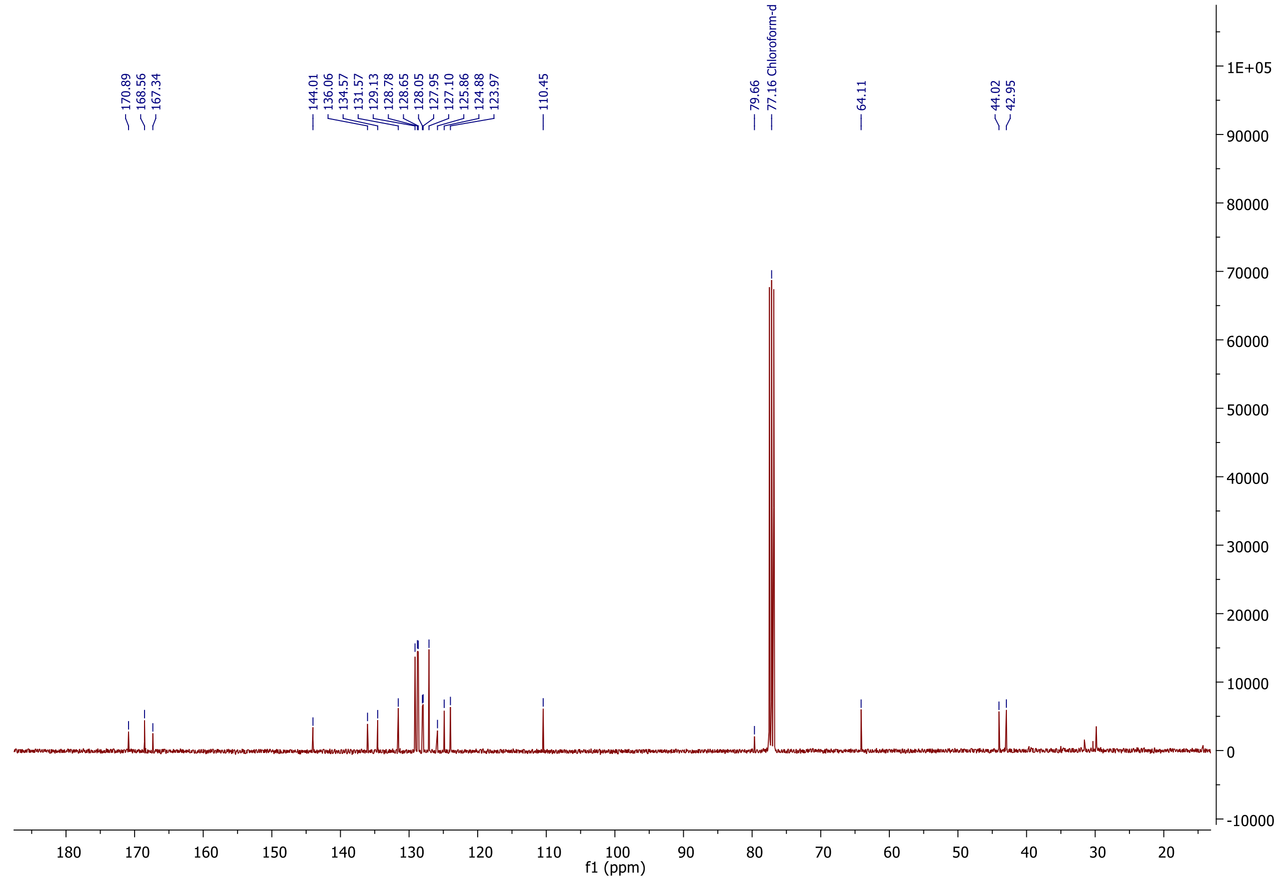

^{1}H and ^{13}C NMR spectra

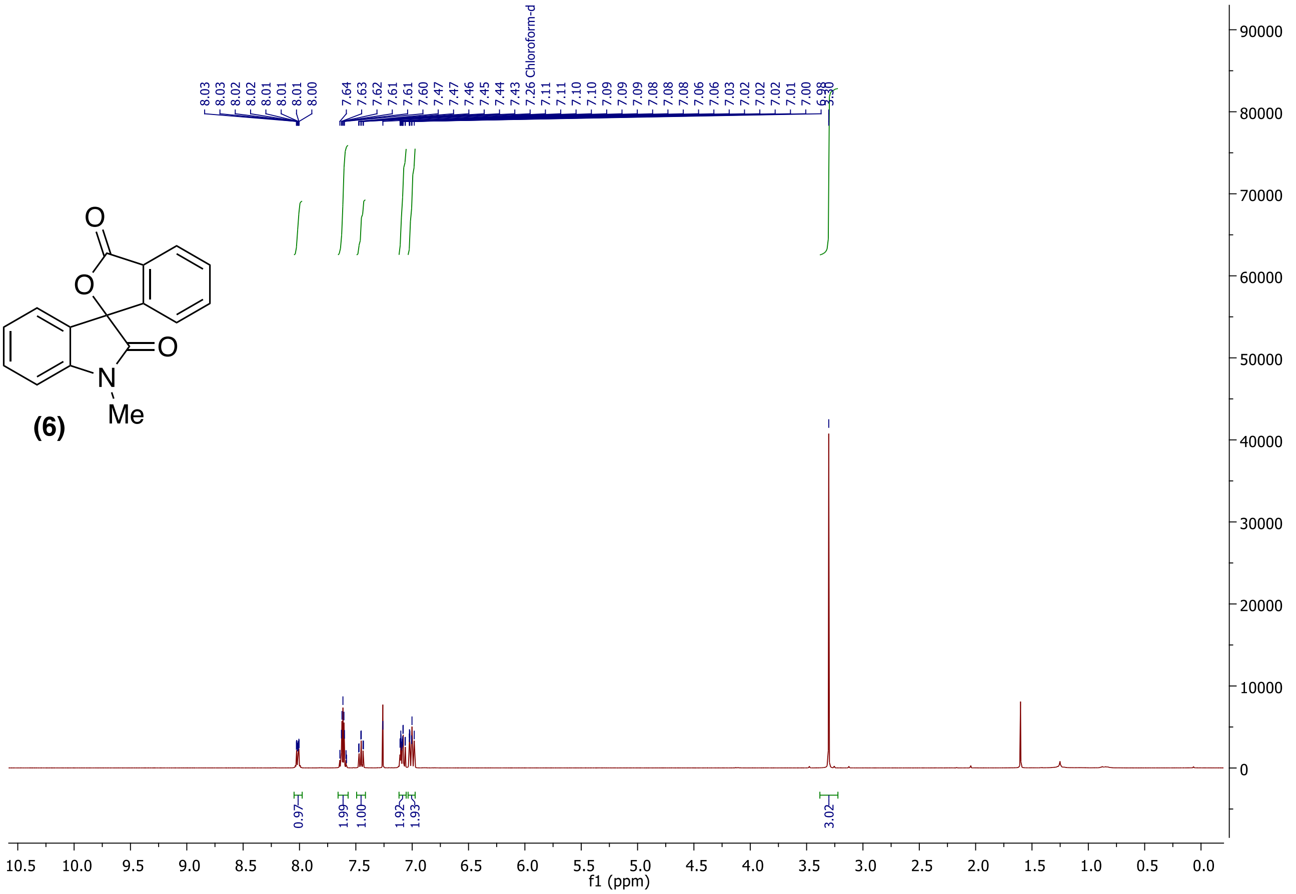

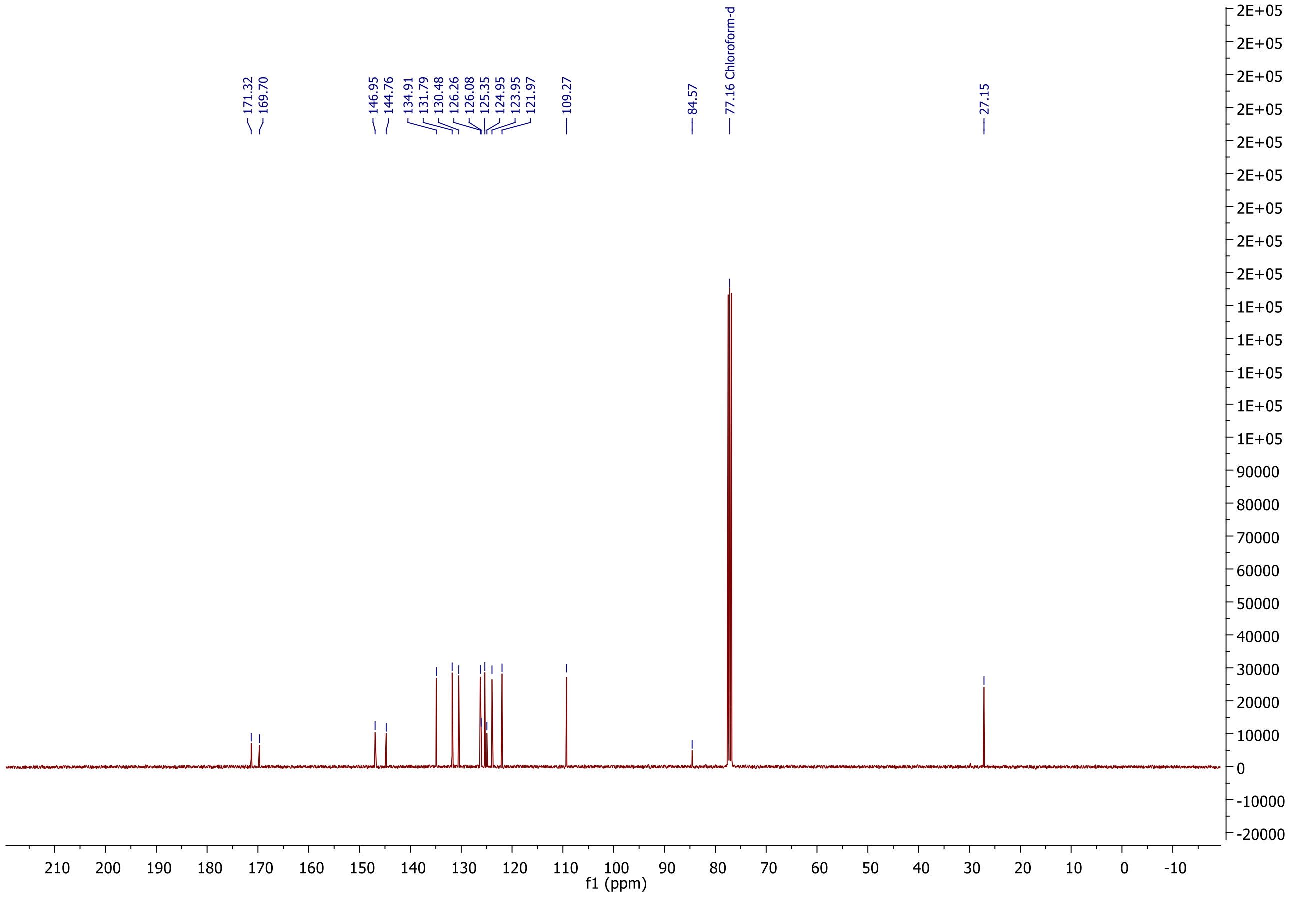

^1H and ^{13}C NMR spectra

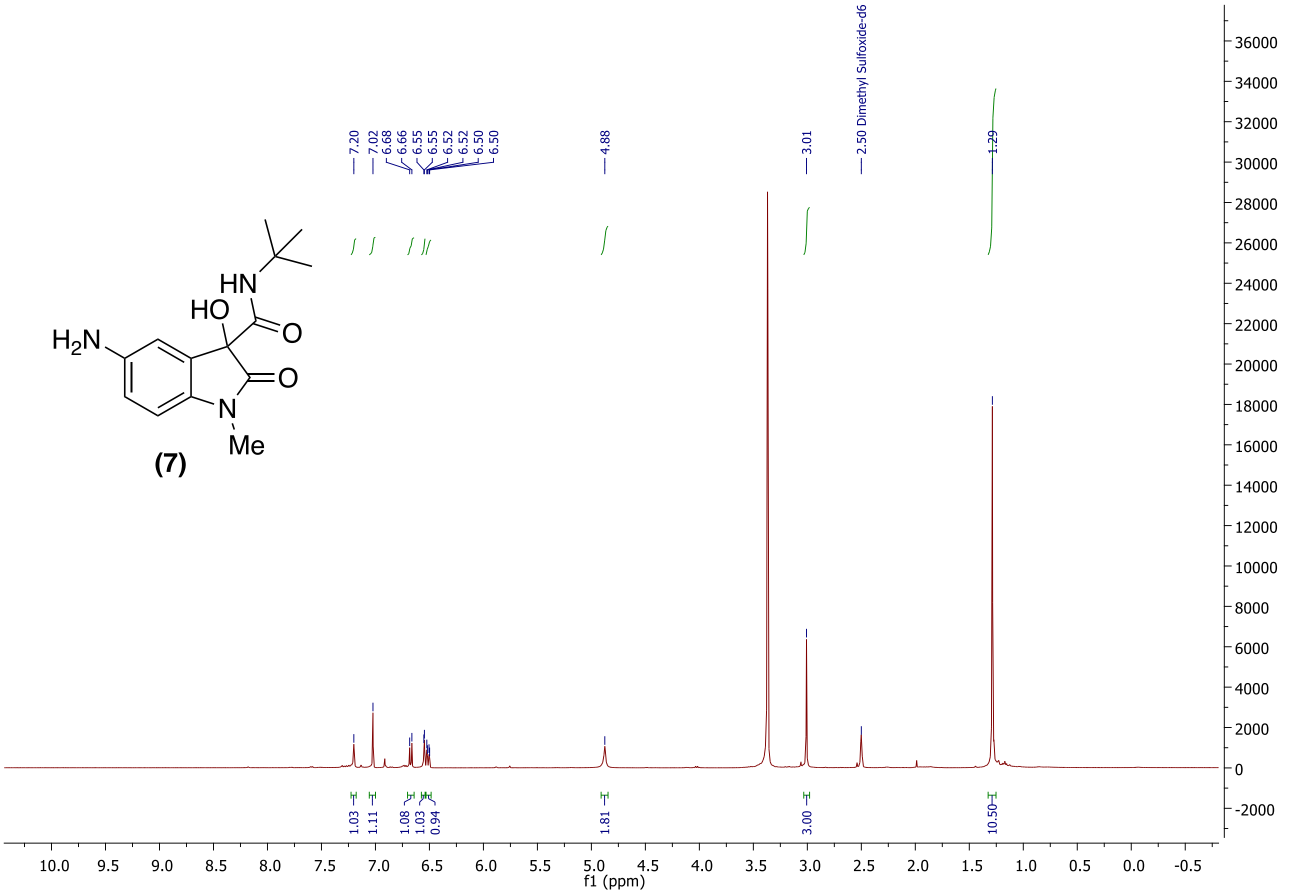

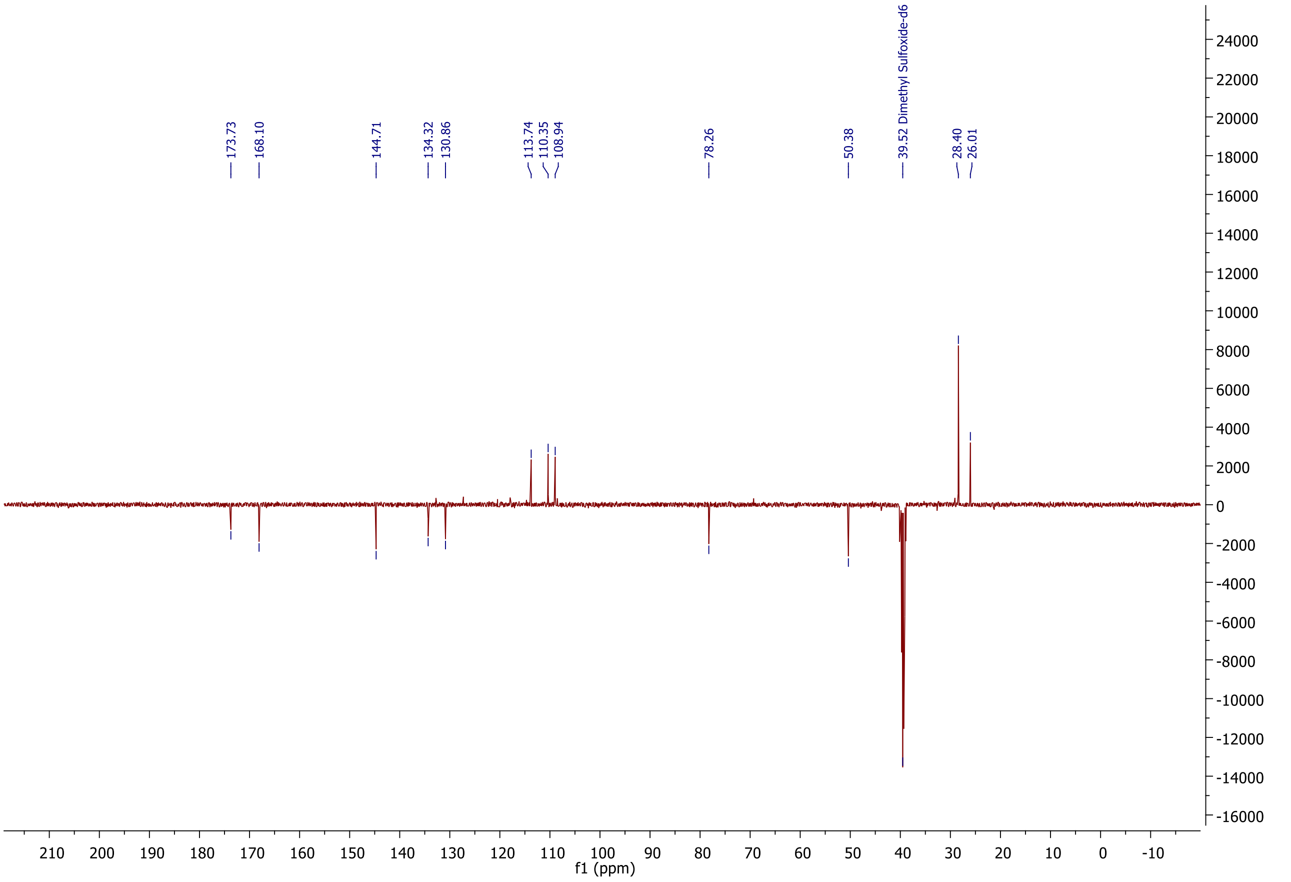

Supplement: Supplementary file 1 [file molecules-29-05538-s001.zip › molecules-3300815-supplementary.pdf]
